# Lapatinib Plasma and Tumor Concentrations and Effects on HER Receptor Phosphorylation in Tumor

**DOI:** 10.1371/journal.pone.0142845

**Published:** 2015-11-16

**Authors:** Neil L. Spector, Faith C. Robertson, Sarah Bacus, Kimberly Blackwell, Deborah A. Smith, Kelli Glenn, Leanne Cartee, Jennifer Harris, Carie L. Kimbrough, Mark Gittelman, Eli Avisar, Peter Beitsch, Kevin M. Koch

**Affiliations:** 1 Department of Medicine, Duke Cancer Center, Duke University Medical Center, Durham, North Carolina, United States of America; 2 Targeted Molecular Diagnostics/Quintiles, Westmont, Illinois, United States of America; 3 GlaxoSmithKline, Research Triangle Park, North Carolina, United States of America; 4 Pivot Oncology Consulting, Durham, North Carolina, United States of America; 5 PAREXEL International, Durham, North Carolina, United States of America; 6 Breast Cancer Specialists, Allentown, Pennsylvania, United States of America; 7 Department of Surgery, University of Miami School of Medicine, Miami, Florida, United States of America; 8 Dallas Surgical Group, Dallas, Texas, United States of America; Fondazione IRCCS Istituto Nazionale dei Tumori, ITALY

## Abstract

**Purpose:**

The paradigm shift in cancer treatment from cytotoxic drugs to tumor targeted therapies poses new challenges, including optimization of dose and schedule based on a biologically effective dose, rather than the historical maximum tolerated dose. Optimal dosing is currently determined using concentrations of tyrosine kinase inhibitors in plasma as a surrogate for tumor concentrations. To examine this plasma-tumor relationship, we explored the association between lapatinib levels in tumor and plasma in mice and humans, and those effects on phosphorylation of human epidermal growth factor receptors (HER) in human tumors.

**Experimental Design:**

Mice bearing BT474 HER2+ human breast cancer xenografts were dosed once or twice daily (BID) with lapatinib. Drug concentrations were measured in blood, tumor, liver, and kidney. In a randomized phase I clinical trial, 28 treatment-naïve female patients with early stage HER2+ breast cancer received lapatinib 1000 or 1500 mg once daily (QD) or 500 mg BID before evaluating steady-state lapatinib levels in plasma and tumor.

**Results:**

In mice, lapatinib levels were 4-fold higher in tumor than blood with a 4-fold longer half-life. Tumor concentrations exceeded the *in vitro* IC_90_ (~ 900 nM or 500 ng/mL) for inhibition of HER2 phosphorylation throughout the 12-hour dosing interval. In patients, tumor levels were 6- and 10-fold higher with QD and BID dosing, respectively, compared to plasma trough levels. The relationship between tumor and plasma concentration was complex, indicating multiple determinants. HER receptor phosphorylation varied depending upon lapatinib tumor concentrations, suggestive of changes in the repertoire of HER homo- and heterodimers.

**Conclusion:**

Plasma lapatinib concentrations underestimated tumor drug levels, suggesting that optimal dosing should be focused on the site of action to avoid to inappropriate dose escalation. Larger clinical trials are required to determine optimal dose and schedule to achieve tumor concentrations that maximally inhibit HER receptors.

**Trial Registration:**

Clinical Trial Registration: NCT00359190

## Introduction

Cancer treatment has historically been based on the use of non-specific cytotoxic chemotherapies that were selected based on their ability to disrupt global cell processes that promote tumor growth and survival, e.g. DNA repair and replication [1,2]. However, these processes are not only critical to maintaining the viability of tumor cells, but also that of highly proliferative non-malignant cells in bone marrow, oral mucosa, and the gastrointestinal tract. Manipulating the dose confers some selectivity, but an inherent lack of specificity (narrow therapeutic index) led to dose selection based on the identification of a maximum tolerated dose (MTD). Consequently, there are examples where patients were subjected to life-threatening toxicity without significant improvement in clinical outcome [3].

Over the past two decades, advancements have been made in our understanding of the molecular biology of cancer cells including identification of oncogenic drivers that promote tumorigenesis and disease progression in solid tumors and hematological malignancies [4, 5]. These discoveries have prompted the development of small molecule and antibody-based therapies designed to selectively target deregulated signaling pathways in tumor, thereby minimizing toxicity to normal tissues [6,7].

The paradigm shift from cytotoxic drugs to more specific targeted therapies poses new challenges, including whether dose selection should continue to be based on an MTD, which maximizes tolerability, or alternatively, a biologically effective dose (BED) which maximizes efficacy [8]. Dose escalation beyond the BED can result in toxicity without additional clinical benefit, at least not mediated via inhibition of the intended target(s).

Lapatinib, a small molecule inhibitor of the family of Human Epidermal Growth Factor Receptor 1 (HER1 or EGFR) and HER2 oncogenic receptor tyrosine kinases, is approved for the treatment of advanced stage HER2-overexpressing (HER2+) breast cancers. Lapatinib has been shown to be highly specific for its intended targets at concentrations as high as 10–26 μM [9,10]. An MTD for lapatinib and many other targeted therapies has not been achieved in the clinic. Instead, selection of a biologically effective dose for targeted therapies to pursue in later phase clinical trials has frequently been based on the identification of a dose shown to achieve plasma concentrations greater than an IC_90_, the concentration of drug required to inhibit *in vitro* proliferative growth of tumor cell lines by 90% [11]. Once daily dosing of 1250 mg lapatinib, the approved dose in combination with capecitabine, achieves minimum steady-state plasma concentrations in the low μΜ range throughout most of the dosing interval [12], which exceeds the IC_90_ (~900 nM or 500 ng/mL) for lapatinib in multiple HER2+ human breast cancer cell lines [10]. There is, however, very little data on the concentration of active drug in tumor tissue, which may or may not reflect concentrations in plasma, and is presumably more directly related to the antitumor effect. In this study, plasma concentrations of lapatinib were shown to markedly underestimate those concomitantly achieved in tumor tissue. These data and their impact on the activation of HER receptors in lapatinib-treated tumors are discussed.

## Methods

### Mouse Study

CB-17 SCID female mice (4–6 weeks old) were purchased from Charles River Laboratories (Wilmington, MA). All animal studies were conducted after review by the Institutional Animal Care and Use Committee at GSK and in accordance with the GSK Policy on the Care, Welfare and Treatment of Laboratory Animals. The Institutional Animal Care and Use Committee at GSK specifically approved these studies (IACUC #01APK0033). All animals were euthanized in accord with the 2013 AVMA Guidelines for the Euthanasia of Animals. CO_2_ was delivered by gradual displacement of 20% chamber volume/minute using a flow meter per an approved GSK IACUC protocol and followed by cervical dislocation to insure death. The HER2+ BT474 human breast cancer cell line/xenograft is a well-established model of human HER2+ breast cancer, the population for which the drug is approved. The BT474 cell line was obtained November 2001 from the ATCC (American Tissue Type Culture Collection, Manassas VA) (ATCC Number HTB-20), and its authenticity was confirmed by demonstrating overexpression of HER2 protein, and expression of other markers of human breast cancer (e.g., Estrogen Receptor). Tumor xenografts were initiated by subcutaneous implantation of tumor fragments (20–100 mg) from established tumors in the right flank as previously described [10]. Tumors were measured with calipers, and mice were weighed twice weekly. Treatment with lapatinib began when tumors were ~200 mm^3^. Forty-five mice were treated with lapatinib at 100 mg/kg every 12 hours (6 doses), and another 45 were treated at a dose of 200 mg/kg every 24 hours (3 doses). The 100 mg/kg BID dose has been shown to be effective in treating HER2+ breast cancer xenografts [10]. Lapatinib was given as an oral suspension in a vehicle consisting of water with 0.5% hydroxymethylcellulose and 0.1% Tween-80. Blood, tumor, liver, and kidney samples were collected after the last doses at various times up to 6 days (n = 3 at each time point from each treatment group). Blood (approximately 0.5 mL per sample) was collected by cardiac puncture and placed into tubes containing EDTA as the anticoagulant. After the blood samples were collected, mice were sacrificed and the following tissues removed: tumor, liver, and kidney. Animals were euthanized as described above upon completion of the study. Blood and tissues were collected at 1, 4, 10, 24, 48, 72, 96, 144 hours following the last dose for analysis of lapatinib.

### Clinical Study

**Fig 1:**
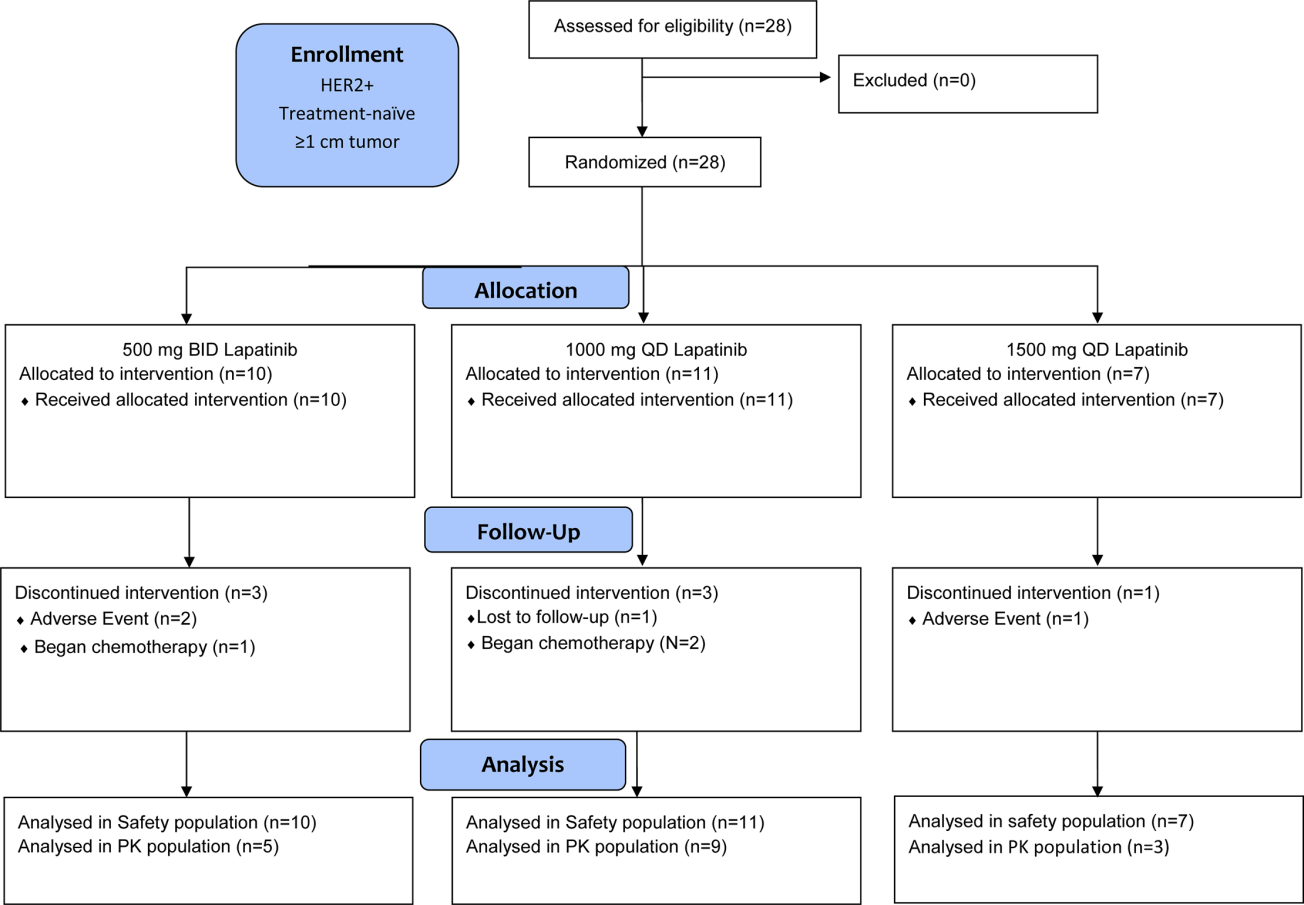
Schema of EGF10027. Twenty-eight patients were randomized to the following treatment groups: lapatinib 500 mg PO BID; lapatinib 1000 mg PO QD; lapatinib 1500 mg PO QD. Patient follow up and data analysis were conducted as indicated.

EGF10027 was a randomized, multicenter phase I clinical trial (NCT00359190) that examined the effects of different doses and schedules of lapatinib monotherapy on concentrations of lapatinib in plasma and tumor as well as the expression of total and phosphorylated forms of HER receptors in tumor tissue (Fig 1; S1 Protocol). Patients were accrued from June 2004 through June 2007, which was the data collection date for primary outcome measures. With follow up, the study completion date was January 2008. Eligible patients were females; age ≥18 years; with histologically confirmed, treatment-naïve invasive breast cancer ≥1cm; early stage, operable breast cancer; HER2 overexpression (by local laboratory) as determined by 3+ staining in 10% tumor cells by immunohistochemistry (IHC) or 1+ or 2+ staining by IHC and HER2 gene amplification by fluorescent-in-situ hybridization; Karnofsky performance status ≥ 70; left ventricular ejection fraction >50%; adequate bone marrow, hepatic and renal function; non-childbearing or child bearing with negative screening pregnancy test and agreed to use various pregnancy precautionary techniques. The following ethics committees/institutional review boards at each of the participating institutions approved the study: Duke University Health System, Durham NC; Institutional Review Board, University of Miami, Miami FL; Human Subjects Research Office, Western Institutional Review Board, Olympia WA; North Central Institutional Review Board at

Medical City, Dallas TX; Sacred Heart Hospital, Institutional Review Board, Allentown PA; Carillion Medical Center IRB, Roanoke VA; University of Pittsburgh Institutional Review Board, Pittsburgh PA; Institutional Review Board, Medical College of Wisconsin, Milwaukee WI; TriStar Nashville Market, Institutional Review Board, Nashville TN. Written informed consent was obtained from all participants prior to study participation. The study was conducted in accordance with the principles of the Declaration of Helsinki and International Conference on Harmonization Guidelines for Good Clinical Practice.

Eligible patients were randomly assigned to receive oral lapatinib at 1000 mg once-daily (QD), 1500 mg QD or 500 mg twice-daily (BID). Patients received lapatinib for ≥9 days prior to definitive surgery (mastectomy, lumpectomy). Post-treatment surgical resection was performed approximately 24 hours after the last lapatinib daily dose and 12 hours after the last BID dose. Tumor tissue obtained at resection was examined for biomarkers as described below and compared with the results from the pre-treatment biopsy. In addition, tumor obtained at the time of resection was examined for concentrations of lapatinib. Blood was drawn simultaneously for measurement of lapatinib plasma concentration.

### Lapatinib Bioanalysis

Mouse blood samples were treated with acetonitrile and spiked to contain stable labeled [^13^C2-, ^2^H3- and ^15^N1-] lapatinib as internal standard. After centrifugation, a portion of the blood supernatant was diluted with water and then evaluated on a liquid chromatography/mass spectrophotometry (LC/MS/MS) system (see below). Tissue samples (25 mg each) were also spiked with stable labeled lapatinib and then solubilized with 0.3 mL Solvable (Packard Biosciences; Meriden, CT) for 3–4 hours at 45°C. Lapatinib was extracted from the solubilized tissue with ethyl acetate. The ethyl acetate fractions were evaporated, and the dried samples were reconstituted in water:acetonitrile (75:25). Blood and tissue samples were evaluated on an LC/MS/MS system in which daughter ions of lapatinib and the stable-labeled internal standard were detected by positive-ion multiple reaction monitoring (MRM). An Oasis HLB column (1 x 50 mm) with a 100% aqueous mobile phase at a flow rate of 3mL/min was used for the extraction column. A Waters Symmetry C18 (2.1 x 50 mm, 3.5μm) column was used as the analytical column using acetonitrile/5mM ammonium acetate (10/90) at a flow rate of 0.4 mL/min. The range of the standard curve was 10–2000 ng/mL for both blood and tissues. The assay required a sample volume of 25μL to achieve the lower limit of quantitation (10 ng/mL). Quality control (QC) samples were analyzed with the study samples, and the QC data fell within the acceptable limits of the method. Data were acquired and analyzed with MassLynx v.3.3 (MicroMass Ltd).

Patient plasma samples were analyzed for lapatinib after protein precipitation with acetonitrile using a previously described LC/MS/MS method [13] with a 0.0086–8.6 μM (5–5000ng/mL) range of quantification, and precision and accuracy within 15%. Lapatinib concentrations in breast tumor tissue were determined by dissolving 50 mg tissue with sodium hydroxide:ethanol, followed by liquid-liquid extraction with methyl-t-butyl ether: hexane, using [^2^H_3_^13^C_2_^15^N]-lapatinib as an internal standard. Extracts were analyzed by LC/MS/MS using positive ion Electrospray (ESI+) TurboIonSpray® interface and multiple reaction monitoring. Sensitivity was less than 0.08ng/mL, and precision and accuracy within 15%. Mouse blood concentrations were converted to plasma concentrations for comparison, using a blood to plasma ratio of 0.7 (unpublished data).

### Quantitative Immunohistochemistry (qIHC)

Patient tumor tissue preparation and qIHC analysis was performed as previously described [14,15]. Tumor biopsies were fixed in 10% neutral buffered formalin and paraffin-embedded sections were prepared. Quantitative IHC analysis was performed in a Clinical Laboratory Improvement Amendments (CLIA) certified, College of American Pathologists (CAP) reference lab. The following antibodies were used: anti–HER2 (1:80; Vector Labs, Burlingame, CA), phosphorylated (p) EGFR (1:25), p-HER2 (1:1200), p-HER3 (1:125) (Cell Signaling Technology, Beverly, MA). Slides were analyzed by microscopy with the aid of a computer program that recognized areas of the biopsy containing tumor cells. Ten random fields from each slide were scored for the intensity of staining of the target and attributed an absorbance or optical density (OD) value. The mean OD of the 10 random fields provided an overall value for target expression in that biopsy [15]. The resulting OD measurements are proportional to concentration because the antibody interaction with its target is stoichiometric. However, this proportionality could not be directly quantified to allow determination of concentrations. Therefore, the OD data were assessed as ratios to obtain relative changes, and at the same time, avoid introducing additive error from the assay. Data were provided for total (phosphorylated + unphosphorylated) and the phosphorylated form alone.

### Data Analysis

Mouse concentration data on Day 3 of dosing, representing steady state in this species, were assessed for each tissue using calculated steady-state dosing interval AUC (linear trapezoidal method). Ratios of tumor/plasma and tumor/tissue were calculated from these AUC values. SAS JMP® v.11.0.0 was used to analyze the lapatinib concentration data in blood versus tumor tissue after multiple doses (five doses for mice dosed BID, and three doses for mice dosed QD). The concentration values were log transformed prior to performing the statistical analysis due to the heterogeneity of the variances among the groups. Concentrations in the dataset labeled BQL (Below the Quantitation Limit (20 ng/mL)) were set to 20 ng/mL, and the concentrations labeled ND (Not Determined) were set to 10 ng/mL for the statistical analysis. The differences between the blood and tumor were assessed via an Analysis of Variance (ANOVA) test. The variables Time and Tissue (tumor and blood), and the Time*Tissue interaction term were included in the model as fixed effects, and the Animal variable was included as a random effect nested within Tissue. Comparisons of lapatinib concentration in blood versus tumor at each time point were performed using the Student’s t-test. Data for the 100 mg/kg BID and the 200 mg/kg QD doses were analyzed separately. Comparisons with *p*-values ≤ 0.05 were considered statistically significant.

The primary outcome for the clinical data was relative biomarker expression in pre-treatment and post-treatment breast tumor tissue between the three dosing schedules. Secondary outcomes included adverse events and changes in lab values from pre-dose and post-dose. Additional measures not explicitly stated in the registered protocol included relative lapatinib steady-state trough concentrations in plasma and tumor and differences between the three dosing schedules. Although the data obtained were too limited to be fit to a concentration-response model, the results were compared to simulations using simple Emax models. Structural analysis shows that EGFR is in the inactive conformational state when bound to lapatinib [16]. It can be assumed that activated/phosphorylated EGFR, and presumably HER2 represent protein unavailable to bind to lapatinib, and the change in unphosphorylated protein in tumor that is available to bind to lapatinib can be calculated as the ratio of post- to pre-treatment values. Changes in p-HER3, which is not a target of lapatinib, were assessed as ratios of the post-treatment to pre-treatment values in each patient. These ratios represent the fractional change over time. This post-hoc analysis included patients with a plasma and a tumor concentration, and who over-expressed HER2 and/or EGFR.

## Results

### Pharmacokinetics in Mice

**Fig 2:**
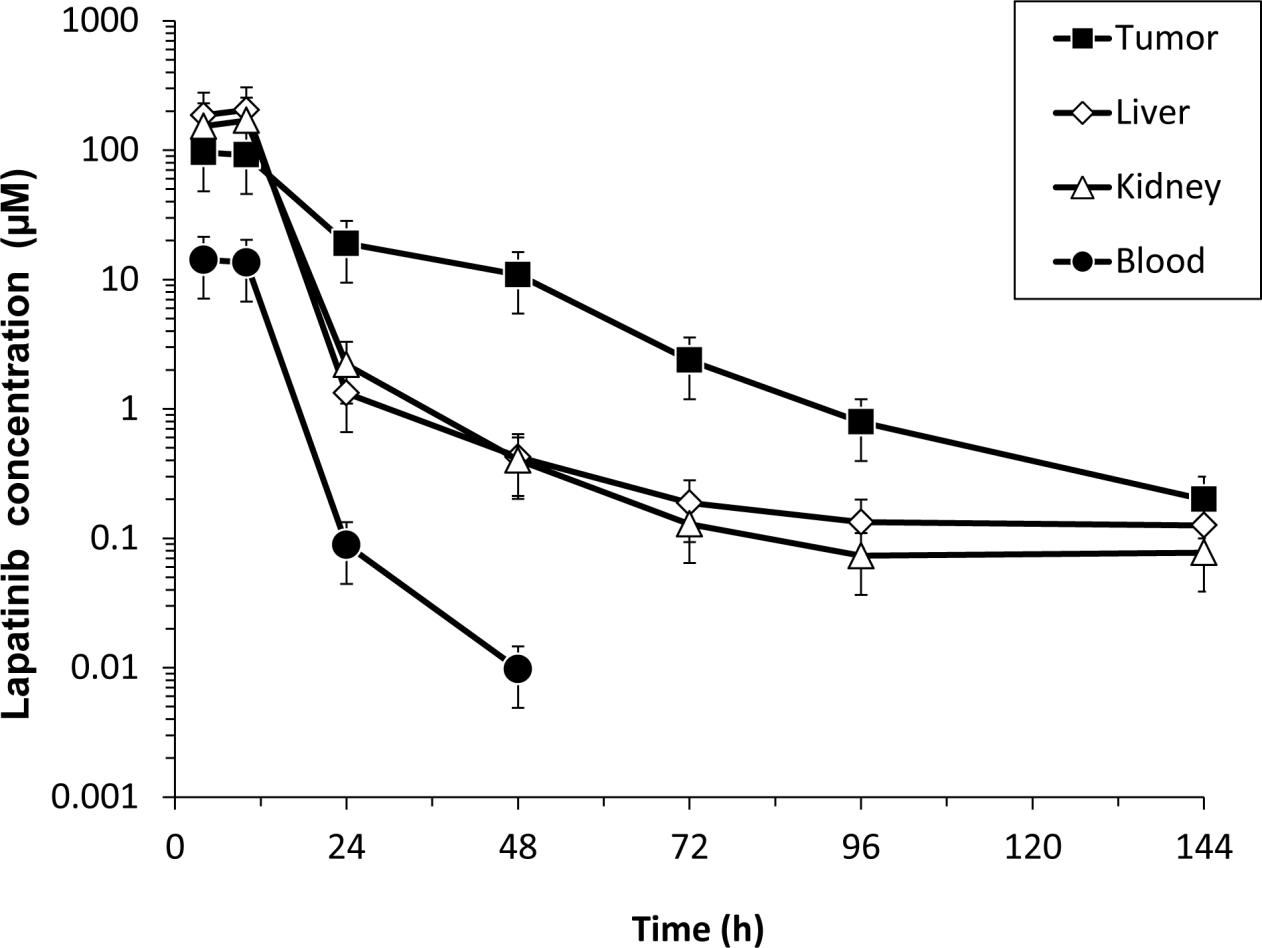
Lapatinib in human breast cancer xenograft tissue. Steady-state (Day 3) lapatinib concentrations in blood, tumor, kidney, and liver measured in CB-17 SCID female mice bearing established HER2+ BT474 breast cancer xenografts (n = 45) following treatment with lapatinib (100 mg/kg BID) administered by oral gavage x 5 doses. Results represent the mean +/- standard error.

We chose to examine lapatinib concentrations in tumor, plasma, kidney, and liver tissues from mice bearing BT474 HER2+ human breast cancer xenografts since this model reflects the approved clinical indication for lapatinib- HER2+ breast cancer- and the patient population studied in this trial. Lapatinib concentration versus time profiles in blood, tumor, liver, and kidney are shown for 100 mg/kg BID administered by oral gavage for 5 doses (Fig 2). A similar pattern was observed at the 200 mg QD dose (data not shown). Liver and kidney concentrations were generally superimposable. Together with tumor concentrations, these declined in parallel with blood at early time points (half-life 4 h). At later time points, liver, kidney, and tumor (at higher concentrations) displayed a slower parallel decline. At later time points, the slower parallel decline probably represents the presence of target-bound drug in these EGFR-expressing tissues [17].

Comparisons of mouse tumor and blood/plasma exposure were based on steady-state dosing interval AUC, because Cmax (peak concentration) sometimes occurred at later times in tumor than in blood. Each sample represented a single mouse; therefore, AUC was calculated using the mean of three concentrations. Exposure in tumor (AUC) was 4-fold higher (Table 1) with an apparent half-life that was 4-fold longer than blood (15.6 vs 3.9 h). Lapatinib concentrations in blood were statistically significantly lower in blood than in tumor, based on the ANOVA Time*Tissue interaction for both the 100 mg/kg and 200 mg/kg doses (*p*< 0.0001) (S1 Table). Statistically significant differences (*p*<0.05) between lapatinib concentrations in blood and tumor were seen at time points 4, 10, 24, 48, 72, 96, and 144 h post-dose for the 100 mg/kg dose (S2 Table). The differences in concentrations between the tissues for the 200 mg/kg dose were statistically significant at all post-dose time points. A visual presentation of the individual concentration data is provided in S1 Fig.

**Table 1:** Median (range) lapatinib exposure in mouse (n = 3) tumor, blood, and plasma after the fifth dose of 100 mg/kg BID (AUC 0–12) or third dose of 200 mg/kg QD (AUC 0–24).

AUC (μM×h)	100 mg/kg BID	200 mg/kg QD
Tumor	396 (325–521)	1953 (1066–2029)
Blood	104 (50–105)	739 (227–743)
Tumor / Blood Ratio	5.0 (3.8–6.5)	2.7 (2.6–4.7)
Blood / Plasma Ratio	0.7	0.7
Tumor / Plasma Ratio	7.1 (5.4–9.2)	3.9 (3.8–6.7)

### Pharmacokinetics in Patients

**Fig 3:**
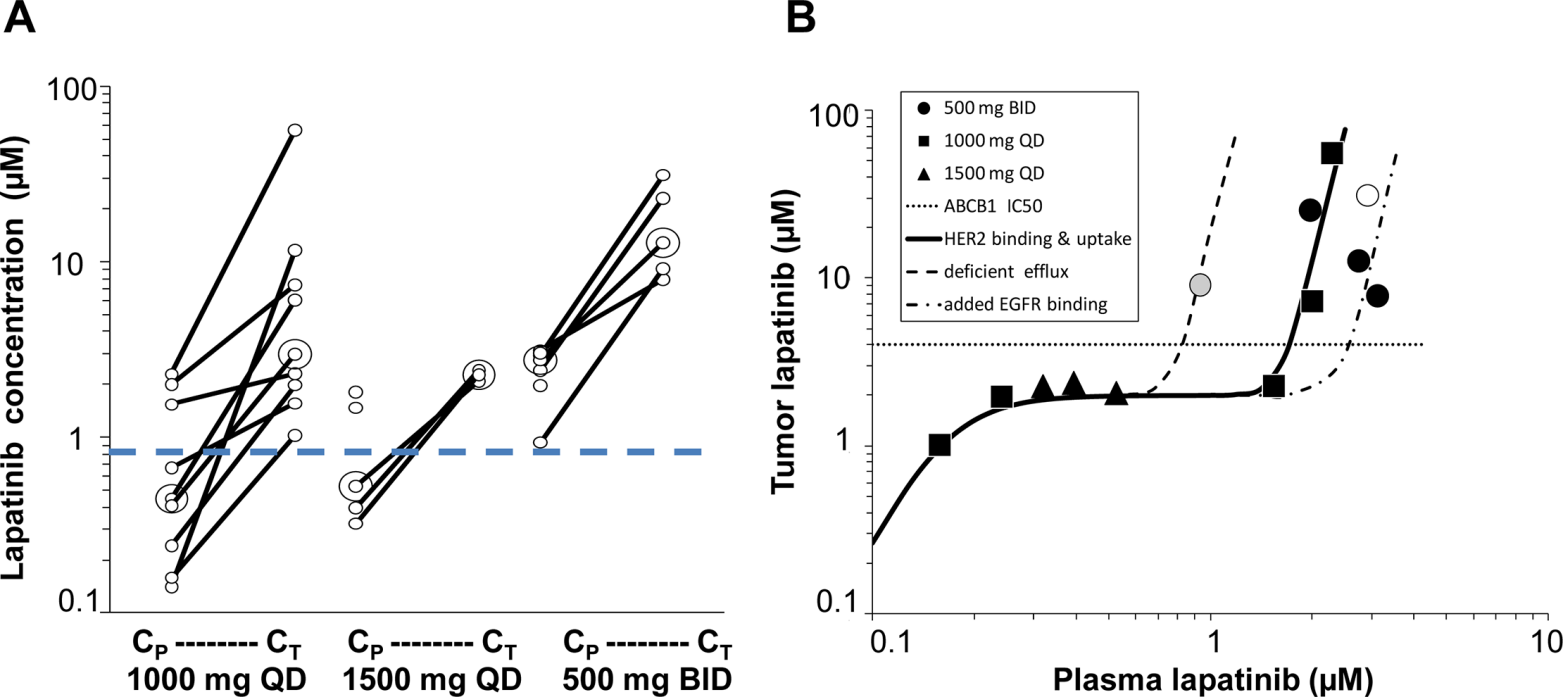
The relationship between lapatinib concentrations in tumor and plasma from clinical samples. Women with early stage HER2+ breast cancer were randomized to oral lapatinib monotherapy at 500 mg twice daily (BID), 1000 mg once daily (QD), or 1500 mg QD. Lapatinib concentrations (μM) in plasma (C_P_) and tumor (C_T_) tissue samples obtained simultaneously 9–15 days after initiating therapy (steady state) concentrations (C_min_). (**A**). Lapatinib concentrations for each patient in the indicated treatment groups. The dotted blue line represents the reported IC_90_ value for lapatinib in HER2+ breast cancer cell lines. Large circles represent group medians. (**B**) The complex relationship between lapatinib concentration in tumor and plasma showing a speculative model that suggests determinants: target protein binding (lower Emax curve), and net uptake/efflux (upper Emax curve): grey circle (patient with dysfunctional ABCB1 SNP C3435T); open circle (patient taking lapatinib 500 mg BID also taking grape seed oil), shown with simulation and deviations (dashed lines) illustrating speculation of diminished efflux (left shift) and increased EGFR target binding (right shift). The horizontal dotted line at 4 μM represents the IC_50_ for the ABCB1 transporter.

Twenty-eight patients were enrolled in the clinical study (Fig 1; Table 2). While the objective patient accrual involved 60 patients (20/treatment group), the study was interrupted for 21 months due to discovery and subsequent mitigation of a genotoxic impurity. The trial resumed for 14 additional months, but the initial goal for enrollment was not achieved due to slow accrual at the sites. Of the 28 patients enrolled in the clinical trial, 13 provided a plasma sample, a tumor tissue sample, and overexpressed and/or expressed HER2 and EGFR, respectively. A single blood sample was taken after 9–15 days of treatment, sufficient to achieve steady-state in plasma (24 h half-life). Sampling was performed one dosing interval after the last dose (Cmin) to obtain a stable estimate of steady state. In previous clinical studies, Cmin = 1.35 μM at 1500 mg QD [18]. Lapatinib concentrations were consistently higher in tumor than in plasma (Fig 3A and 3B; Table 3). Tumor concentration appeared to be related to plasma concentrations, but with a complex relationship that suggested multiple determinants. At plasma concentrations less than 2 μM, tumor concentrations rose to a plateau. This relationship paralleled target protein levels and likely represented binding to these proteins. In general, plasma lapatinib concentrations greater than 2 μM were associated with tumor concentrations greater than 4 μM, which is the IC_50_ for inhibition of the efflux transporter ABCB1, that lapatinib has been shown to inhibit [19]. These data are consistent with tumor concentration additionally determined by transporter-mediated cellular uptake, with a substrate-inhibited futile efflux. There was one exception to this pattern, a patient expressing a dysfunctional single nucleotide polymorphism (SNP) in ABCB1 (C3435T). A second patient with this same SNP showed no distinction in tumor concentration at lower plasma concentrations following treatment at 1500 mg QD. Tumor concentrations also appeared to segregate by dosing frequency. Patients dosed BID had higher tumor concentrations than patients dosed QD, which agrees with previous reports of higher plasma levels with the BID regimen [20]. Two patients receiving 1000 mg QD also achieved markedly higher levels of lapatinib in tumor, which could be due to their concurrent hyperbilirubinemia, suggesting poor biliary recycling of lapatinib and consequent increased bioavailability. Interestingly, the patient dosed BID with the highest tumor concentration was also taking grape seed oil, which contains pentagalloylglucose, an ABCB1 inhibitor [21]. These speculative observations require further study.

**Table 2:** Patient Demographics.^1^

Treatments	500 mg BID	1000 mg QD	1500 mg QD	All
Patients enrolled (no.)	10	11	7	28
Patients with samples (no.)	5	5	3	13
Reasons for withdrawals (no.)				
Adverse event	2	0	1	3
Lost to follow-up	0	1	0	1
Start of chemotherapy	1	2	0	3
Age (years), mean (range)	59 (45–91)	52 (35–60)	52 (40–60)	54 (35–91)
BMI (kg/m^2^), mean (range)	30 (22–38)	31 (22–45)	24 (20–30)	29 (20–45)
Ethnicity (no.)				
Black	0	1	1	2
Hispanic	2	2	0	4
White	8	8	6	22

1. Demographic data in this table are based on all enrolled patients

**Table 3:** Median (range) of lapatinib concentrations in tumor, plasma, and their ratios.

Dose regimen (n)	1500 mg QD (n = 3)	1000 mg QD (n = 5)	500 mg BID (n = 5)
**Tumor Cmin (μM)**	2.27 (2.08–2.39)	2.29 (1.02–56.0)	12.8 (7.9–31.1)
**Plasma Cmin (μM)**	0.39 (0.32–0.53)	1.54 (0.16–2.29)	2.75 (0.93–3.12)
**Tumor/plasma ratio**	6.1 (3.9–7.1)	6.5 (1.5–24.5)	9.7 (2.5–13)

Fig 3B shows a simulated relationship that describes target protein binding and net active uptake using two Emax models, one for target binding (first bracket) and the second for net tumor cellular uptake (second bracket):

$${\text{C}}_{\text{tumor}}=\left[{\text{B}}_{\text{max}}{•\text{C}}_{\text{p}}{}^{\lambda }/\left({\text{C}}_{\text{p}}{}^{\lambda }{+\text{BC}}_{50}{}^{\lambda }\right)\right]+\left[{\text{U}}_{\text{max}}{•\text{C}}_{\text{p}}{}^{\text{x}}/\left({\text{UC}}_{50}{}^{\text{x}}{+\text{C}}_{\text{p}}{}^{\text{x}}\right)\right]$$

This model resembled the data when the following values were used: B_max_ = 2, BC_50_ = 0.16 and λ = 4 in the first part relating to target protein binding, and U_max_ = 120, UC_50_ = 2.4 μM), and x = 12 in the second part relating to cellular uptake. The value of 2 for B_max_ reflects the upper limit of dimerization, and the value of 4 for λ reflects cooperativity between the HER receptor dimers and each of their lapatinib ligands. The values of UC_max_, UC_50_, and X, were arbitrary because no plateau was observed, and because they incorporate the futile cycle of any remaining efflux. In addition to resembling this unusual pattern in the data, this simulation (dashed lines in Fig 3B) also allowed an explanation for the deficient efflux associated with a SNP (left shift) and additional target binding in the presence of EGFR (right shift). Although speculative at this point, the correspondence between observations and the simulation (r^2^ = 0.915) suggests that this may be a plausible mechanism.

### Pharmacodynamics

**Fig 4:**
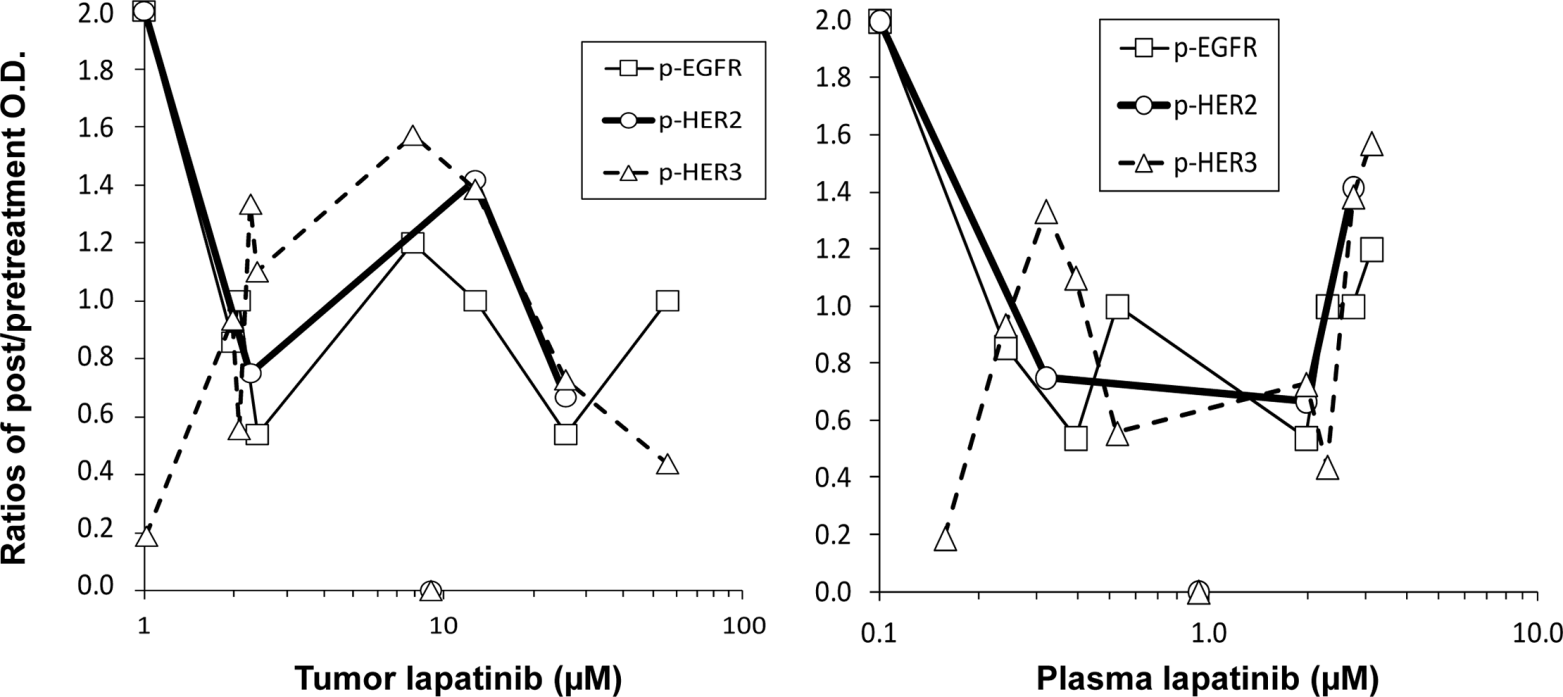
Phosphorylation of HER receptors relative to tumor and plasma concentrations of lapatinib. Changes (post-/pre-treatment ratios of optical density (OD values)) in EGFR, HER2, and HER3 phosphorylation in response to lapatinib in clinical tumor samples, and their relationship to tumor (left panel) and plasma (right panel) concentrations of lapatinib.

EGFR, HER2, and HER3 phosphorylation in the 13 patients who expressed at least one of these receptors was altered in the presence of lapatinib. Examples of IHC staining for total and phosphorylated forms of EGFR, HER2 and HER3 are shown in S2 Fig. Changes in receptor phosphorylation were represented by the ratio of post-/pre-treatment OD values relative to concentrations of lapatinib, as shown in Fig 4. Variability in ratios was generally lower than in individual OD values (S3 Table). As the lowest tumor concentration of lapatinib (1 μM) equivalent to the untreated state, phosphoryslation of HER2 and EGFR were relatively high compared to baseline, while p-HER3 was relatively low. As tumor concentrations of lapatinib increased to 2 μM, decreased p-EGFR and p-HER2 were apparent coincident with increasing p-HER3, presumably reflecting inhibition. Tyrosine phosphorylation of kinase deficient HER3 is dependent upon its dimerization partners e.g. HER2 and EGFR [22]. HER3 is not inhibited by lapatinib, so its persistent phosphorylation may play a role in preventing lapatinib from completely inhibiting HER2 and EGFR phosphorylation. Heterodimerization is consistent with the increased expression of phosphorylated HER2 and EGFR observed when tumor concentrations of lapatinib increased in the 2–10 μM range (Fig 4). Further increases in tumor concentration (>10 μM) were associated with decreased phosphorylation of HER3 and increased EGFR, consistent with a switch in HER3 heterodimers from HER3/HER2 to HER3/EGFR, or formation of EGFR homodimers.

The pattern in plasma differed according to the relationship with tumor concentration. Increasing plasma concentrations up to 2 μM had little effect on tumor concentrations, which exhibited a plateau. Increasing plasma concentrations above 2 μM had more dramatic effects in elevating phosphorylation, perhaps due to the complexity of mechanisms affecting these intracellular concentrations. No data was available above 3 μM in plasma at these doses.

Consistent with a role of inhibiting efflux in elevating tumor concentrations, there was one patient who achieved complete inhibition of HER2, EGFR, and HER3 phosphorylation at a plasma concentration of 1μM (9 μM in tumor). Interestingly, this patient also expressed a dysfunctional single nucleotide polymorphism (C3435T) in the efflux transporter ABCB1.

## Discussion

Small molecule tyrosine kinase inhibitors (TKIs) including lapatinib are designed to block the activity of their intended target and as a consequence, abrogate downstream signaling pathways that promote tumor growth and survival [23]. In the treatment of advanced stage solid tumors, TKIs are often administered in combination with cytotoxic chemotherapies or other targeted agents. The advantage of targeted agents is the wide index between efficacious and toxic doses, owing to target selectivity [24]. Determining a dose sufficient to maximally inhibit the target without additional toxicity (BED) is the goal, whether in combination or as monotherapy [8]. Dosing targeted agents on a continuous basis at an MTD may adversely impact compliance, and be counterproductive. The dose of TKIs required to achieve plasma drug concentrations ≥ IC_50_ or IC_90_ based on *in vitro* studies is frequently used as an initial estimate of a BED. This strategy is often based on the assumption that plasma concentrations of drug are reflected by tumor concentrations; however our findings suggest this does not apply to lapatinib. To the best of our knowledge, this is the first published study that not only shows the relationship between concurrent lapatinib concentrations in plasma and tumor, but also explores the correlations between plasma/tumor drug concentrations and the phosphorylation state of lapatinib targets (HER2;EGFR), and their key co-receptor HER3.

The low aqueous solubility of lapatinib predisposes extensive (>99%) binding to plasma albumin, which delivers lapatinib to systemic tissues. Albumin binding of lapatinib is linear across the range in this study. Plasma concentrations of albumin in this study ranged from 568 to 712 μM, more than 80-fold higher than lapatinib concentration in plasma. Lapatinib can also bind in plasma to α_1_- acid glycoprotein (AAG); however, in contrast to albumin, the contribution of this low capacity, acute-phase reactant protein is likely minimal. Albumin binding of lapatinib does not limit uptake in tissues that express its more avidly bound targets, e.g. liver, kidney, and tumor. Previous studies have shown that lapatinib is highly selective for its intended targets, HER2 and EGFR, at concentrations as high as 10–26 μM [9,10]. Steady-state equilibrium in the tumor can therefore be described as the net result of competing binding affinities and uptake/efflux transporters. Target binding in tumor dominates that in plasma, with greater affinities (IC_50_ of 0.025 μM for HER2, and 0.029 μM for EGFR) that effectively extract albumin-bound lapatinib from blood passing through the tumor. At the same time, active uptake into tumor cells may also have an impact on tumor concentrations. Concentrations in tumor tissue were 6-fold higher with QD dosing and 10-fold higher with BID dosing compared with concentrations of lapatinib in plasma. Although lapatinib is currently administered on a QD schedule, these data showed that 500 mg BID dosing resulted in increased tumor as well as plasma concentrations of lapatinib compared with two QD regimens of equal or greater total daily dose. These findings are consistent with a previous phase II clinical trial comparing several BID and QD doses. In that study, BID dosing resulted in higher plasma concentrations than equivalent cumulative QD doses [20]. In addition, a phase II clinical trial comparing 500 mg BID versus 1500 mg QD single agent lapatinib showed a tendency towards increased clinical efficacy in the BID dosing cohort, although the study was not sufficiently powered to show a statistical difference between the two groups [25]. With similar toxicity profiles, and existing data indicating higher plasma and now tumor concentrations, it is tempting to speculate that BID dosing may be a more clinical effective schedule.

In the treatment of advanced stage HER2+ breast cancer, the development of therapeutic resistance remains a significant dilemma limiting the clinical efficacy of lapatinib. Although resistance appears to be a multi-factorial process, one potential mechanism is related to persistent HER3 activation (phosphorylation), which we observed in our study at certain tumor concentrations of lapatinib. The downstream growth and survival effects of HER receptors are mediated by HER homo- or heterodimers, with the latter the more potent signaling complex. Dimerization patterns are influenced by the expression level of HER receptors and their affinity for one another. In addition, binding of soluble ligands (e.g. heregulin, EGF) to their cognate HER receptors impacts dimerization patterns [26,27]. The hierarchy of dimer composition based on binding affinities favors formation of HER2/HER3 heterodimers, followed by HER2/EGFR, EGFR/HER3, and EGFR/EGFR. Heterodimerization serves an important purpose in the case of HER3, which possesses low level autokinase activity requiring transactivation through its partner [22]. Of the HER family members, HER3 contains the most PI3K binding sites [28]. With HER2 serving to amplify the signal, HER2/HER3 heterodimers potently activate the PI3K-Akt-mTOR signaling pathway that promotes tumor cell survival, therapeutic resistance, and metastatic dissemination [29].

There are questions as to whether the current dose of lapatinib adequately inhibits HER2/EGFR signaling. Although the data in Fig 4 are limited, they are consistent with incomplete inhibition of p-HER2 and p-EGFR at lapatinib concentrations in tumor of 1–10 μM. The persistent phosphorylation of HER2 and EGFR at these lower drug concentrations in tumor may be mediated through increased expression of p-HER3 leading to the formation of heterodimers that are resistant to inhibition by lapatinib [30]. It appears that more effective inhibition of HER3-containing heterodimers may be achieved at higher tumor concentrations of lapatinib (> 10 μM). Inhibition of EGFR phosphorylation by lapatinib is less efficient and requires increased concentrations compared with HER2 (Fig 4). These findings are consistent with our recent observations that lapatinib does not effectively block EGFR phosphorylation, particularly at certain phosphotyrosine sites [30]. Increasing tumor concentrations of lapatinib from 1–10 μM appeared to be associated with increased HER3 phosphorylation, and persistent phosphorylation of EGFR. These observations are similar to our recent findings in which therapeutic resistance to lapatinib in preclinical models was associated with a switch in the regulation of breast cancer cell survival from HER2-HER3-PI3K in the treatment naïve state, to a HER3-EGFR-PI3K signaling axis that is resistant to inhibition by lapatinib [30]. Another mechanism of resistance relates to successful inhibition of HER2 signaling by lapatinib, which leads to inhibition of downstream PI3K-Akt signaling and de-repression of the transcription factor FOXO3a. As a consequence, increased transcriptional activity of FOXO3a upregulates genes that have been shown to contribute to lapatinib resistance, including the estrogen receptor and HER3 [31,32]. Thus, it may be advantageous to maintain tumor concentrations of lapatinib in a range that does not trigger a switch to HER3-EGFR heterodimerization. If subsequent therapeutic resistance or suboptimal response to lapatinib occurs at that concentration, it may introduce an opportunity to then increase dosages, or switch to (or combine with) an alternative inhibitor (e.g. afatinib or neratinib, irreversible inhibitors of EGFR and HER2) which have been shown to block HER3 transphosphorylation [30,33].

Treatment with intermittent high doses of lapatinib in patients has been proposed as a more effective strategy to completely block HER2 signaling and prevent this form of resistance, while mitigating the toxicities of continuous dosing [34]. In this context, an intermittent schedule of high doses of lapatinib was shown by Moasser and colleagues to be effective at blocking HER2/HER3 signaling and consequently triggering apoptosis in HER2+ tumor cell lines and breast cancer xenografts [34]. In addition, an increased antitumor response was observed in tumor xenograft studies when mice were treated with lapatinib at 400 mg/kg BID for 5 days in three 14 day cycles compared with continuous dosing at 100 mg/kg BID for 42 consecutive days [35]. The intermittent high dose of lapatinib resulted in peak plasma concentrations of approximately 8000 ng/mL (14 μM) compared with 800 ng/mL (1.4 μM) at the lower continuous dose. However, in our mouse study, 100 mg/kg BID x 5 doses resulted in peak plasma concentrations of 18μM and tumor concentrations of 135 μM. In comparison, the median tumor concentration of lapatinib in patients treated with 500 mg BID in EGF10027 was 13 μM (Table 3). Moasser and colleagues focused on elevating plasma concentrations as a means to raise tumor concentrations. Our data suggests that this strategy will result in tumor concentrations that greatly exceed the reported levels necessary for target inhibition. Twice daily dosing might produce an improved response, as could co-administration of an ABCB1 inhibitor that can achieve intra-tumor concentrations of lapatinib required to maximally inhibit HER2 and EGFR signaling. In the clinical trial of high doses of lapatinib conducted by Moasser and colleagues, it may be that the responses seen with lapatinib and ketoconazole were due to the ability of ketoconazole to inhibit ABCB1 in tumor, and not just CYP3A4 in the intestine [35]. Additional studies are needed to assess if ABCB1 inhibition could serve as a tool to elevate lapatinib levels in a stereotactic manner to decrease toxicity and increase efficacy.

When increasing the dose of any drug it is important to be aware of toxicity, which includes diarrhea for lapatinib. This is presumably a locally induced mechanism-based toxicity, as enterocytes are very dependent upon EGFR for their high turnover rate [36,37]. Exposure to lower doses given BID may mitigate this toxicity. Interestingly, with respect to liver toxicity, peak concentrations of lapatinib were higher in mouse liver than in mouse blood (Fig 1). EGFR also plays an important role in liver function and regeneration from injury [38]. The flattened profiles of a BID regimen (i.e. lower peaks, higher troughs) may also help mitigate this potential side effect. Furthermore, dosing to a BED rather than an MTD allows titrating to an optimal dose that maximizes therapeutic efficacy while reducing toxicity in normal tissue.

Our preclinical and clinical investigations of lapatinib concentrations in plasma and tumor underscore a potential need to refine dose selection. Although based on a small sample size, the limited data in this study revealed a discrepancy between plasma concentrations of lapatinib and those in the tumor, the drug's target site. Not only do plasma levels of lapatinib underestimate corresponding concentrations in tumor and normal EGFR expressing tissues, but multiple determinants of tumor concentration complicate this relationship making predictions difficult. The data suggest, but could not identify, an “optimal” concentration that can achieve effective target inhibition. Using plasma levels of lapatinib as a surrogate to determine a BED based on an IC_90_ as a benchmark can mislead our understanding of what is truly occurring within the tumor. Such misinformation may lead to dose escalation resulting in tumor concentrations that can activate redundant survival pathways that contribute to therapeutic resistance. Importantly, pushing tumor concentrations beyond the optimal concentration may disrupt the balance of HER receptor dimers, favoring a HER3-EGFR-PI3K signaling axis, which is not effectively blocked by lapatinib. Though recognizing that many of the speculations in this study are based on a small data set, it is hoped that several opportunities for ongoing improvement in cancer treatment pertaining to targeted therapies have been pointed out. Emphasizing the inherent risks in exceeding a biologically effective dose, these findings suggest that a greater understanding of the relationship between plasma and tumor drug concentrations, and effects at the intended target(s) is required in order to optimize treatment of small molecule kinase inhibitors. Doing so will likely enhance the clinical efficacy of targeted therapies while reducing potential toxicity.

## Conclusions

The relationship between plasma and tumor concentrations of lapatinib is complicated by more than one determinant that makes it difficult to predict, but needs to be addressed in the course of rational dose selection. These analyses should be viewed with caution because they are based on limited data. Although their consistency with previously reported observations lends some validity, additional data are required to support these conclusions.

## Supporting Information

S1 CONSORT Checklist CONSORT 2010 checklist. (DOC)

S1 Fig Individual Animal Log Lapatinib Concentration Variability Plot. Individual animal log lapatinib concentration values (Day 3) in blood and tumor tissues over post dose time points 1, 4, 10, 24, 48, 72, 96, and 144 hours for the 100 mg/kg BID and 200 mg/kg QD doses.
(TIF)

S2 Fig Examples of total and phosphorylated EGFR, HER2, and HER3 by IHC. Immunohistochemistry staining of (A) total EGFR expression; (B) pre- and paired (C) post-treatment EGFR phosphotyrosine. IHC of (D) total HER2 expression; (E) pre- and paired (F) post-treatment HER2 phosphotyrosine staining. And, (G) total HER3; (H) pre- and paired (I) post-treatment HER3 phosphotyrosine.
(TIF)

S1 Protocol EGF10027 protocol. (PDF)

S1 Table Pharmacokinetic parameter estimates of lapatinib in animal tissue. Lapatinib was administered to fed female tumor-bearing CB-17 SCID mice orally to ascertain the pharmacokinetics and pharmacodynamics of its free base. Blood, kidney, liver and BT474 tumor samples were collected up to 144 hours after a single dose or multiple doses (100, and 200 mg/kg BID or QD for 3 days). Plasma and tissue samples were analyzed for lapatinib concentration by LC/MS/MS. Values shown are based on the calculated mean. Mean values for Tmax (time to maximum concentration) and MRTlast (mean resonance time) are indicated in hours, while Cmax (maximum concentration) and AUC (area under the curve) are indicated in ng/ml.
(PDF)

S2 Table Concentration-time profiles of lapatinib (ng/mL) in animal tissue. Data were from female mice after single and repeat oral administration at 100 mg/kg BID, or 200 mg/kg QD.
(PDF)

S3 Table Optical density (OD) of phosphorylated EGFR, HER2 and HER3, and ratios of post to pre-treatment OD values in patient tumor samples. (PDF)
