# Supplementary figures and images for: Lapatinib Plasma and Tumor Concentrations and Effects on HER Receptor Phosphorylation in Tumor
Source: PLoS One. 2015 Nov 16;10(11):e0142845. doi: 10.1371/journal.pone.0142845 (PMC4646457; doi:10.1371/journal.pone.0142845)

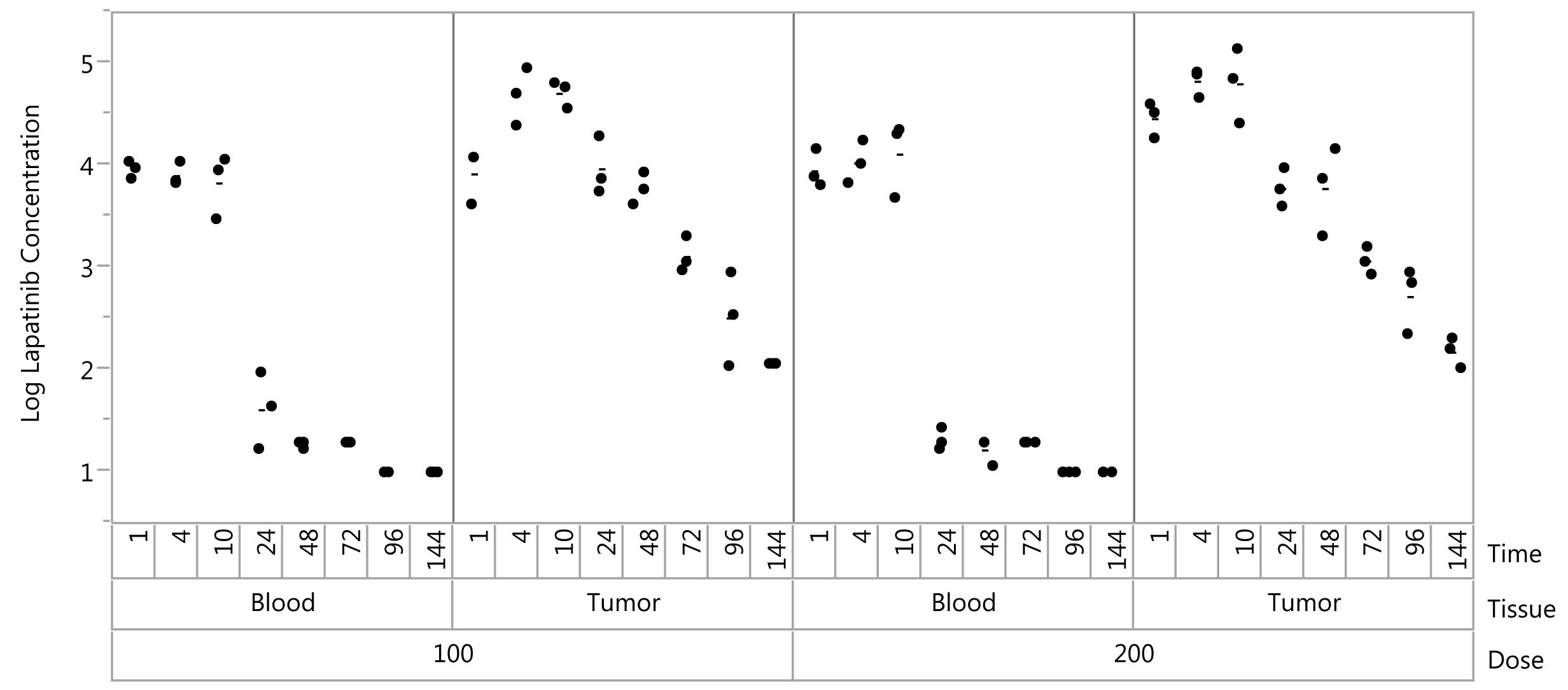

Supplement: S1 Fig — Individual animal log lapatinib concentration values (Day 3) in blood and tumor tissues over post dose time points 1, 4, 10, 24, 48, 72, 96, and 144 hours for the 100 mg/kg BID and 200 mg/kg QD doses. (TIF) [file pone.0142845.s002.tif]

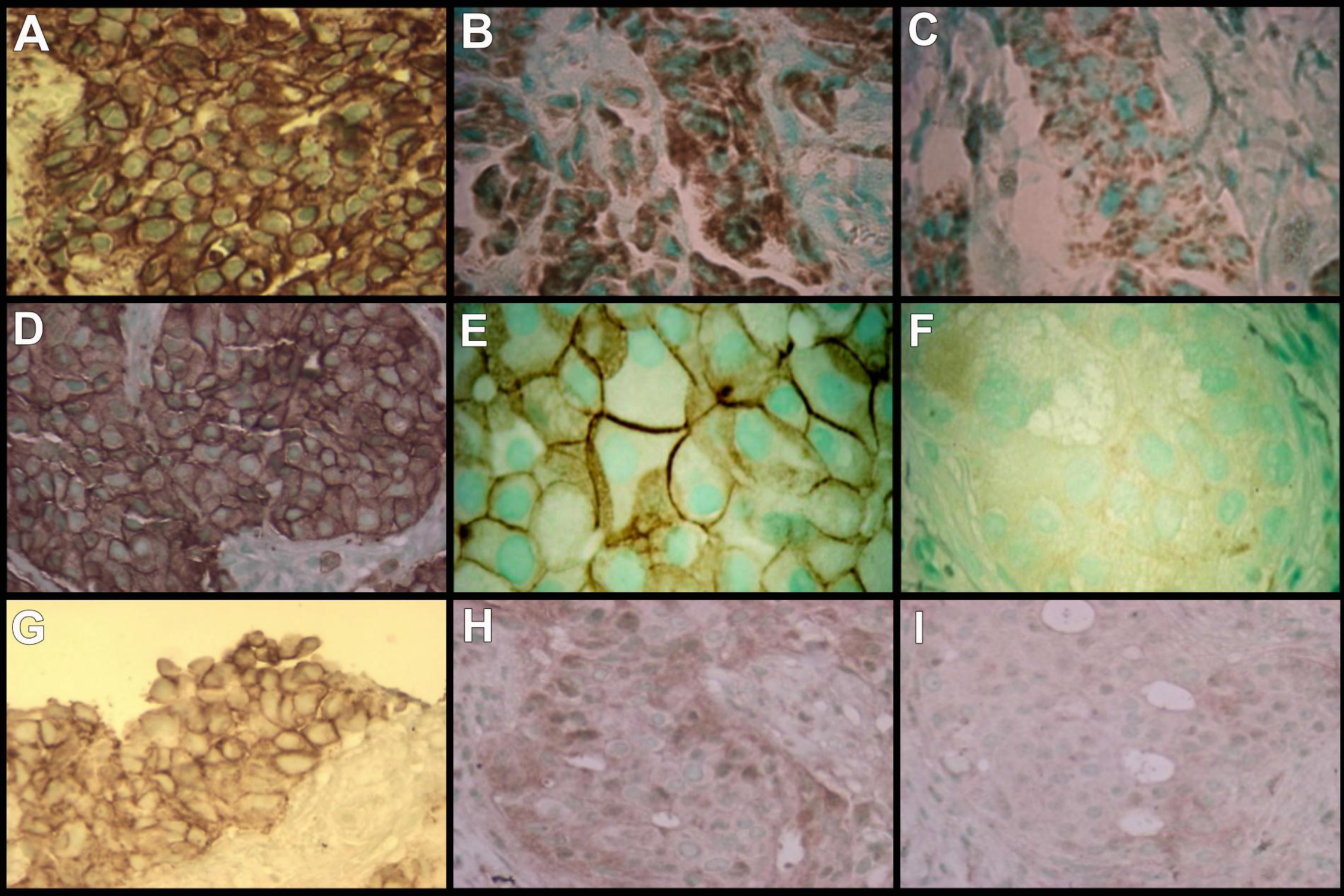

Supplement: S2 Fig — Immunohistochemistry staining of (A) total EGFR expression; (B) pre- and paired (C) post-treatment EGFR phosphotyrosine. IHC of (D) total HER2 expression; (E) pre- and paired (F) post-treatment HER2 phosphotyrosine staining. And, (G) total HER3; (H) pre- and paired (I) post-treatment HER3 phosphotyrosine. (TIF) [file pone.0142845.s003.tif]
