# Supplementary material for: Lapatinib Plasma and Tumor Concentrations and Effects on HER Receptor Phosphorylation in Tumor
Source: PLoS One. 2015 Nov 16;10(11):e0142845. doi: 10.1371/journal.pone.0142845 (PMC4646457; doi:10.1371/journal.pone.0142845)
Supplement: S1 Protocol — (PDF) [file pone.0142845.s004.pdf]

Division: Worldwide Development
Retention Category: GRS019
Information Type: Protocol Amendment

**Title:**	A Phase I, Open Label Study of the Safety, Pharmacokinetics and Pharmacodynamics of Lapatinib (GW572016) in Patients with Treatment-Naïve, ErbB2 Positive Breast Cancer

Compound Number: GW572016

Effective Date: 08-OCT-2007

Protocol Amendment Number: 03

Description:

Over-expression of growth factor receptors ErbB1 and ErbB2 has been associated with a poor clinical outcome in a variety of epithelial malignancies. Inhibition of these receptors has been shown to disrupt signaling pathways regulating cell proliferation, survival and differentiation. Lapatinib (GW572016) is a reversible dual inhibitor of ErbB2 and EGFR and has resulted in tumor growth arrest and/or apoptosis in ErbB1 and ErbB2 dependent tumor cell lines and xenografts. Safety data from the first human trials revealed no clinically significant changes in safety laboratory assessments or ECG parameters pre- to post-treatment. The most commonly reported adverse events were diarrhea, rash, nausea, headache, flatus and fatigue in healthy volunteers and patients treated with once daily dosing regimens. This study will examine the ability of lapatinib to inhibit downstream mediators of tumor cell growth and survival (e.g. AKT and ERK1/2). Patients with treatment-naïve, breast tumors that overexpress ErbB2 protein or demonstrate ErbB2 gene amplification will be enrolled. In addition, safety and pharmacokinetic data will be collected.

Subject: lapatinib (GW572016), ErbB2, ErbB1 (EGFR), pharmacokinetics, pharmacodynamics, safety, breast cancer

Author: Oncology MDC: Maria Koehler, M.D., Ph.D; Catherine Ellis; CPDM: Melissa Versola; Kevin Koch; Marla Bowman; Discovery Biometrics: Nikita Arya

Copyright 2007 the GlaxoSmithKline group of companies. All rights reserved. Unauthorised copying or use of this information is prohibited.

Revision Chronology:

RM2002/00456/00	2003-JUL-30	Original
RM2002/00456/01	2003-NOV-20	Amendment 01:

1. Patient population restricted to breast cancer patients who will undergo surgical resection as their primary definitive therapy.

2. Study changed to reflect a 3-arm, randomized design to include GW572016 doses 1500 mg QD, 1000mg QD and 500 mg BID. The low-dose (250mg), non-randomized arm was eliminated.

3. Study treatment duration was shortened to 9 to 13 days (previously 14 to 21 days) and resection to occur on Day 10 to 14 (previously Day 14 to 21)

4. Collection of normal breast tissue adjacent to the tumor was eliminated.

5. The twenty-four hour pharmacokinetic sampling on Day 1 and at steady state (Day 12 to 19) was eliminated. A single blood sample for pharmacokinetic analysis will be collected pre-dose (Day 0 or 1) and at the time of resection (Day 10 to 14).

6. Transcriptome analysis of tumor tissue using microarray was eliminated.

7. Analysis of tumor tissue for intratumoral GW572016 concentrations was added.

8. An updated list of prohibited medications has been added.

RM2002/00456/02	2005-JAN-25	Amendment 02:<br><br>1. The number of participating study centers was increased to 10.<br><br>2. The target patient population was amended to include patients with breast cancer lesions 1 cm and greater.<br><br>3. The protocol-defined list of acceptable contraceptive choices was updated.<br><br>4. The toxicity criteria was updated to NCI CTCAE version 3.0.<br><br>5. Criteria for defining ErbB2 overexpression and ErbB1 (EGFR) expression was added.<br><br>6. Criteria for evaluating compliance to study medication was added.<br><br>7. The window for obtaining the pre-treatment biopsy was expanded from 3 days to 14 days.<br><br>8. Oral steroids and alcohol use were removed as exclusion criteria.<br><br>9. The window for completing the Post Study Visit was expanded from 28 days to 7-28 days.<br><br>10. The prohibited medication list was updated.<br><br>11. Urinalysis was deleted as a protocol-defined laboratory test.<br><br>12. The number of tablets of GW572016 (lapatinib) was changed from 100 tablets to 90 tablets per bottle.<br><br>13. Text was added indicating PGx samples may be shipped amibient (real time) or frozen (if batched).

		14. Text was added indicating that the medical monitor must be notified if surgical resection cannot be performed within the protocol defined timelines.<br><br>15. Text clarifying that if archived tissue was used to determine elgibility, an additional biopsy must be obtained within 14 days of initiating dosing.<br><br>16. Global replacement of lapatinib for GW572016 and ErbB1 for EGFR was made throughout the protocol document.
RM2002/00456/03	2007-OCT-08	Amendment 03:<br><br>1. Study design changed to single arm lapatinib administered daily at 1500mg dose. Removed the 1000mg daily and 500mg BID doses.<br><br>2. Eligibility requirement to include ErbB2 positive tumors only.<br><br>3. Archived tumor tissue is allowed for the pre-treatment tissue; removed language requiring biopsy before starting study treatment.<br><br>4. Updated the protocol to include data from EGF100151 which supported FDA approval of TYKERB™.<br><br>5. Biomarker studies may include protein, DNA and/or RNA analyses, added language to clarify this approach.<br><br>6. Updated safety information and prohibited medications.

7. Added language to define primary and secondary cardiac endpoints; appendix for referencing NYHA Class III and IV cardiac function classifications.

CONFIDENTIAL RM2002/00456/03

**Sponsor Signatory:**	**Signature:**	**Date:**
Barbara Weber, M.D.<br>VP Translational Medicine<br>Clinical Pharmacology &<br>Discovery Medicine	Barbara L. Weber	8 Oct 2007

SPONSOR INFORMATION PAGE

Title:	A Phase I, Open Label Study of the Safety, Pharmacokinetics and Pharmacodynamics of Lapatinib (GW572016) in Patients with Treatment-Naïve, ErbB2 Positive Breast Cancer
Study Identifier:	EGF10027

GlaxoSmithKline
Five Moore Drive
P.O. 13398
Research Triangle Park, NC 27709-3398, USA
Telephone: + 001-919-483-2100

Primary Medical Monitor:

Maria Koehler

Secondary Medical Monitor:

Barbara Weber

Tertiary Medical Monitor:

Peter Lebowitz

INVESTIGATOR AGREEMENT PAGE

I confirm agreement to conduct the study in compliance with the protocol, as amended by this protocol amendment.

Investigator Name: ______________________________

____ ______________________

Investigator Signature Date

TABLE OF CONTENTS

	Page
ABBREVIATIONS	13
PROTOCOL SUMMARY	15
1. INTRODUCTION	20
1.1. Background	20
1.2. Preclinical Results	20
1.3. Toxicology	21
1.4. Preliminary Results from Healthy Volunteers and Patients	22
1.5. Rationale	23
2. OBJECTIVE(S)	24
2.1. Primary	24
2.2. Secondary	24
3. ENDPOINT(S)	25
3.1. Primary	25
3.2. Secondary	25
4. STUDY DESIGN	25
5. STUDY POPULATION	26
5.1. Number of Subjects	26
5.2. Eligibility Criteria	26
5.2.1. Inclusion Criteria	26
5.2.2. Exclusion Criteria	27
5.2.3. Other Eligibility Criteria Considerations	28
6. STUDY ASSESSMENTS AND PROCEDURES	28
6.1. Demographic and Baseline Assessments	28
6.2. Screening Visit	29
6.3. On Study Assessments	29
6.4. Post Study Visit	30
6.5. Safety	30
6.5.1. Pregnancy	30
6.5.2. Clinical Laboratory Tests	31
6.5.3. Cardiac Assessments	31
6.5.4. Adverse Events	32
6.5.5. Criteria for Study Hold	32
6.6. Pharmacokinetics	32

6.7. Pharmacodynamics 33
6.7.1. Tumor Tissue Samples 33
6.8. Pharmacogenetics 34
6.8.1. Background 34
6.8.2. Scope of PGx Analysis 35
6.8.3. Sample Quality Control (QC) 35
6.8.4. Pharmacogenetics Objectives 35
6.8.5. Pharmacogenetics Study Population 35
6.8.6. Pharmacogenetic Samples 36
6.8.7. Subject Withdrawal from Pharmacogenetic Research 37
6.8.8. Screen and Baseline Failures for Pharmacogenetic Samples . 37
6.8.9. Pharmacogenetic Analyses 37

7. INVESTIGATIONAL PRODUCT(S) 39
7.1. Description of Investigational Product 39
7.2. Dosage and Administration 39
7.3. Dose Rationale 39
7.4. Treatment Assignment 40
7.5. Packaging and Labeling 40
7.6. Handling and Storage 40
7.7. Product Accountability 41
7.8. Assessment of Compliance 41
7.9. Treatment of Investigational Product Overdose 41
7.10. Occupational Safety 41

8. CONCOMITANT MEDICATIONS AND NON-DRUG THERAPIES 42
8.1. Permitted Medications 42
8.2. Prohibited Medications 42

9. SUBJECT COMPLETION AND WITHDRAWAL 43
9.1. Subject Completion 43
9.2. Subject Withdrawal 44

10. ADVERSE EVENTS (AE) AND SERIOUS ADVERSE EVENTS (SAE) . . 44
10.1. Definition of an AE 44
10.2. Definition of a SAE 45
10.3. Lack of Efficacy 46
10.4. Clinical Laboratory Abnormalities and Other Abnormal Assessments as AEs and SAEs 46
10.5. Time Period, Frequency, and Method of Detecting AEs and SAEs . 47
10.6. Recording of AEs and SAEs 47

10.7. Evaluating AEs and SAEs 48
10.7.1. Assessment of Intensity 48
10.7.2. Assessment of Causality 48
10.8. Follow-Up of AEs and SAEs 49
10.9. Prompt Reporting of SAEs to GSK 49
10.9.1. Timeframes for Submitting SAE Reports to GSK 49
10.9.2. Completion and Transmission of the SAE Reports 49
10.10. Regulatory Reporting Requirements for SAEs 50
10.11. Post-study AEs and SAEs 51
10.12. SAEs Related to Study Participation 51

11. DATA ANALYSIS AND STATISTICAL CONSIDERATIONS 51
11.1. Hypotheses 51
11.2. Sample Size Considerations 51
11.2.1. Sample Size Assumptions 51
11.3. Analysis Populations 52
11.4. General Considerations for Data Analysis 52
11.4.1. Withdrawal 52
11.4.2. Missing Data 52
11.4.3. Assessment Windows 52
11.4.4. Other issues 52
11.5. Safety Analyses 52
11.5.1. Extent of Exposure 53
11.5.2. Adverse Events 53
11.5.3. Clinical Laboratory Evaluations 53
11.5.4. Other Safety Measures 53
11.6. Clinical Pharmacology Data Analyses 53
11.6.1. Pharmacokinetic Analyses 53
11.6.2. Pharmacodynamic Analyses 53

12. STUDY ADMINISTRATION 54
12.1. Regulatory and Ethical Considerations 54
12.1.1. Regulatory Authority Approval 54
12.1.2. Ethical Conduct of the Study and Ethics Approval 54
12.1.3. Informed Consent 55
12.1.4. Investigator Reporting Requirements 55
12.2. Study Monitoring 55
12.3. Quality Assurance 56
12.4. Study and Site Closure 56

12.5. Records Retention 57
12.6. Provision of Study Results and Information to Investigators 57
12.7. Information Disclosure and Inventions 57
12.8. Data Management 59
12.9. Confidentiality of a Subject's Pharmacogenetic Data 59

13. REFERENCES 60

14. APPENDICES 62
14.1. Appendix 1: Time and Events Table 62
14.2. Appendix 2: Clinical Laboratory Assessments 63
14.3. Appendix 3: Country Specific Requirements 64
14.4. Appendix 4: NCI Performance Status Scale/Score, Karnofsky Performance Status Scale/Score 65
14.5. Appendix 5: New York Heart Association Functional Classification 66
14.6. Appendix 6: Amendment 01 Summary of Changes 67
14.7. Appendix 7: Amendment 02 Summary of Changes 98
14.8. Appendix 8: Amendment 03 Summary of Changes 111

ABBREVIATIONS

AE	Adverse event
ALT	Alanine aminotransferase
AST	Aspartate aminotransferase
AUC_τ	Area under the plasma drug concentration versus time curve within the dosing interval
BID	Twice daily
β-hCG	Human chorionic gonadotropin
°C	Degrees centigrade
C_{max}	Maximum observed concentration
CBC	Complete blood count
CIB	Clinical Investigators' Brochure
CRF	Case report form
CTCAE	Common Terminology Criteria for Adverse Events
CYP	Cytochrome P450
DB	Discovery Bioinformatics
ECG	Electrocardiogram
ECD	Extracellular domain
ECHO	Echocardiogram
EDTA	Ethylenediamine tetraacetic acid
EISR	Expedited investigator safety report
Erb	Erythroblastosis
EGFR	Epidermal growth factor; ErbB-1; or tyrosine kinase-type cell surface receptor HER1
FDA	Food and Drug Administration
FISH	Fluorescence in situ hybridization
GCP	Good Clinical Practice
GCSP	Global Clinical Safety and Pharmacovigilance
GSK	GlaxoSmithKline
HCT	Hematocrit
IC_{50}	Concentration of compound producing half-maximal inhibition
IEC	Independent Ethics Committee
IHC	Immunohistochemistry
IND	Investigational New Drug Application
IRB	Institutional Review Board
kg	Kilogram(s)
KPS	Karnofsky Performance Status
L	Liter
LVEF	Left ventricular ejection fraction
M	Moles per liter
m^2	Square meters
MCH	Mean cell hemoglobin
MedDRA	Medical Dictionary for Regulatory Activities
mins	Minutes
MCV	Mean cell volume
mg	Milligram(s)

MTD	Maximum tolerated dose
mL	Milliliter(s)
MUGA	Multigated angiogram
NCI	National Cancer Institute
ng	Nanogram
nmol	Nanomole
μM	Micromolar
NOAEL	No-observed adverse effect level
PGx	Pharmacogenetics
PI	Principal Investigator
PK	Pharmacokinetic
p-tyr	Phosphotyrosine
QD	Once daily
RAP	Reporting and Analysis Plan
RBC	Red blood cell(s)
SAE	Serious adverse event(s)
SNP	Single nucleotide polymorphisms
SGOT	Serum glutamic oxaloacetic transaminate (AST)
SGPT	Serum glutamic pyruvic transaminase (ALT)
t_{max}	Time to maximum observed plasma drug concentration
UK	United Kingdom
US	United States
WBC	White blood cell(s)

Trademark Information

Trademarks of the GlaxoSmithKline group of companies
TUMS
TYKERB

Trademarks not owned by the GlaxoSmithKline group of companies
Herceptin
Iressa
Maalox
Mylanta
Rennies
Tarceva

PROTOCOL SUMMARY

Rationale

Breast cancer is the most common malignancy in women in the United States. Despite a variety of hormonal, cytotoxic and biologic approaches, a significant number of tumors are resistant to currently approved treatment modalities [Ring, 2002]. There is evidence that epidermal growth factor receptor (EGFR, ErbB1) and ErbB2 overexpression in breast cancers are independently associated with more aggressive tumor proliferation and a poor prognosis [Klijn, 1992; Kaptain, 2001]. ErbB1 and ErbB2 are members of the Type I family of receptor tyrosine kinases (ErbB). Tyrosine autophosphorylation of ErbB1/ErbB2 activates downstream effectors regulating cell proliferation and survival. A compound such as lapatinib that can selectively modulate the abnormal signaling pathways in these tumors is an alternative therapeutic approach in treating breast cancer. Although the efficacy of treatment with lapatinib in the neoadjuvant breast cancer setting is unknown at this time, tumor biomarker data generated from this study will guide future Phase II/III breast cancer efficacy trial design and biomarker studies.

Trastuzumab, a humanized monoclonal antibody targeting the extracellular domain of ErbB2, is effective in treating patients whose breast cancer overexpress ErbB2 and/or exhibit ErbB2 gene amplification. Lapatinib, a reversible inhibitor of both ErbB1 and ErbB2 tyrosine kinases, has been shown to induce growth arrest and/or tumor cell apoptosis in ErbB1 or ErbB2 dependent tumor cell lines or xenografts. Data from several Phase II and III clinical studies have demonstrated the efficacy and safety of lapatinib, as either single-agent therapy or in combination with chemotherapy, in patients with ErbB2 positive, advanced breast cancer. The dual inhibitory nature of lapatinib may offer a potential therapeutic advantage over a compound that inhibits only one tyrosine receptor kinase.

This study will examine the effect of lapatinib treatment on inhibiting ErbB1, ErbB2 and downstream signaling pathways (e.g., ERK1/2 and AKT); as well as evaluate its effect on other mediators of tumor cell growth and survival in tumor tissue from treatment-naïve breast cancer patients. Patients will receive 1500mg of lapatinib treatment daily for at least 9 days or longer and continue treatment until no more than 24 hours prior to surgical resection of the tumor. Biomarker analyses will be performed on tumor tissue obtained pre-treatment (archived tumor tissue) and compared with those biomarkers derived from the tissue obtained by resection post-treatment. In addition, intratumoral drug concentrations of lapatinib will be evaluated in the tumor tissue obtained at the time of resection.

Objective(s)

Primary

- To investigate the effects of lapatinib on mediators that regulate tumor cell growth and survival pathways (e.g., ErbB2, ERK1/2, AKT and other downstream pathways regulated by ErbB receptor signaling) through the analysis and comparison of biomarkers derived from pre-treatment and post-treatment breast tumor tissue samples.

Secondary

- To assess the safety and tolerability of lapatinib when administered to patients with treatment-naïve breast tumors.
- To examine the intratumoral concentrations of lapatinib obtained at the time of tumor resection.
- To investigate the effects of lapatinib therapy on the proteomic profile in peripheral blood.
- Pharmacogenetics may be investigated if at any time it appears that there is a potential unexpected or unexplained variation in lapatinib response or handling (e.g., safety and/or clinical activity) in this clinical study or in a series of clinical studies using lapatinib.

Endpoint(s)

Primary

- Comparison of the effects of lapatinib on biomarkers and pathways involved in regulating tumor cell proliferation and survival (e.g., ErbB2, ERK1/2, AKT, and other downstream pathways regulated by ErbB receptor signaling) in pre-treatment and post-treatment breast tumor tissue samples.

Secondary

- Evaluation of adverse events (AEs) and changes in laboratory values from pre-dose and post-dose values.
- Examination of plasma and intratumoral lapatinib steady state trough concentrations.
- Assessment of peripheral blood to examine the proteomic profile in response to lapatinib.

Study Design

This will be a single arm, open-label phase I study. Patients will receive 1500mg of lapatinib administered daily for at least 9 days.

Prior to entering the study the ErbB2 status will be required for each patient. Only patients with treatment-naïve, ErbB2 positive, as defined by either overexpression of ErbB2 (3+) by semi-quantitative IHC or amplification of the ErbB2 gene by fluorescence in situ hybridization (FISH), breast tumors measuring 1 cm or greater will be eligible for the study. For eligible patients, the archived tumor tissue obtained at diagnosis will represent the pre-treatment biopsy and be required for entry into the study. The pre-treatment biopsy tissue will be examined using technologies for measuring biomarkers involved in regulating tumor cell proliferation and survival (e.g., ErbB2, ERK1/2, AKT, and other downstream pathways regulated by ErbB receptor signaling). Patients will receive treatment with lapatinib for a minimum of 9 days prior to surgical resection. Patients will have their post-treatment surgical resection performed within 24 hours of last lapatinib dose. Tumor tissue obtained at resection will be examined for biomarkers as described above and compared with the results from the pre-treatment biopsy. In addition, tumor obtained at the time of resection will be examined for steady state, trough concentrations of lapatinib.

Safety (AEs, clinical laboratory values) will be assessed throughout the study.

Study Population

Approximately 20 evaluable patients will be enrolled from approximately 16 study centers. Female patients, aged 18 years or older with a breast tumor measuring 1 cm or greater with evidence of ErbB2 protein overexpression as measured by IHC 3+ or ErbB2 gene amplification as measured by FISH and who will receive adjuvant chemotherapy, hormonal therapy or radiation therapy will be enrolled. Patients must be naïve to cytotoxic, radiation, hormonal and biologic therapy to treat this incidence of breast cancer.

In addition to the criteria above, a patient will be eligible for inclusion only if all of the following criteria apply:

a. Non-child-bearing potential (i.e. physiologically incapable of becoming pregnant), including any female who:
   - Has had a hysterectomy
   - Has had a bilateral oophrectomy (ovariectomy)
   - Has had a bilateral tubal ligation, or
   - Is post-menopausal (a demonstration of total cessation of menses for ≥ 1 year)

b. Childbearing potential, has a negative serum pregnancy test at Screening and agrees to one of the following where considered acceptable by the institution IEC or IRB:
   - Complete abstinence form sexual intercourse from 2 weeks prior to administration of the study drug, throughout the active study treatment period, and through the follow-up visit (to occur 28 days after last dose of study medication).

- Barrier contraception (condom with spermicidal jelly, foam suppository, or film; diaphragm with spermicide; or male condom and diaphragm).
- Male partner who is sterile prior to the female subject's entry and is the sole sexual partner for that female subject.
- Implants of levonorgesterol.
- Injectable progestogen.
- Any intrauterine device (IUD) with a documented failure rate of less than 1% per year.
- Oral contraceptives (either combined or progetogen only).

c. Karnofsky Performance Status (KPS) ≥ 70 (Appendix 4)

d. Ability to swallow and retain oral medication.

e. Provided written informed consent.

f. Hemoglobin ≥ 9 gm/dL.

g. Absolute granulocyte count ≥ 1,500/mm^3 (1.5 x 10^9/L).

h. Platelet count ≥ 100,000/mm^3 (100 x 10^9/L).

i. Serum creatinine ≤ 3 mg/dL.

j. Total bilirubin ≤ 1.5mg/dL.

k. Aspartate and alanine transferase (AST & ALT) ≤ 3 times the upper limit of the reference range.

Study Assessments and Procedures

After obtaining informed consent, screening assessments will be completed to determine the patient's eligibility for enrollment. Screening assessments must be completed within 14 days prior to the first dose of investigational product. Screening assessments performed within 72 hours of the first treatment period may also be used as Day 1 assessments. The archived tumor biopsy obtained for diagnosis of this incidence of breast cancer must be available and shipped to a GSK Central lab for biomarker studies. Patients will receive study medication for a minimum of 9 days and continue treatment until scheduled surgical resection of the tumor (subjects must continue lapatinib treatment so that no more than 24 hours have elapsed between last dose and surgical resection). Surgical resection will coincide with the lapatinib trough concentration. The resected tumor tissue is required for biomarker and lapatinib intratumoral concentration analyses. A 2mL blood sample will be obtained at the time of tumor resection to examine steady state, trough plasma lapatinib concentrations. Safety (AEs, clinical laboratory values) will be assessed throughout the study.

Blood samples (2 x 5 ml tubes) will be obtained on Day 1, on the same day as the tumor resection and at the post-treatment safety visit to evaluate changes in circulating/secreted proteins (proteomic analysis) and biomarkers that may correlate with biological response at the tumor site and therefore provide an easily obtainable surrogate marker for determining therapeutic benefit of lapatinib.

A complete list of required study procedures is included in Section 14.1, Appendix 1.

Investigational Product(s)

Patients will receive oral tablets of lapatinib ditosylate salt. Lapatinib will be supplied to the study site as 250 mg tablets. The number of tablets supplied to the patient will be dependent on the period of time between enrollment and scheduled resection.

1. INTRODUCTION

1.1. Background

Advances in the understanding of the basic mechanisms driving tumor growth have implicated aberrant activation of growth factor receptors as important in many types of cancer. Overexpression of ErbB2 and epidermal growth factor receptor (EGFR, ErbB1) occurs in a significant percentage of cases in many epithelial tumors. Overexpression of either receptor is often correlated with a poor patient prognosis, including reduced overall survival.

ErbB1 and ErbB2 are members of the Type I receptor kinase family (ErbB). Tyrosine autophosphorylation of ErbB1/ErbB2 in turn activates downstream signaling cascades that promote tumor cell growth and survival. Overexpression of ErbB-family members in cells *in vitro* causes transformation, such that the cells will grow in the absence of growth factors and in anchorage-independent conditions. Cells with inactive Type I receptors do not have these properties. Inhibitors of ErbB kinases have been shown to block growth and survival signals resulting in tumor cell growth arrest and/or apoptosis in ErbB1 or ErbB2 dependent tumor cell lines and xenografts. The experimental work to support this hypothesis is summarized below

1.2. Preclinical Results

Preclinical Pharmacodynamics

Lapatinib is a specific, reversible dual inhibitor of ErbB1 and ErbB2 tyrosine kinases with IC_{50} values in an *in vitro* kinase assay of 10 and 9nM, respectively. Potent growth inhibition has been demonstrated in both *in vitro* and *in vivo* models of ErbB1 expression and/or ErbB2 overexpression. In an *in vitro* human transformed cell line (IC_{90} values ≤ 2.26μM or 1414ng/mL) no growth was observed up to 18 days after treatment with lapatinib. Treatment mice with established ErbB1 expressing or ErbB2 overexpressing human tumor xenografts resulted in the inhibition of phospho-ErbB1 and ErbB2 as well as inhibition of MAPK and PI3K/AKT downstream growth and survival pathways. These biological effects correlated with inhibition of tumor growth. Thus, the available data supports ErbB1 and ErbB2 receptor tyrosine kinase inhibition as the mechanism of action of lapatinib.

No evidence of overt central or peripheral effects in either rats or dogs, cardiovascular effects in rats, or respiratory effects in guinea pigs were noted following oral administration of up to 500mg/kilogram (kg) of lapatinib. Equivocal to very slight changes in mean systolic arterial pressure and mean arterial pressure were noted in beagle dogs at lapatinib doses of 150 and 500mg/kg. Thus, for cardiovascular effects, the no-observed adverse event level (NOAEL) in beagle dogs was 50mg/kg of lapatinib. Concentrations of lapatinib up to 2560ng/mL did not alter any other action potential parameters in isolated canine cardiac Purkinje fibers.

Preclinical Pharmacokinetics

Pharmacokinetics were investigated following single intravenous and oral administration of 10mg/kg lapatinib in mice, rats and dogs. In each species, clearance was approximately equal to the hepatic blood flow and the volume of distribution was greater than the total body water volume. The mean oral bioavailability ranged from 28.7 to 63.2% and oral half-life of lapatinib ranged from 1.0 to 4.5 hours across aforementioned species. In rat and dog plasma, lapatinib-related material (lapatinib and its metabolites) had a longer oral half-life than parent lapatinib.

Lapatinib was highly bound (>99%) to mouse, rat, dog, rabbit, and human plasma proteins at concentrations ranging from 1 to 100μM (581 to 57106 ng/mL). Species specific differences were observed in binding to erythrocytes; no erythrocyte binding was observed in rats, and binding to erythrocytes was low in mice and humans compared to rabbits and dogs.

Oral administration of lapatinib to male Wistar Han rats and male beagle dogs resulted in nearly quantitative excretion of radioactivity in feces; overall recovery was 90.0 and 89.9%, respectively. In bile duct cannulated male rats, 32% of the dose was recovered in the bile within 5 hours after dosing. Urinary excretion was < 1% of the oral dose in mice, rats and dogs.

Lapatinib showed no ability to induce CYP3A in a pregnane X receptor-screening assay. Preclinical *in vitro* studies using cDNA expressed human CYP450 enzymes and human liver microsomes have shown that lapatinib is metabolized predominantly by CYP3A4 and CYP3A5, and to a lesser extent by CYP2C19. Lapatinib was also shown to have inhibitory potential towards CYP3A4.

In a whole body autoradiography study in albino and pigmented male rats, high concentrations of radioactivity remained in the gastrointestinal tract throughout the first 24 hours after dosing. Despite the evidence for binding to melanin, no ophthalmic abnormalities or histologic findings were observed in the eye during the 2-week toxicity study in dogs.

1.3. Toxicology

No deaths were noted in either mice or rats following single oral doses of lapatinib up to 2,000 mg/kg or single intravenous doses up to 46 and 21 mg/kg, respectively. Following oral administration, reversible treatment-related changes in body weight and body weight gain (female rats only) as well as reversible gastrointestinal findings were observed. Similar degenerative and inflammatory gastrointestinal findings were noted on repeated dosing and were considered related to alterations in white blood cell count and differential as well as other hematologic parameters. Following intravenous administration, no treatment-related changes were noted.

Lapatinib was generally well tolerated when administered to rats and dogs at doses of ≤ 60 mg/kg/day and ≤ 40mg/kg/day, respectively, for up to 13 weeks. The NOAEL in rats was 60 (males) and 20 (females) mg/kg/day and in dogs was 10mg/kg/day (see Clinical

Investigator’s Brochure (CIB) [GlaxoSmithKline Document Number RM2000/00481/06] for additional information).

Lapatinib was found to be non-mutagenic and non-clastogenic in a battery of bacterial and mammalian assays which include the Ames assay, Chinese hamster ovary chromosome aberration assay, human peripheral lymphocyte chromosome aberration assay and *in vivo* rat bone marrow chromosome aberration assay.

No fetal malformations occurred when lapatinib was administered to pregnant female rats or rabbits during the period of major organogenesis. At maternally toxic doses, lapatinib treatment was associated with growth retardation and developmental variations.

An impurity 3-chloro-4-((3-fluorobenzyl) oxy) aniline has been detected in the lapatinib drug substance batches. GlaxoSmithKline does not believe the presence of this impurity increases risk for patients who are receiving, have received or will receive other therapies known to be genotoxic such as chemotherapy, hormonal therapy or radiation.

Refer to the current Supplement to the Clinical Investigator’s Brochure / Investigator’s Brochure (CIB/IB) [GlaxoSmithKline Document Number RM2000/00481/06] additional information regarding the genotoxic impurity and the results of the completed genotoxicity studies.

1.4. Preliminary Results from Healthy Volunteers and Patients

Safety data from the first single-dose healthy volunteer study (EGF10001) indicated no clinically significant changes in safety labs or ECG parameters, pre- to post-treatment. Headache, diarrhea, rash on chest, and powdery taste were the only adverse events (AEs) reported in more than one subject.

Lapatinib concentrations appeared in serum after a lag time in absorption from the oral suspension doses (10 to 250 mg) administered in the first study. Time to achieve the peak serum concentration did not meaningfully differ between doses, averaging 3 to 4 hours post dose. Area under the serum concentration versus time curve (AUC_{∞}) increased with increasing doses in a slightly more than proportional manner, especially at the lower end of the range of doses examined in this study. Geometric mean AUC ranged from 90 to 3,668 ng•h/mL, and maximum serum concentrations (C_{max}) ranged from 11 to 317 ng/mL. Half-life increased slightly with increasing dose, from 6 to 9 hours over this dose range.

Safety data from the second, repeat-dose, healthy volunteer study (EGF10002) indicated no clinically significant changes in safety labs or ECG parameters, pre- and post-treatment. Four subjects had elevations in their ALT and/or AST post-dosing Day 4. Three of the four subjects were on placebo at the time and did not receive active investigational product. The most commonly reported AEs for EGF10002 were flatulence and headache. No AEs exceeded a Grade 2 toxicity grade per National Cancer Institute Common Terminology Criteria for Adverse Events (NCI-CTCAE), Version 3 [Cancer Therapy Evaluation Program, 2003].

Pharmacokinetic data from EGF10002 indicated that serum concentrations of lapatinib increased in proportion with increasing dose, following 8 days of once daily administration of doses ranging from 25 to 175 mg. Over this dose range, mean AUC (during the Day 8 dosing interval) ranged from 326 to 2976 ng•h/mL, and mean C_{max} (occurring 3 to 4 hours post-dose) ranged from 34 to 268 ng/mL. Half-life was independent of dose, averaging 7 h on Day 1, and 9 h on Day 8. Once daily dosing resulted in approximately 50% accumulation at 100 and 175 mg doses.

The efficacy and safety of lapatinib as a single agent or in combination with chemotherapy are being assessed in several clinical studies of metastatic breast cancer.

Single agent lapatinib exhibited a 6% clinical benefit response rate in a heavily pre-treated population where an unmet medical need exist [GlaxoSmithKline Document Number RM2005/00018/00] (Study EGF20008). In a large, randomized, Phase III study EGF100151 [GlaxoSmithKline Document Number ZM2006/00137/00], lapatinib plus capecitabine demonstrated anti-tumor activity in patients with refractory, advanced metastatic breast cancer (MBC) who were previously treated with trastuzumab. Data from the pivotal study EGF100151 supported approval of lapatinib plus capecitabine for the treatment of patients with HER2-positive advanced or MBC who have received prior anthracycline, taxane and trastuzumab therapy [TYKERB Package Insert, 2007]. In this trial, lapatinib 1250mg once daily (QD) plus capecitabine 2000 mg/m2/day on days 1–14 every 3 weeks showed a statistically and clinically significant improvement in median time to progression of disease compared with capecitabine (2500 mg/m2/day on days 1–14 every 3 weeks) alone (hazard ratio 0.57; p=0.00013; median of 27.1 in the combination arm and 18.6 weeks in the control arm).

Refer to the the current Clinical Investigator's Brochure / Investigator's Brochure (CIB/IB) [GlaxoSmithKline Document Number RM2000/00481/06] for additional information on studies in healthy volunteers and cancer patients.

1.5. Rationale

Breast cancer is the most common malignancy in women in the United States. Despite a variety of hormonal, cytotoxic and biologic approaches, a significant number of tumors are resistant to currently approved treatment modalities [Ring, 2002]. There is evidence that ErbB1 and ErbB2 overexpression in breast cancers is independently associated with more aggressive tumor proliferation and a poor prognosis [Klijn, 1992; Kaptain, 2001]. ErbB1 and ErbB2 are members of the Type I family of receptor tyrosine kinases (ErbB). Tyrosine autophosphorylation of ErbB1/ErbB2 activates downstream effectors regulating cell proliferation and survival. A compound such as lapatinib that can selectively modulate the abnormal signaling pathways in ErbB1 expressing and ErbB2 overexpressing tumors is an alternative therapeutic approach in treating breast cancer. Although the efficacy of short-term treatment with lapatinib prior to surgical resection in breast cancer is unknown at this time, tumor biomarker data generated from this study will guide the design of future Phase II/III breast cancer efficacy trials and biomarker studies.

Trastuzumab, a humanized monoclonal antibody targeting ErbB2, is effective in treating patients whose breast cancer overexpress ErbB2 and/or display ErbB2 gene amplification, and hence, demonstrates the clinical utility of ErbB2 targeted therapy. Lapatinib, a reversible inhibitor of both ErbB1 and ErbB2 tyrosine kinases has been shown to induce growth arrest and/or tumor cell apoptosis in ErbB1 or ErbB2 dependent tumor cell lines or xenografts. The dual inhibitory nature of lapatinib offers a potential therapeutic advantage over a compound that inhibits only one tyrosine receptor kinase.

This study will examine the effect of lapatinib treatment on inhibiting ErbB1, ErbB2 and downstream signaling pathways (e.g., ERK1/2 and AKT); as well as evaluate its effect on other mediators of tumor cell growth and survival in tumor tissue from treatment-naïve breast cancer patients. Patients will receive 1500mg of lapatinib treatment daily for at least 9 days or longer and continue treatment until no more than 24 hours have elapsed prior to surgical resection of the tumor. Biomarker analyses will be performed on tumor tissue obtained pre-treatment (archived tumor tissue which is required for entry into the study) and compared with those biomarkers derived from the tissue obtained by resection post-treatment. In addition, intratumoral drug concentrations of lapatinib will be evaluated in the tumor tissue obtained at the time of resection.

2. OBJECTIVE(S)

2.1. Primary

- To investigate the effects of lapatinib on its target (ErbB1 and ErbB2) and mediators that regulate tumor cell growth and survival pathways (e.g., ERK1/2, AKT and other downstream pathways regulated by ErbB receptor signaling) through the analysis and comparison of biomarkers derived from pre-treatment and post-treatment breast tumor tissue samples.

2.2. Secondary

- To assess the safety and tolerability of lapatinib when administered to patients with treatment-naïve breast tumors.
- To examine the steady state, trough plasma and intratumoral concentrations of lapatinib obtained at the time of tumor resection.
- To assess the effects of lapatinib therapy on the proteomic profile in peripheral blood.
- Pharmacogenetics may be investigated if at any time it appears that there is a potential unexpected or unexplained variation in lapatinib response or handling (e.g., safety and/or clinical activity) in this clinical study or in a series of clinical studies using lapatinib.

3. ENDPOINT(S)

3.1. Primary

- Comparison of the effects of lapatinib on biomarkers involved in regulating tumor cell proliferation and survival (e.g., ErbB2, ERK1/2, AKT, and other downstream pathways regulated by ErbB receptor signaling) in pre-treatment and post-treatment breast tumor tissue samples.

3.2. Secondary

- Evaluation of adverse events (AEs) and changes in laboratory values from pre-dose and post-dose values.
- Examination of plasma and intratumoral lapatinib steady state trough concentrations.
- Assessment of peripheral blood to examine the proteomic profile in response to lapatinib.

4. STUDY DESIGN

This will be a single arm, open-label phase I study. Patients will receive 1500mg of lapatinib administered daily for a minimum of 9 days and continue treatment until surgical resection (≤24 hours).

Prior to entering the study, the documented ErbB2 status will be required for each patient. Only patients with treatment-naïve, ErbB2 positive, as defined by either overexpression of ErbB2 (3+) by semi-quantitative IHC or amplification of the ErbB2 gene by fluorescence in situ hybridization (FISH), breast tumors measuring 1 cm or greater will be eligible for the study. For eligible patients, archived tumor tissue obtained at diagnosis will represent the pre-treatment biopsy and be required for entry into the study. The pre-treatment biopsy tissue will be examined using technologies for measuring biomarkers involved in regulating tumor cell proliferation and survival (e.g., ErbB2, ERK1/2, AKT, and other downstream pathways regulated by ErbB receptor signaling). Patients will receive treatment with lapatinib for a minimum of 9 days and continue treatment until 24 hours prior to surgical resection. Patients will have their post-treatment surgical resection performed within 24 hours of last lapatinib dose. Tumor tissue obtained at resection will be examined for biomarkers as described above and compared with the results from the pre-treatment biopsy. In addition, tumor obtained at the time of resection will be examined for steady state, trough concentrations of lapatinib.

Detailed instructions on processing and shipping archived and resected tissue are included in the SRM.

Safety (AEs, clinical laboratory values) will be assessed throughout the study.

5. STUDY POPULATION

Female patients 18 years or older who are amenable to treatment with oral lapatinib and who will receive adjuvant chemotherapy, hormonal therapy or radiation therapy will be enrolled. An evaluable patient must meet all inclusion and exclusion criteria as detailed in Section 5.2 including taking all study medication. In addition, patients must have pre-dose and post-dose tumor tissue samples adequate for biomarker analysis. A GSK medical monitor must approve any deviation from these criteria.

5.1. Number of Subjects

A sufficient number of patients will be enrolled to obtain approximately 20 evaluable patients approximately 16 study centers. Evaluable subjects are defined as those subjects whose pre-treatment tumors are determined by a central laboratory to be ErbB2 positive, which is defined as either IHC 3+ or FISH amplified. Patients must have pre-dose and post-dose tumor tissue samples adequate for biomarker analyses, and have completed lapatinib treatment for a minimum of 9 days. In addition, the elapsed time between last lapatinib dose and surgical resection must be within 24 hours.

5.2. Eligibility Criteria

5.2.1. Inclusion Criteria

A subject will be eligible for inclusion in this study only if all of the following criteria apply:

1. Has a histologically confirmed, treatment-naïve, breast tumor measuring 1 cm or greater.
2. Is Female and at least 18 years of age.
3. Has tumor that overexpresses ErbB2 defined by local laboratory as either:
   - 3+ by IHC

     OR
   - c-erbB2 gene amplification by FISH
   - 1+, 2+ by IHC and c-erbB2 gene amplification by FISH.
4. Will receive adjuvant chemotherapy, hormonal therapy or radiation therapy
5. Is eligible to participate in the study if she is of:

a. Non-child-bearing potential (i.e. physiologically incapable of becoming pregnant), including any female who:
   - Has had a hysterectomy
   - Has had a bilateral oophrectomy (ovariectomy)
   - Has had a bilateral tubal ligation, or

- Is post-menopausal (a demonstration of total cessation of menses for ≥ 1 year)

b. Childbearing potential, has a negative serum pregnancy test at Screening and agrees to one of the following where considered acceptable by the institution IEC or IRB:

- Complete abstinence form sexual intercourse from 2 weeks prior to administration of the study drug, throughout the active study treatment period, and through the follow-up visit (to occur 28 days after last dose of study medication).
- Barrier contraception (condom with spermicidal jelly, foam suppository, or film; diaphragm with spermicide; or male condom and diaphragm).
- Male partner who is who is sterile prior to the female subject's entry and is the sole sexual partner for that female subject.
- Implants of levonorgesterol.
- Injectable progestogen.
- Any intrauterine device (IUD) with a documented failure rate of less than 1% per year.
- Oral contraceptives (either combined or progetogen only).

6. Is able to swallow and retain oral medication.
7. Has a Karnofsky Performance Status ≥ 70.
8. Has provided written informed consent.
9. Hemoglobin ≥ 9 gm/dL
10. Absolute granulocyte count ≥ 1,500/mm3 (1.5 x 10^9/L)
11. Platelet count ≥ 100,000/mm3 (100 x 10^9/L).
12. Serum Creatinine ≤ 3 mg/dL.
13. Total bilirubin ≤ 1.5mg/dL.

5.2.2. Exclusion Criteria

A patient will not be eligible for inclusion in this study if any of the following criteria apply:

1. Has received prior biological, cytotoxic or hormonal (other than for replacement) therapy to treat this incidence of breast cancer.
2. Has received prior radiation therapy to the chest to treat this incidence of breast cancer.
3. Is a pregnant or lactating female.
4. Has malabsorption syndrome, disease affecting gastrointestinal function, or resection of the stomach or small bowel.
5. Is considered medically unfit for the study by the PI as a result of the medical interview, physical exam, or screening investigations.

6. Has a history of drug or other allergy, which, in the opinion of the PI makes contraindicates participation.
7. Has a known immediate or delayed hypersensitivity reaction or idiosyncrasy to drugs chemically related to the study medication. These include other anilinoquinazolines, such as gefitinib [Iressa], erlotinib [Tarceva], or other chemically related compounds.
8. Has received treatment with any investigational drug in the previous 4 weeks.
9. Currently receiving treatment with any medications listed on the prohibited medication list (see Section 8.2).
10. Has had major surgery within the previous 2 weeks.
11. Aspartate or alanine transaminase (ALT or AST) greater than three times the upper limit of normal (ULN).
12. Has physiological, familial, sociological, or geographical conditions that do not permit compliance with the protocol.
13. Has Class III or IV heart failure as defined by the NYHA functional classification system.
14. Has a left ventricular ejection fraction (LVEF) < 50% based on MUGA or ECHO.
15. In the clinical judgment of the investigator or designated staff the patient has inadequate venous access for the protocol required blood draws.

5.2.3. Other Eligibility Criteria Considerations

To assess any potential impact on subject eligibility with regard to safety, the investigator must refer to the following document(s) for detailed information regarding warnings, precautions, contraindications, adverse events, and other significant data pertaining to the investigational product(s) being used in this study. Such documents include the Clinical Investigator's Brochure (CIB) [GlaxoSmithKline Document Number RM2000/00481/06].

Patient Requirements and Restrictions:

- Patients must notify the PI about any concomitant medications taken from the time of screening through study completion.

6. STUDY ASSESSMENTS AND PROCEDURES

The study specific assessments and procedures are detailed below and are outlined in the Time and Events Schedule contained in Section 14.1, Appendix 1. The total amount of blood to be obtained during this study is approximately 60 mL.

6.1. Demographic and Baseline Assessments

Demographic data will include date of birth, race, height in centimeters, and body weight in kilograms. For height and weight measurements, the patient will be allowed to wear indoor daytime clothing with no shoes.

Baseline characteristics will include a comprehensive physical examination, medical conditions, and concomitant medications. The medical and physical examination should be performed by a qualified physician and should include a thorough review of all body systems. All data will be captured in the Case Report Form (CRF) and study database.

6.2. Screening Visit

Screening assessments will be completed within 14 days prior to the first dose of study drug. Screening assessments completed within 72 hours of first dose can be used as Day 1 assessments. After consent has been obtained, patients will be required to undergo a medical screen to determine whether they are eligible to participate in the study according to the criteria listed in Section 5.2. Additionally, evaluations at the Screening visit will include:

- Full physical examination, including vital signs (blood pressure and heart rate).
- Medical history including date of diagnosis of current incidence of breast cancer, histology and current sites of disease.
- Medication history
- 12-lead ECG.
- MUGA or ECHO.
- Hematology (Section 14.2, Appendix 2).
- Clinical Chemistry including serum pregnancy test for women of childbearing potential (Section 14.2, Appendix 2).

All required information will be captured in the patient source records and documented in the CRF. Any results falling outside of the normal ranges may be repeated at the discretion of the PI.

6.3. On Study Assessments

On Day 1 and on the day prior to surgical resection patients will have safety, pharmacokinetic and pharmacodynamic assessments performed. The following procedures will be completed and time and date of each assessment will be documented in the source records and the CRF.

- Full physical examination, including vital signs (blood pressure and heart rate) to occur at Day 1 (if greater than 14 days since screening physical exam) and prior to scheduled surgical resection.
- Clinical Chemistry on Day 1 (if greater than 14 days since screening chemistries) and prior to scheduled surgical resection.
- Hematology on Day 1 (if greater than 14 days since screening hematology) and prior to scheduled surgical resection.

- A blood sample for pharmacokinetic analysis will be collected pre-dose on Day 1 and at the time of surgical resection. Refer to Section 6.6 for sampling schedule.
- Pharmacodynamic sampling (1 x 5 mL tubes of whole blood) on Day 1 and the on the day prior to scheduled resection. Refer to Section 6.7 for sampling schedule.
- Pharmacogenetic sampling (1 x 10 mL tube of whole blood) on Day 1 or at any time during the conduct of the study. Refer to Section 6.8 for sampling schedule.
- Assessment of study medication compliance when study medication bottle is returned prior to surgical resection.
- Assessment of adverse events continuously.
- Tumor surgical resection (≥Day 10).

6.4. Post Study Visit

Seven to twenty-eight days following the last dose of lapatinib (or prior to initiating adjuvant therapy), all patients will be required to have a post-study evaluation to include:

- Full physical examination, including vital signs (blood pressure and heart rate).
- Medication history.
- Assessment of Adverse Events.
- Clinical Chemistry.
- Hematology.
- Pharmacodynamic sampling (1 x 5 ml tube of whole blood).

6.5. Safety

6.5.1. Pregnancy

The investigator, or his/her designee, will collect pregnancy information on any female subject who becomes pregnant while participating in this study. The investigator, or his/her designee, will record pregnancy information on the appropriate form and submit it to GSK within 2 weeks of learning of a subject's pregnancy. The subject will also be followed to determine the outcome of the pregnancy. Information on the status of the mother and child will be forwarded to GSK. Generally, follow-up will be no longer than 6 to 8 weeks following the estimated delivery date. Any premature termination of the pregnancy will be reported.

While pregnancy itself is not considered to be an AE or SAE, any pregnancy complication or elective termination of a pregnancy for medical reasons will be recorded as an AE or a SAE, as described in Section 10.6, "Recording of AEs and SAEs" and will be followed as described in Section 10.8, "Follow-up of AEs and SAEs."

A spontaneous abortion is always considered to be a SAE and will be reported as described in Section 10, “Adverse Events (AE) and Serious Adverse Events (SAE).”

Furthermore, any SAE occurring as a result of a post-study pregnancy **and** is considered reasonably related to the investigational product by the investigator, will be reported to GSK as described in Section 10.11, "Post-study AEs and SAEs." While the investigator is not obligated to actively seek this information in former study participants, he/she may learn of an SAE through spontaneous reporting.

6.5.1.1. Pregnancy testing

All women of child-bearing potential will have a serum β-hCG test performed at screening.

As positive serum β-hCG test result at screening will exclude the patient from participation in the study (See Section 5.2).

6.5.1.2. Action to be taken if pregnancy occurs

A patient who has a positive β-hCG test result at any time after study drug administration will have study drug terminated immediately. All post-treatment study assessments will be collected as described in Section 6.5.

6.5.2. Clinical Laboratory Tests

Blood will be obtained for clinical laboratory tests prior to dosing on Days 1 and the day prior to surgical resection.

6.5.3. Cardiac Assessments

A 12-lead ECG and MUGA or ECHO will be obtained at the Screening visit.

6.5.3.1. Primary Cardiac Endpoint

The following is a definition of a primary cardiac endpoint, which includes cardiac death and congestive heart failure. This definition is provided to universally describe cardiac-related events.

- Cardiac death defined as either:
  - Cardiac death due to heart failure, myocardial infarction or arrhythmia;
  - Probable cardiac death defined as sudden, unexpected death within 24 hours of a definite or probable cardiac event.
- Severe symptomatic congestive heart failure defined as:
  - New York Heart Association (NYHA) class III or IV (refer to Appendix 5) (class III defined as being not capable of climbing one flight of stairs and class IV defined as having symptoms at rest), **and**
  - A drop in left ventricular ejection fraction (LVEF) of more than 10 points from baseline **and** below 50%.

Treatment with lapatinib will be permanently stopped if a subject develops severe symptomatic NYHA class III or IV event and has a drop in LVEF of more than 10 points. However, all efforts must be made to complete all post-study patient assessments which must include LVEF measurement. The method for LVEF assessment at screening should be used to assess LVEF in 4 weeks after documented absolute decrease of 10 points from baseline, and every 4 weeks for at least 16 weeks or until resolution.

6.5.3.2. Secondary Cardiac Endpoint

- Asymptomatic or mildly symptomatic cardiac event defined as:
  - An asymptomatic (NYHA I) or mildly symptomatic (NYHA II) significant decrease in LVEF defined as an absolute decrease in LVEF of more than 10 points from baseline **and**
  - LVEF value below 50%.

A second LVEF assessment must be performed within approximately 3 weeks to confirm the significant decrease in LVEF as defined above. If a subject has a confirmed secondary cardiac event as defined above, cardiac evaluation, preferably using the same method as for LVEF baseline assessment, must be performed every 4 weeks for at least 16 weeks or until resolution.

6.5.4. Adverse Events

Patients will be monitored from screening until 28 days after the last dose (or until initiation of adjuvant chemotherapy) for the occurrence of AEs.

6.5.5. Criteria for Study Hold

If Grade III or IV toxicity is observed in ≥ 30% (≥ 3 patients) of the first 10 patients the study will placed on hold (i.e. no further accrual). The safety data will be reviewed jointly by the GSK medical monitor and the Principal Investigators to determine if termination of additional enrollment is warranted.

6.6. Pharmacokinetics

A 2 mL blood sample for pharmacokinetic analysis will be collected predose (Day 1) and as close as possible to the time of surgical resection to examine the steady state, trough plasma lapatinib concentration.

Prior to collection, the collection tube and plasma storage tube must be labeled with the corresponding bar-coded labels provided by GSK. The sample labels will include Protocol Number, Analyte, Subject Number, Planned Sample Time and Dosing Day. The labels must be placed along the length of the tube so the bar code can be easily read. So that the tubes will fit into the autoanalyzer test tube rack, tape must not be used to secure the labels.

The 2 mL blood sample will be drawn into a vacutainer containing K3-EDTA and centrifuged to separate the plasma. The plasma (approximately 1 mL) will then be transferred into a separate storage tube for lapatinib analysis. Until assayed, the samples must be kept frozen in a freezer set at or below -20°C. All samples must be shipped with dry ice.

The actual time each sample was collected will be captured to the nearest minute on the case report form. All samples will be shipped at the end of each dosing period and analyzed for plasma lapatinib concentrations by a contract laboratory specified by GSK, using a validated method.

Pharmacokinetic sample preparation and shipping details are contained in the Study Reference Manual.

6.7. Pharmacodynamics

Blood samples (2 x 5 mL tubes) will be obtained pre-treatment (Day 1) and on the day prior to surgical resection to evaluate changes in circulating/secreted proteins (proteomic analysis) that may correlate with biological response at the tumor site and therefore provide an easily obtainable surrogate marker for determining therapeutic benefit of lapatinib. An additional 5 mL of blood will be obtained at the Post Study Visit to examine the changes in circulating/secreted proteins identified from the pharmacodynamic analyses.

Blood sample preparation and shipping details are contained in the Study Reference Manual.

6.7.1. Tumor Tissue Samples

Archived tumor tissue obtained at diagnosis (prior to initiating dosing with lapatinib) is required for biomarker analyses. All of the archived tumor tissue is required for pre-treatment biomarker analysis.

Patients will have a post-treatment surgical resection performed within 24 hours of lapatinib dose. If for any reason, surgical resection cannot be performed within 24 hours after the last dose of lapatinib, the GSK medical monitor must be contacted.

Tumor tissue processing and shipping details are contained in the Study Reference Manual.

6.8. Pharmacogenetics

6.8.1. Background

Pharmacogenetics (PGx) is the study of variability in drug handling or response due to hereditary factors in different populations. There is increasing evidence that an individual's genetic composition (i.e.genotype) may impact the pharmacokinetics (absorption, distribution, metabolism or elimination), pharmcodynamics (relationship between concentrations and pharmacologic effects or the time course of pharmacologic effects) and/or the incidence of adverse events. Some reported examples of PGx analysis include:

Drug	Disease	Gene	Outcome
6-mercaptopurine (6-MP)	Lymphoblastic leukemia	S-methyltransferase	Deficiency of the TPMT enzyme can be associated with toxicity and severe myelosuppression as patients are not able to sufficiently clear active thioguanine nucleotides [Andre, 2002; McLeod, 2002].
5-FU	Colorectal cancer	Dihydropyrimidine dehydrogenase (DPD)	Variants in the DPD gene result in little or no DPD and predisposition to toxicity to 5-FU [Daisio 2001; Kawakami, 2001; Mattison, 2002]
Atomoxetine<br><br>Desipramine	ADHD<br><br>Depression	CYP2D6	Polymorphisms in CYP2D6 result in different phenotypes; poor, intermediate, or extensive metabolizers. Poor metabolizing genotypes at risk of drug accumulation and associated toxicity [Daly, 1995]

A key component to successful PGx research is the collection of samples during the conduct of clinical studies. Collection of whole blood samples, even when no *a priori* hypothesis has been identified may enable PGx analysis to be conducted if at any time it appears that there is a potential unexpected or unexplained variation in handling or response to lapatinib. If the study patient consents to the PGx research, a 10 mL sample will be collected for the purposes of PGx research at any time during the conduct of the study. It is recommended that the sample be collected at Day 1 of study treatment.

6.8.2. Scope of PGx Analysis

PGx analysis may be conducted if unexplained variations in response to or handling of lapatinib (e.g., pharmacokinetics, efficacy and/or safety) that may be attributable to genetic variation are observed. In these circumstances, the analysis undertaken will be limited to PGx analysis of lapatinib handling or response and may include the evaluation of specific candidate genes, the conduct of a whole genome single nucleotide polymorphism (SNP) scan or other marker scan. Polymorphisms in target EGFR, ErbB2, and other relevant genes previously associated with the adverse event in question will be evaluated. For the whole genome SNP scan approach, SNP or other genetic marker sets across the genome may be evaluated to identify those markers associated with differential drug handling or response.

The need to conduct PGx analysis may be identified after a study (or set of studies) of lapatinib has been completed and the study data reviewed. For this reason, samples may be kept for up to 15 years after the last subject completes the study or GSK may destroy the samples before then. In special cases, the samples may not be studied. This might happen if there are not enough subjects, if the study is stopped for other reasons, or if no questions are raised about how people respond to or handle lapatinib.

In the performance of the PGx analysis, GSK may use subjects' medical information, samples and/or research results. This PGx research is not designed to determine whether other members of the subject's family are at risk of developing breast cancer or their response to or handling of lapatinib.

6.8.3. Sample Quality Control (QC)

If DNA is extracted from blood samples taken from the clinical study, the DNA may be subjected to sample quality control (QC) analysis. The analysis will involve the genotyping of several genetic markers to confirm the integrity of individual samples. If inconsistencies are noted in the analysis, then those samples may be destroyed.

6.8.4. Pharmacogenetics Objectives

If at any time it appears there is potential variability in lapatinib response or handling (e.g., pharmacokinetics, safety, and/or efficacy) in this clinical trial or in a series of clinical trials, the following objectives may be investigated (assuming samples number is adequate and the variability of genotyping assays):

6.8.5. Pharmacogenetics Study Population

Any subject who has given informed consent to participate in the clinical study, has met all the criteria required for entry into the clinical study, and receives investigational product may take part in the PGx research. Any subject who has received a bone marrow transplant must be excluded from the PGx research.

Subject participation in the PGx research is voluntary and refusal to participate will not indicate withdrawal from the clinical study. Refusal to participate will involve no penalty or loss of benefits to which the subject would otherwise be entitled.

No administration of investigational product beyond that detailed in the clinical study is associated with the PGx research.

6.8.6. Pharmacogenetic Samples

In addition to any blood samples taken for the clinical study, a whole blood sample (~10ml) will be collected for the PGx research using a tube containing EDTA. The PGx sample is labeled (or "coded") with a study specific number that can be traced or linked back to the subject by the investigator or site staff. Coded samples do not carry personal identifiers (such as name or social security number). The blood sample will be taken on a single occasion unless a duplicate sample is required due to inability to utilize the original sample. It is recommended that the blood sample be taken at the first available opportunity, but may be taken at any time while the subject is participating in the clinical study.

The whole blood sample must be shipped to the receiving laboratory at room temperature on the day of collection. A log should be maintained at the site capturing the time of sample collection.

Prior to sample shipment, the site staff should contact the sponsor staff member responsible for the study to notify them of the impending shipment. A completed inventory form must accompany the samples to the laboratory.

The inventory form will contain the following:

- study identifier
- investigator's name
- site address
- site number
- site and sponsor contact names and telephone numbers
- subject number(s)
- date(s) of sample collection(s)
- shipment date
- total number of tubes
- number of tubes/subject
- barcode (if applicable)
- comments (e.g., environmental conditions, information on broken/missing tubes)
- signature of packer

6.8.7. Subject Withdrawal from Pharmacogenetic Research

If a subject who has consented to participate in PGx research withdraws from the clinical study for any reason other than lost to follow-up, the subject will be given the following options concerning the PGx sample if already collected:

- PGx research continues per the subject's consent; or,
- Any remaining sample is destroyed.

6.8.8. Screen and Baseline Failures for Pharmacogenetic Samples

If a blood sample for PGx research has been collected and it is determined that the subject does not meet the inclusion and exclusion criteria for participation in the clinical study, then the investigator must complete the appropriate documentation to request sample destruction within the timeframe specified by GSK and maintain the documentation in the investigator study files.

6.8.9. Pharmacogenetic Analyses

If at any time it appears that there is a potential unexpected or unexplained variation in drug handling or response (e.g., pharmacokinetics, efficacy and/or safety) that may be attributable to genetic variation, then PGx analysis may be conducted. In these circumstances the analysis undertaken will be limited to PGx analysis of response to or handling of lapatinib.

Generally GSK will utilize two approaches to explore genetic variation in drug handling or response.

1. Hypothesis driven approach: A specific hypothesis is generated about sections of DNA (or individual single nucleotide polymorphisms (SNPs) or other genetic markers) that may be associated with differential drug handling or response. Specific sections of DNA may be selected from areas of the genome (e.g., candidate genes) known to encode the drug target, drug metabolizing enzymes, areas associated with mechanisms underlying adverse events, and those linked to study disease and, thus, linked to drug response.
2. Genome-wide approach utilizing polymorphic markers (e.g., SNPs): By evaluating large numbers of polymorphic markers throughout the genome, sets of markers may be identified that correspond to differential drug response or handling.

Analysis of genetic markers (e.g., whether within candidate genes or SNPs studied in a genome-wide analysis) will include the following considerations. The genotypic frequencies of each polymorphism will be evaluated for conformity to those expected under normal conditions by employing Hardy-Weinberg Equilibrium testing. Any departure from expectation will be taken into account, possibly signaling a data error or alternatively a connection between the polymorphism and cancer.

For pairs of polymorphisms, the degree to which alleles from the two sites are correlated (linkage disequilibrium) may also be evaluated. If the genotypes at two polymorphic

sites within a gene are shown to be statistically associated with a response to investigational product, the degree of linkage disequilibrium will aid interpretation in that it will indicate the extent to which the two sites are exerting independent effects.

A decision regarding the construction and analysis of marker haplotypes -- combinations of alleles from different polymorphic sites that are inherited from one parent -- may be guided by the assessment of linkage disequilibrium. For example, if there is no linkage disequilibrium between polymorphic sites, then haplotype construction will be uninformative.

Differences in baseline clinical characteristics and potential contributing covariates may be summarized and compared among genotype (or haplotype) subgroups.

Analyses may be carried out to evaluate the degree of association between subject genotype (or haplotype) and selected parameters (e.g., pharmacokinetics, efficacy and safety). Where such genotypic tests are inappropriate (for example, where the number of marker genotypes is too large and/or the frequency of individual genotypes too small), allelic tests may be conducted. Allelic tests evaluate whether the frequency of each marker allele is the same in responders and non-responders.

In addition to evaluating the main effects of the genotypes (haplotypes or alleles) on the selected parameters, the possibility of a treatment group by genotype (haplotype or allele) interaction may also be explored. If appropriate, the joint effects of multiple markers (gene-gene interactions) may also be evaluated.

Sample Size Considerations

The ability to detect differential drug response or handling among genotypes at a polymorphic site depends on the total number of subjects genotyped and the frequency distribution of the different genotypes. Consequently, genotyping analyses are plausible for those polymorphic sites where the number of subjects comprising the genotypic groups is sufficiently large; however, these frequencies will not be known until sufficient samples have been collected and genotyping is complete.

Estimates of sample sizes required to demonstrate genotype effects vary considerably, depending on the assumptions made about allele frequency, genetic effect size, and mechanism of inheritance [Cardon, 2000]. In the work by Palmer and Cookson [Palmer, 2001], which assumed a genotype relative risk of 1.5, it was estimated that more than 300 cases and 600 controls would be needed to conduct a genetic association analysis. In contrast, McCarthy and Hilfiker [McCarthy, 2000] showed that with a genotype relative risk of 2.16 and a relatively commonly occurring genotype, only 30 cases and 30 controls would be needed to demonstrate an association. Consequently, it is quite possible that effects with relatively large genotype relative risks may be detectable in individual Phase I or Phase II studies. A range of examples exist to demonstrate robust and clinically relevant PGx data may be generated from PGx studies with sample sizes far less than the sizes proposed by Palmer and Cookson.

Published PGx examples include abacavir [Hetherington, 2002; Mallal, 2002] hypersensitivity reaction and tranilast induced hyperbilirubinemia [Roses, 2002] where genetic markers have been found to significantly associate with hypersensitivity reaction (abacavir) and hyperbilirubinemia (tranilast). These examples show that small sample sizes typically encountered in Phase I and Phase II studies may be sufficient to identify clinically relevant genetic associations.

7. INVESTIGATIONAL PRODUCT(S)

7.1. Description of Investigational Product

Lapatinib will be supplied to the study site as 250mg oral tablets in bulk bottles containing 90 tablets per bottle. Each tablet contains 405 mg of lapatinib ditosylate monohydrate salt equivalent to 250 mg lapatinib free base.

The 250 mg tablets are oval, biconvex, orange, and film-coated with one side plain and the opposite side de-bossed with FG HLS.

The number of tablets and administration schedule will be dependent upon the number of days between enrollment and scheduled surgical resection.

7.2. Dosage and Administration

A daily dose of lapatinib is six tablets (1500mg of lapatinib) taken approximately at the same time each day. Subjects will be instructed to take study drug either 1 hour (or more) before a meal or 1 hour (or more) after a meal.

7.3. Dose Rationale

The dose of lapatinib to be tested in this study is 1500 mg QD and was selected based upon the following criteria:

- Cell-based assays demonstrate that the lapatinib concentration required to inhibit the proliferation of either ErbB1 or ErbB2 over-expressing tumor cell lines by 90% (IC_{90}) ranged from 520ng/mL to 1313ng/mL, depending upon the cell line. These concentrations were associated with 90% inhibition of tumor cell proliferation and comparable inhibition of ErbB1 or ErbB2 receptor tyrosine phosphorylation.
- In the initial Phase I patient study (EGF10003), lapatinib doses of 1200 mg and 1600 mg QD were well tolerated with only Grade 1/2 diarrhea and skin rash reported.
- Initial biopsies of skin, which expresses ErbB1 in the epidermis, show that doses below 1200 mg QD inhibit ErbB tyrosine autophosphorylation, indicating that 1500 mg QD is within in the biologically active dose range.
- Preliminary analysis of EGF10004 indicates that doses of 1200 mg QD produced biological activity against growth and survival pathways in tumor biopsies. Data

showed a plateau of effects on biomarkers (pErbB1, pErbB2, pAKT, and pERK) between doses 1200 mg and 1500 mg daily. No maximum tolerated dose was reached at the maximum dose given (1800 mg/day). Therefore, to ensure that patients received the optimum effect from lapatinib, the dose of lapatinib was increased from 1250 to 1500 mg/day, with the expectation that this change was unlikely to affect the safety profile.

- Pharmacokinetic data in patients receiving lapatinib doses up to 1800 mg QD indicate that QD dosing results in 1.5- to 2-fold accumulation and 3-fold fluctuation between peak and trough plasma concentrations at steady state. Based on these data, 1500 mg QD is likely to produce a steady-state plasma concentration profile with peaks that are above, and troughs that are below these *in vitro* IC90. Although steady-state trough concentrations at this dose may not exceed these *in vitro* IC90, data from animal xenografts indicate that intra-tumoral concentration exceeds and lags behind plasma concentration. Therefore, declining plasma concentrations in patients may not preclude continuous exposure in tumors with QD dosing.

7.4. Treatment Assignment

All patients will receive 1500 mg of lapatinib daily. Any patient not completing all required assessments will be replaced.

7.5. Packaging and Labeling

The contents of the label will be in accordance with all applicable regulatory requirements.

The study drug will be provided as open-label supplies by GSK and will contain a child resistant closure. The label will include the protocol number and storage conditions. A study pharmacist, or designee at each site will prepare study drug for patient dispensation according to assigned dose.

7.6. Handling and Storage

Study drug will be shipped to sites only after receipt of all required documents in accordance with applicable regulatory requirements and GSK procedures. Study drug should be stored at 15°C to 30°C.

Investigational product must be dispensed or administered according to procedures described herein. Only patients enrolled in the study may receive investigational product, in accordance with all applicable regulatory requirements. Only authorized site staff may supply or administer investigational product. All investigational products must be stored in a secure area with access limited to the investigator and authorized site staff and under physical conditions that are consistent with investigational product-specific requirements.

On Day 1 study drug will be administered to the patient at the study site after pre-dose safety and PK/PD assessments are complete. An adequate supply of study drug will be dispensed to the patient to account for dosing for the remainder of the study.

7.7. Product Accountability

The investigator is responsible for investigational product accountability, reconciliation, and record maintenance. In accordance with all applicable regulatory requirements, the investigator or designated site staff must maintain investigational product accountability records throughout the course of the study. The PI or designee will document the amount of investigational product received from GSK, the amount supplied and/or administered to and returned by subjects, if applicable.

After completion of the study, all unused study drug will be inventoried and packaged for return shipment.

7.8. Assessment of Compliance

Patients will be instructed to return all medication containers. The site staff will inventory the returned medication and record the amount returned on the investigational product accountability records. In addition, patients will be required to complete a patient-dosing diary which will document daily study drug dosing time. During the visit the site staff will review the patient dosing diary and will inventory returned study medication to confirm compliance with dosing.

If the investigator assesses that the patient is not compliant to the study medication regimen, the patient should be terminated from the study.

7.9. Treatment of Investigational Product Overdose

Patients with suspected overdose should be monitored until drug can no longer be detected systemically (at least 5 half-lives or 48 hours) and a follow-up physical examination with laboratory tests taken between 10 and 14 days after drug concentrations are undetectable and before being discharged from the investigator’s care. Any AE that occurs as a result of an overdose should be reported to the GSK medical monitor. There is not specific antidote for lapatinib in case of an overdose. Treatment should be based on patient signs and symptoms.

7.10. Occupational Safety

Investigational product is not expected to pose significant occupational safety risk to site staff under normal conditions of use and administration. A Material Safety Data Sheet (MSDS) describing occupational hazards and recommended handling precautions either will be provided to the investigator, where this is required by local laws, or is available upon request from GSK.

8. CONCOMITANT MEDICATIONS AND NON-DRUG THERAPIES

8.1. Permitted Medications

Patients will be asked to provide a complete list of prescription and over-the-counter medications that have been taken within 4 weeks prior to the Screening Visit. The investigator must be kept informed about any new medications taken during the course of the study. All concomitant medications taken during the study will be recorded in the CRF with indication, dose information, and dates of administration.

Patients should receive full supportive care during the trial, including transfusion of blood and blood products, treatment with antibiotics, antiemetics, antidiarrheals and analgesics, when appropriate.

8.2. Prohibited Medications

Patients should not receive other anti-cancer therapy (cytotoxic, biologic, radiation, or hormone other than for replacement) while on treatment in this study. Patients should not receive any other investigational drugs from 4 weeks prior to the first dose of lapatinb until 28 days after the last dose of study or post-treatment blood draws are completed, whichever is earlier. Lapatinib is a substrate for CYP3A4. Inducers and inhibitors of CYP3A4 may alter the metabolism of lapatinib. The following list of CYP3A4 inducers and inhibitors are prohibited from screening through discontinuation from the study:

Drug Class	Agent	Wash-out[1]
CYP3A4 Inducers		
Antibiotics	all rifamycin class agents (e.g., rifampin, rifabutin, rifapentine)	14 days
Anticonvulsants	phenytoin, carbamezepine, barbiturates (e.g., phenobarbital)	
Antiretrovirals	efavirenz, nevirapine	
Glucocorticoids (oral)	Cortisone (>50mg), hydrocortisone (>40mg), prednisone (>10mg), methylprednisolone (>8mg), dexamethasone (>1.5mg)[2]	
Other	St. John's Wort, modafinil	
CYP3A4 Inhibitors		
Antibiotics	clarithromycin, erythromycin, troleandomycin	7 days
Antifungals	itraconazole, ketoconazole, fluconazole (> 150mg daily), voriconazole	
Antiretrovirals	delaviridine, nelfinavir, amprenavir, ritonavir, indinavitr, saquinavir, lopinavir	
Calcium channel blockers	verapamil, diltiazem	
Antidepressants	nefazodone, fluvoxamine	
GI Agents	cimetidine, aprepitant	
Other	grapefruit, grapefruit juice	
	Amiodarone	6 months
Miscellaneous		
Antacids	Mylanta, Maalox, TUMS™, Rennies	1 hour before and after dosing
Herbal or dietary supplements	All	14 days

1. At the time of screening, if a patient is receiving any of the above listed medications/substances, the medication or substance must be discontinued (if clinically appropriate) for the period of time specified prior to administration of the first dose of investigational product and throughout the study period in order for the patient to meet study eligibility.
2. Glucocorticoid daily doses (oral) ≤ 1.5mg of dexamethasone (or equivalent) are allowed. Glucocorticoid conversions are provided in parentheses.

9. SUBJECT COMPLETION AND WITHDRAWAL

9.1. Subject Completion

A patient will be considered complete if the patient receives a minimum of 9 days of dosing with lapatinib and pre-dose and post-dose tumor samples are adequate for analysis of biomarkers.

9.2. Subject Withdrawal

A patient may voluntarily discontinue participation in the study at any time. The investigator may also at his or her discretion, discontinue the patient from participating in the study at any time. If a patient prematurely discontinues from study, every effort must be made to perform all post study evaluations listed in Section 6.4.

10. ADVERSE EVENTS (AE) AND SERIOUS ADVERSE EVENTS (SAE)

The investigator is responsible for the detection and documentation of events meeting the criteria and definition of an AE or SAE as provided in this protocol. During the study, when there is a safety evaluation, the investigator or site staff will be responsible for detecting AEs and SAEs, as detailed in this section of the protocol.

When applicable, AEs and SAEs will be graded using the NCI Common Terminology Criteria for Adverse Events (CTCAE), version 3. A copy of this manual is contained in the Study Reference Manual.

10.1. Definition of an AE

Any untoward medical occurrence in a patient or clinical investigation subject, temporally associated with the use of a medicinal product, whether or not considered related to the medicinal product.

An AE can therefore be any unfavorable and unintended sign (including an abnormal laboratory finding), symptom, or disease (new or exacerbated) temporally associated with the use of a medicinal product.

Examples of an AE **include**:

Exacerbation of a chronic or intermittent pre-existing condition including either an increase in frequency and/or intensity of the condition.

New conditions detected or diagnosed after investigational product administration even though it may have been present prior to the start of the study.

Signs, symptoms, or the clinical sequelae of a suspected interaction.

Signs, symptoms, or the clinical sequelae of a suspected overdose of either investigational product or a concurrent medication (overdose per se should not be reported as an AE/SAE).

Examples of an AE **do not include** a/an:

Medical or surgical procedure (e.g., endoscopy, appendectomy); the condition that leads to the procedure is an AE.

Situations where an untoward medical occurrence did not occur (social and/or convenience admission to a hospital).

Anticipated day-to-day fluctuations of pre-existing disease(s) or condition(s) present or detected at the start of the study that do not worsen.

The disease/disorder being studied, or disease progression, signs, or symptoms of the disease/disorder being studied, unless more severe than expected for the subject's condition.

For GSK clinical studies, AEs may include pre- or post-treatment events that occur as a result of protocol-mandated procedures (i.e., invasive procedures, modification of subject's previous therapeutic regimen).

10.2. Definition of a SAE

A serious adverse event is any untoward medical occurrence that, at any dose:

a. Results in death.

b. Is life-threatening.

   NOTE: The term 'life-threatening' in the definition of 'serious' refers to an event in which the subject was at risk of death at the time of the event. It does not refer to an event, which hypothetically might have caused death, if it were more severe.

c. Requires hospitalization or prolongation of existing hospitalization.

   NOTE: In general, hospitalization signifies that the subject has been detained (usually involving at least an overnight stay) at the hospital or emergency ward for observation and/or treatment that would not have been appropriate in the physician's office or out-patient setting. Complications that occur during hospitalization are AEs. If a complication prolongs hospitalization or fulfills any other serious criteria, the event is serious. When in doubt as to whether "hospitalization" occurred or was necessary, the AE should be considered serious.

   Hospitalization for elective treatment of a pre-existing condition that did not worsen from baseline is not considered an AE.

d. Results in disability/incapacity, or

   NOTE: The term disability means a substantial disruption of a person's ability to conduct normal life functions. This definition is not intended to include experiences of relatively minor medical significance such as uncomplicated headache, nausea, vomiting, diarrhea, influenza, and accidental trauma (e.g. sprained ankle) which may interfere or prevent everyday life functions but do not constitute a substantial disruption.

e. Is a congenital anomaly/birth defect.

f. Medical or scientific judgment should be exercised in deciding whether reporting is appropriate in other situations, such as important medical events that may not be immediately life-threatening or result in death or hospitalization but may jeopardize

the subject or may require medical or surgical intervention to prevent one of the other outcomes listed in the above definition. These should also be considered serious. Examples of such events are invasive or malignant cancers, intensive treatment in an emergency room or at home for allergic bronchospasm, blood dyscrasias or convulsions that do not result in hospitalization, or development of drug dependency or drug abuse.

Additional protocol defined criteria:

- All grade 4 laboratory abnormalities.
- Cardiovascular events have been seen in patients taking other compounds that inhibit ErbB2 when used in combination with or following anthracyclines and interstitial pneumonitis has been reported in patients taking compounds that inhibit ErbB1. As a precaution, the following will be reported as SAE:
    - Cardiac related events meeting the criteria of a primary cardiac endpoints (which is, CHF and cardiac death) will be reported as serious adverse events (SAEs).

      Note: If a secondary cardiac endpoint fulfils any of the criteria for ‘seriousness’ (e.g. hospitalisation), it must also be reported as an SAE. **Symptomatic** declines in LVEF that do not meet the criteria for a secondary cardiac endpoint are not expected to be common. These events will be reported as AEs or SAEs, if applicable, as described above. **Asymptomatic** declines in LVEF that do not meet the criteria for ‘significant,’ do not qualify as a secondary cardiac endpoint and will not be reported as AEs.

Any signs or symptoms of pneumonitis that are ≥ Grade 3 (NCI CTCAE, version 3.0) (defined as radiographic changes and requiring oxygen). Refer to [Cancer Therapy Evaluation Program, 2003] CTCAE (version 3.0) grading of pneumonitis/pulmonary infiltrates.

10.3. Lack of Efficacy

“Lack of efficacy” per se will not be reported as an AE. The signs and symptoms or “clinical sequelae resulting from lack of efficacy will be reported if they fulfil the AE or SAE definition (including clarifications).

10.4. Clinical Laboratory Abnormalities and Other Abnormal Assessments as AEs and SAEs

Abnormal laboratory findings (e.g., clinical chemistry, hematology, urinalysis) or other abnormal assessments (e.g., ECGs, vital signs, etc.) that are judged by the investigator as **clinically significant** will be recorded as AEs or SAEs if they meet the definition of an AE, as defined in Section 10.1 (“Definition of an AE”), or SAE, as defined in Section 10.2 (“Definition of a SAE”). Clinically significant abnormal laboratory findings or other abnormal assessments that are detected during the study or are present at baseline and significantly worsen following the start of the study will be reported as AEs and SAEs. However, clinically significant abnormal laboratory findings or other abnormal

assessments that are associated with the disease being studied, unless judged by the investigator as more severe than expected for the patient's condition, or that are present or detected at the start of the study and do not worsen, will **not** be reported as AEs or SAEs.

The investigator will exercise his or her medical and scientific judgment in deciding whether an abnormal laboratory finding or other abnormal assessment is clinically significant.

10.5. Time Period, Frequency, and Method of Detecting AEs and SAEs

Patients will be monitored for the occurrence of AEs/SAEs at each study visit or contact, and at the 28-day post study follow-up visit. The investigator or designee will inquire about AEs by asking the following general questions:

1. How are you feeling?
2. Have you had any medical problems since your last visit?
3. Have you taken any new medications since your last visit?

All AEs and SAEs, regardless of relationship to study medications, will be recorded from study treatment initiation until 72 hours after permanent discontinuation of study treatment. Only AEs and SAEs assessed and related to study medications will be recorded between 72 hours and 28 days after the permanent discontinuation of study treatment.

10.6. Recording of AEs and SAEs

When an AE/SAE occurs, it is the responsibility of the investigator to review all documentation (e.g., hospital progress notes, laboratory, and diagnostics reports) relative to the event. The investigator will then record all relevant information regarding an AE/SAE on the CRF. It is not acceptable for the investigator to send photocopies of the subject's medical records to GSK in lieu of completion of the appropriate AE/SAE CRF pages. However, there may be instances when copies of medical records for certain cases are requested by GSK. In this instance, all subject identifiers will be blinded on the copies of the medical records prior to submission to GSK.

The investigator will attempt to establish a diagnosis of the event based on signs, symptoms, and/or other clinical information. In such cases, the diagnosis should be documented as the AE/SAE and not the individual signs/symptoms.

10.7. Evaluating AEs and SAEs

10.7.1. Assessment of Intensity

The investigator will make an assessment of intensity for each AE and SAE reported during the study. The assessment will be based on the investigator's clinical judgment and using the Cancer Evaluation Program, Common Terminology Criteria for Adverse Events(CTCAE), Version 3, when appropriate. For non-CTCAE graded events, the intensity of each AE and SAE recorded in the CRF should be assigned to one of the following categories:

Mild: An event that is easily tolerated by the subject, causing minimal discomfort and not interfering with everyday activities.

Moderate: An event that is sufficiently discomforting to interfere with normal everyday activities.

Severe: An event that prevents normal everyday activities.

An AE that is assessed as severe should not be confused with a SAE. Severity is a category utilized for rating the intensity of an event; and both AEs and SAEs can be assessed as severe. An event is defined as 'serious' when it meets one of the pre-defined outcomes as described in Section 10.2, "Definition of a SAE".

10.7.2. Assessment of Causality

The investigator is obligated to assess the relationship between investigational product and the occurrence of each AE/SAE. The investigator will use clinical judgment to determine the relationship. Alternative causes, such as natural history of the underlying diseases, concomitant therapy, other risk factors, and the temporal relationship of the event to the investigational product will be considered and investigated. The investigator will also consult the CIB/IB and/or Product Information, for marketed products, in the determination of his/her assessment.

There may be situations when an SAE has occurred and the investigator has minimal information to include in the initial report to GSK. However, it is very important that the investigator always make an assessment of causality for every event prior to transmission of the SAE CRF to GSK. The investigator may change his/her opinion of causality in light of follow-up information, amending the SAE CRF accordingly. The causality assessment is one of the criteria used when determining regulatory reporting requirements.

The investigator will provide the assessment of causality as per instructions on the SAE form in the CRF.

10.8. Follow-Up of AEs and SAEs

After the initial AE/SAE report, the investigator is required to proactively follow each subject and provide further information to GSK on the subject's condition.

All AEs and SAEs documented at a previous visit/contact and are designated as ongoing, will be reviewed at subsequent visits/contacts.

All AEs and SAEs will be followed until resolution, until the condition stabilizes, until the event is otherwise explained, or until the subject is lost to follow-up. Once resolved, the appropriate AE/SAE CRF page(s) will be updated. The investigator will ensure that follow-up includes any supplemental investigations as may be indicated to elucidate the nature and/or causality of the AE or SAE. This may include additional laboratory tests or investigations, histopathological examinations, or consultation with other health care professionals.

GSK may request that the investigator perform or arrange for the conduct of supplemental measurements and/or evaluations to elucidate as fully as possible the nature and/or causality of the AE or SAE. The investigator is obligated to assist. If a subject dies during participation in the study or during a recognized follow-up period, GSK will be provided with a copy of any post-mortem findings, including histopathology.

New or updated information will be recorded on the originally completed "SAE" CRF, with all changes signed and dated by the investigator. The updated SAE CRF should be resent to GSK within the time frames outlined in Section 10.9.

10.9. Prompt Reporting of SAEs to GSK

SAEs will be reported promptly to GSK as described in the following table once the investigator determines that the event meets the protocol definition of an SAE.

10.9.1. Timeframes for Submitting SAE Reports to GSK

	Initial SAE Reports		Follow-up Information on a Previously Reported SAE	
Type of SAE	Time Frame	Documents	Time Frame	Documents
All SAEs	24 hrs	"SAE" CRF pages	24 hrs	Updated "SAE" CRF pages

10.9.2. Completion and Transmission of the SAE Reports

Once an investigator becomes aware that an SAE has occurred in a study subject, she/he will report the information to GSK within 24 hours as outlined in Section 10.9, "Prompt Reporting of SAEs to GSK". The SAE CRF will always be completed as thoroughly as possible with all available details of the event, signed by the investigator (or designee), and forwarded to GSK within the designated time frames. If the investigator does not have all information regarding an SAE, he/she will not wait to receive additional

information before notifying GSK of the event and completing the form. The form will be updated when additional information is received.

The investigator will always provide an assessment of causality at the time of the initial report as described in Section 10.7.2, “Assessment of Causality”.

Facsimile transmission of the “SAE” CRF is the preferred method to transmit this information to the project contact for SAE receipt. In rare circumstances and in the absence of facsimile equipment, notification by telephone is acceptable, with a copy of the "SAE" CRF sent by overnight mail. Initial notification via the telephone does not replace the need for the investigator to complete and sign the SAE CRF within the time frames outlined in Section 10.9, “Prompt Reporting of SAEs to GSK”.

GSK will provide a list of project contacts for SAE receipt, fax numbers, telephone numbers, and mailing addresses.

The following pages of the CRF must accompany the SAE forms that are forwarded to GSK: “Demography”, “Medical History”, “Concomitant Medications”, “Study Medication Records”, and “Form D” (if applicable).

10.10. Regulatory Reporting Requirements for SAEs

The investigator will promptly report all SAEs to GSK in accordance with the procedures detailed in Section 10.9, "Prompt Reporting of SAEs to GSK." GSK has a legal responsibility to notify, as appropriate, both the local regulatory authority and other regulatory agencies about the safety of a product under clinical investigation. Prompt notification of SAEs by the investigator to the appropriate project contact for SAE receipt is essential so that legal obligations and ethical responsibilities towards the safety of other subjects are met.

The investigator, or responsible person according to local requirements, will comply with the applicable local regulatory requirements related to the reporting of SAEs to regulatory authorities and the Institutional Review Board (IRB)/Independent Ethics Committee (IEC).

This protocol has been filed under an Investigational New Drug (IND) application with the US Food and Drug Administration (FDA). A given SAE may qualify as an IND Safety Report if the SAE is both attributable to the investigational product and unexpected. In this case, all investigators filed to the IND (and associated INDs for the same compound) will receive an Expedited Investigator Safety Report (EISR), identical in content to the IND Safety Report submitted to the FDA.

Expedited Investigator Safety Reports (EISR) are prepared according to GSK policy and are forwarded to investigators as necessary. An EISR is prepared for a SAE that is both attributable to investigational product and unexpected. The purpose of the EISR is to fulfill specific regulatory and Good Clinical Practice (GCP) requirements, regarding the product under investigation.

An investigator who receives an EISR describing a SAE or other specific safety information from GSK will file it with the Investigator Brochure and will notify the IRB or IEC, if appropriate according to local requirements.

10.11. Post-study AEs and SAEs

A post-study AE/SAE is defined as any event that occurs outside of the AE/SAE detection period defined in Section 10.5, “Time Period, Frequency, and Method of Detecting AEs and SAEs”, of the protocol.

Investigators are not obligated to actively seek AEs or SAEs in former study participants. However, if the investigator learns of any SAE, including a death, at any time after a subject has been discharged from the study, and he/she considers the event reasonably related to the investigational product, the investigator will promptly notify GSK.

10.12. SAEs Related to Study Participation

An SAE considered related to study participation (e.g., procedures, invasive tests, a change in existing therapy), even if it occurs during the pre- or post-treatment period, will be reported promptly to GSK (see Section 10.9, "Prompt Reporting of SAEs to GSK").

11. DATA ANALYSIS AND STATISTICAL CONSIDERATIONS

11.1. Hypotheses

No formal hypotheses are being tested. The study will focus on obtaining point estimates and 90% confidence intervals of biomarker differences derived from the pre-to-post tumor tissue.

11.2. Sample Size Considerations

Twenty evaluable subjects are needed for biomarker analyses.

11.2.1. Sample Size Assumptions

In preliminary analysis of the tumor biomarkers in the phase I clinical study EGF10004, the standard deviation of the pre-dose to post-dose percent change in individual biomarkers ranged from 17% to over 600% across multiple dose levels of lapatinib. Although the variability of tumor biomarkers measured in a more homgenous population (i.e. all breast cancer) may be reduced, it is not practical to frame this study as confirmatory hypothesis testing. Thus, results from this study should be viewed as exploratory data analysis, and proposed sample sizes are based on logistic feasibility.

11.3. Analysis Populations

All subjects who receive at least one dose of lapatinib will be included in the safety population. All patients for whom valid lapatinib pharmacokinetic concentrations can be estimated will be included in the pharmacokinetic population. All patients who receive at least 9 days of treatment and for whom pre- and post-dose biomarker values have been obtained will be included in the pharmacodynamic population.

11.4. General Considerations for Data Analysis

Data will be listed and summarized according to GSK reporting standards, where applicable. Complete details will be documented in the Reporting and Analysis Plan (RAP).

11.4.1. Withdrawal

Patients that drop out will be replaced until 20 subjects have been determined to be evaluable.

11.4.2. Missing Data

Missing data will not be imputed.

11.4.3. Assessment Windows

Safety assessments that occur prior to the administration of the first administration of lapatinib will be considered screening assessments. Safety assessments that occur after dosing has begun will be considered as having occurred while on treatment. Pharmacokinetic measurements will be associated with the treatment day on which the assessment occurred. Pharmacodynamic measures will be associated with the day on which the measure was taken.

11.4.4. Other issues

Since multiple sites will be involved in the conduct of this study, an exploratory analysis may be undertaken to determine if there appear to be gross site effects associated with the primary endpoints.

11.5. Safety Analyses

Safety assessments conducted at screening and at various times throughout the study include blood pressure, heart rate, hematology and clinical chemistry. Adverse events will be monitored throughout the study.

11.5.1. Extent of Exposure

Extent of exposure to lapatinib will depend on the patient’s tolerability to the dose administered and the date post-exposure biopsy or resection.

11.5.2. Adverse Events

The investigator is responsible for the detection and documentation of events meeting the definition of an AE or SAE as provided in this protocol. Whenever there is a safety evaluation, the investigator or study site personnel will be responsible for detecting AEs and SAEs, as detailed in Section 10 of the protocol. In order to fulfill international safety reporting obligations, the investigator should include in his or her assessment any SAEs resulting from study participation (e.g., complications resulting from the taking of a blood sample).

Adverse Events will be coded and grouped using the Medical Dictionary for Regulatory Activities (MedDRA). All AEs will be listed. If warranted, by the number of AEs, a summary by treatment group of the number and percent of patients reporting each event at least once will be produced.

11.5.3. Clinical Laboratory Evaluations

Hematology and clinical chemistry values will be listed for each patient and flagged high or low relative to the normal range, where applicable. Median and range pre- and post-dose values will be plotted by treatment group. Pre-dose values will be used to assess laboratory shifts occurring post-dose. A comparison of pre-study and post-study follow-up values will be performed to identify any parameters that have not returned to pre-study levels.

11.5.4. Other Safety Measures

Vital signs will be listed for each subject. Median profiles will be plotted by treatment group.

11.6. Clinical Pharmacology Data Analyses

11.6.1. Pharmacokinetic Analyses

Lapatinib plasma and intratumoral concentration data will be summarized and displayed in both tabular and graphical form.

11.6.2. Pharmacodynamic Analyses

Each patient will be measured for biomarker activity level in tumor obtained pre- and post-dose. For each marker, a one-way analysis of covariance of the post-dose measurement will be performed with a biological marker of cellular proliferation, cellular apoptosis, or other biological marker deemed relevant to pathways regulating cancer

pathophysiology as a classification variable and the pre-dose level of the marker as a covariate. Transformation, e.g., logarithms, of pre- and post-dose marker levels may be necessary to satisfy analysis assumptions.

All pharmacodynamic endpoints of interest from all study sessions will be descriptively and/or graphically summarized, as appropriate to the data.

12. STUDY ADMINISTRATION

12.1. Regulatory and Ethical Considerations

12.1.1. Regulatory Authority Approval

GSK will obtain approval to conduct the study from the appropriate regulatory agency in accordance with any applicable country-specific regulatory requirements prior to a site initiating the study in that country.

12.1.2. Ethical Conduct of the Study and Ethics Approval

This study will be conducted in accordance with "good clinical practice" (GCP) and all applicable regulatory requirements, including, where applicable, the 1996 version of the Declaration of Helsinki.

The investigator (or sponsor, where applicable) is responsible for ensuring that this protocol, the site's informed consent form, and any other information that will be presented to potential patients (e.g., advertisements or information that supports or supplements the informed consent) are reviewed and approved by the appropriate IEC/IRB. The investigator agrees to allow the IEC/IRB direct access to all relevant documents. The IEC/IRB must be constituted in accordance with all applicable regulatory requirements. GSK will provide the investigator with relevant document(s)/data that are needed for IEC/IRB review and approval of the study. Before investigational product(s) and CRFs can be shipped to the site, GSK must receive copies of the IEC/IRB approval, the approved informed consent form, and any other information that the IEC/IRB has approved for presentation to potential subjects.

If the protocol, the informed consent form, or any other information that the IEC/IRB has approved for presentation to potential subjects is amended during the study, the investigator is responsible for ensuring the IEC/IRB reviews and approves, where applicable, these amended documents. The investigator must follow all applicable regulatory requirements pertaining to the use of an amended informed consent form including obtaining IEC/IRB approval of the amended form before new subjects consent to take part in the study using this version of the form. Copies of the IEC/IRB approval of the amended informed consent form/other information and the approved amended informed consent form/other information must be forwarded to GSK promptly.

12.1.3. Informed Consent

Informed consent will be obtained before the subject can participate in the study. The contents and process of obtaining informed consent will be in accordance with all applicable regulatory requirements.

12.1.4. Investigator Reporting Requirements

As indicated in Section 10.10, the investigator (or sponsor, where applicable) is responsible for reporting SAEs to the IEC/IRB, in accordance with all applicable regulations. Furthermore, the investigator may be required to provide periodic safety updates on the conduct of the study at his or her site and notification of study closure to the IEC/IRB. Such periodic safety updates and notifications are the responsibility of the investigator and not of GSK.

12.2. Study Monitoring

In accordance with applicable regulations, GCP, and GSK procedures, GSK monitors will contact the site prior to the subject enrollment to review the protocol and data collection procedures with site staff. In addition, the monitor will periodically contact the site, including conducting on-site visits. The extent, nature and frequency of on-site visits will be based on such considerations as the study objective and/or endpoints, the purpose of the study, study design complexity, and enrollment rate.

During these contacts, the monitor will:

- Check the progress of the study.
- Review study data collected.
- Conduct source document verification.
- Identify any issues and address their resolution.

This will be done in order to verify that the:

- Data are authentic, accurate, and complete.
- Safety and rights of subjects are being protected.
- Study is conducted in accordance with the currently approved protocol (and any amendments), GCP, and all applicable regulatory requirements.

The investigator agrees to allow the monitor direct access to all relevant documents and to allocate his/her time and the time of his/her staff to the monitor to discuss findings and any relevant issues.

At study closure, monitors will also conduct all activities described in Section 12.4, "Study and Site Closure."

12.3. Quality Assurance

To ensure compliance with GCP and all applicable regulatory requirements, GSK may conduct a quality assurance audit. Regulatory agencies may also conduct a regulatory inspection of this study. Such audits/inspections can occur at any time during or after completion of the study. If an audit or inspection occurs, the investigator and institution agree to allow the auditor/inspector direct access to all relevant documents and to allocate his/her time and the time of his/her staff to the auditor/inspector to discuss findings and any relevant issues.

12.4. Study and Site Closure

Upon completion of the study, the monitor will conduct the following activities in conjunction with the investigator or site staff, as appropriate:

- Return of all study data to GSK.
- Completion and return of all data queries to GSK.
- Accountability, reconciliation, and arrangements for unused investigational product(s) to be returned to GSK.
- Review of site study records for completeness.
- Shipment of PK/PD/biomarker samples to assay laboratory(ies).

In addition, GSK reserves the right to temporarily suspend or prematurely discontinue this study either at a single site or at all sites at any time for reasons including, but are not limited to, safety or ethical issues or severe non-compliance. If GSK determines such action is needed, GSK will discuss this with the Investigator (including the reasons for taking such action) at that time. When feasible, GSK will provide advance notification to the investigator of the impending action prior to it taking effect.

GSK will promptly inform all other investigators and/or institutions conducting the study if the study is suspended or terminated for safety reasons, and will also inform the regulatory authorities of the suspension or termination of the study and the reason(s) for the action. If required by applicable regulations, the investigator must inform the IEC/IRB promptly and provide the reason for the suspension or termination.

If the study is prematurely discontinued, all study data must be returned to GSK. In addition, arrangements will be made for all unused investigational product(s) in accordance with the applicable GSK procedures for the study.

Financial compensation to investigators and/or institutions will be in accordance with the agreement established between the investigator and GSK.

12.5. Records Retention

Following closure of the study, the investigator must maintain all site study records in a safe and secure location. The records must be maintained to allow easy and timely retrieval, when needed (e.g., audit or inspection), and, whenever feasible, to allow any subsequent review of data in conjunction with assessment of the facility, supporting systems, and staff. Where permitted by local laws/regulations or institutional policy, some or all of these records can be maintained in a format other than hard copy (e.g., microfiche, scanned, electronic); however, caution needs to be exercised before such action is taken. The investigator must assure that all reproductions are legible and are a true and accurate copy of the original and meet accessibility and retrieval standards, including re-generating a hard copy, if required. Furthermore, the investigator must ensure there is an acceptable back-up of these reproductions and that an acceptable quality control process exists for making these reproductions.

GSK will inform the investigator of the time period for retaining these records to comply with all applicable regulatory requirements. The minimum retention time will meet the strictest standard applicable to that site for the study, as dictated by any institutional requirements or local laws or regulations, or GSK standards/procedures; otherwise, the retention period will default to 15 years.

The investigator must notify GSK of any changes in the archival arrangements, including, but not limited to, the following: archival at an off-site facility, transfer of ownership of the records in the event the investigator leaves the site.

12.6. Provision of Study Results and Information to Investigators

When a clinical study report is completed, GSK will provide the major findings of the study to the investigator.

In addition, details of the study treatment assignment will be provided to the investigator to enable him/her to review the data to determine the outcome of the study for his/her subject.

12.7. Information Disclosure and Inventions

Ownership

All information provided by GSK and all data and information generated by the site as parts of the study (other than a subject’s medical records) are the sole property of GSK.

All rights, title, and interests in any inventions, know-how or other intellectual or industrial property rights which are conceived or reduced to practice by site staff during the course of or as a result of the study are the sole property of GSK, and are hereby assigned to GSK.

If a written contract for the conduct of the study which includes ownership provisions inconsistent with this statement is executed between GSK and the study site, that contract’s ownership provisions shall apply rather than this statement.

Confidentiality

All information provided by GSK and all data and information generated by the site as part of the study (other than a subject’s medical records) will be kept confidential by the investigator and other site staff. This information and data will not be used by the investigator or other site personnel for any purpose other than conducting the study. These restrictions do not apply to: (1) information which becomes publicly available through no fault of the investigator or site staff; (2) information which it is necessary to disclose in confidence to an IEC or IRB solely for the evaluation of the study; (3) information which it is necessary to disclose in order to provide appropriate medical care to a study subject; or (4) study results which may be published as described in the next paragraph. If a written contract for the conduct of the study which includes confidentiality provisions inconsistent with this statement is executed, that contract’s confidentiality provisions shall apply rather than this statement.

Publication

For multicenter studies, the first publication or disclosure of study results shall be a complete, joint multicenter publication or disclosure coordinated by GSK. Thereafter, any secondary publications will reference the original publication(s).

Prior to submitting for publication, presentation, use for instructional purposes, or otherwise disclosing the study results generated by the site (collectively, a “Publication”), the investigator shall provide GSK with a copy of the proposed Publication and allow GSK a period of at least thirty (30) days [or, for abstracts, at least five (5) working days] to review the proposed Publication. Proposed Publications shall not include either GSK confidential information other than the study results or personal data on any subject, such as name or initials.

At GSK’s request, the submission or other disclosure of a proposed Publication will be delayed a sufficient time to allow GSK to seek patent or similar protection of any inventions, know-how or other intellectual or industrial property rights disclosed in the proposed Publication.

If a written contract for the conduct of the study, which includes publication provisions inconsistent with this statement is executed, that contract’s publication provisions shall apply rather than this statement.

This includes the results of the pharmacogenetics assessments included in the study and all samples collected for those assessments.

12.8. Data Management

Subject data are collected by the investigator or designee using the Case Report Form (CRF) defined by GSK. Subject data necessary for analysis and reporting will be entered/transmitted into a validated database or data system. Clinical data management will be performed in accordance with applicable GSK standards and data cleaning procedures. Database freeze will occur when data management quality control procedures are completed. Original CRFs will be retained by GSK, while the investigator will retain a copy.

Pharmacogenetic Data Management

Data from the case report forms and PGx research using the coded sample will be stored electronically. International regulations for information on computers and relevant laws on processing personal information will be followed.

Data Security

Access to the data will be strictly controlled.

12.9. Confidentiality of a Subject's Pharmacogenetic Data

GSK advises that participation in this PGx research, withdrawal from this research, sample destruction, and/or PGx results should not be documented in the subject's medical records. Storage of information regarding the PGx research with source documents for the study is permissible if stored in the investigator study files.

Coded PGx samples and results will be associated with the subject's study specific number in computer databases. Coded PGx research results may be submitted to regulatory agencies as part of an investigational product submission and/or included in a research publication.

Individual genotype results will only be shared with a subject through the investigator if the subject requests to see their results and it is a requirement of a governmental agency or other legal authority that GSK make these results available. GSK will not release individual PGx results to anyone else (e.g., family members, primary care physicians, insurers, or employers) under any circumstance, unless required by law.

13. REFERENCES

Andre BP, Kuilenburg, V, Meinsma, R, Zoetekouw L, Van Gennip AH. High prevalence of the IVS14 + 1G>A mutation in the dihydropyrimidine dehydrogenase gene of patients with severe 5-fluorouracil-associated toxicity. Pharmacogenetics 2002; 12:555-558

Cancer Therapy Evaluation Program, Common Terminology Criteria for Adverse Events. Version 3.0., DCTD. NCI, HIH, DHHS, December 2003.

GlaxoSmithKline Document Number RM2000/00481/06. GW572016 Current Investigator's Brochure. Report Date 06-Apr-2007.

GlaxoSmithKline Document Number RZM20005/00115/00 Study ID EGF10027. Supplement to GW572016 Investigator's Brochure. Report Date 21-Sep-2005.

Cardon LR, Idury RM, Harris TJR, Witte JS, Elston RC. Testing drug response in the presence of genetic information: sampling issues for clinical trials. *Pharmacogenetics*. 2000; 10:503-10.

Daisio R. Clinical implications of dihydropyrimidine dehydrogenase on 5-FU pharmacology. Oncology 2001; 15(Suppl 2):21-26.

Daly AK. Molecular basis of polymorphic drug metabolism. J Mol Med. 1995; 73:539-553.

Dudoit S, Fridlyand J, Speed T. Comparison of discrimination methods for the classification of tumors using gene expression data. Technical report #576. June 2002.

GlaxoSmithKline Document Number RM2005/00018/00 Study ID EGF20008. An Open-Label Multicenter, Single Arm Phase II Study of Oral GW572016 as Single Agent Therapy in Subjects with Advanced or Metastatic Breast Cancer Who Have Progress While Receiving Herceptin Containing Regimens. Report Date 13-Jul-2006.

GlaxoSmithKline Document Number ZM2006/00137/00 Study ID EGF100151. A Phase III, Randomized, Open-Label, Multicenter Study Comparing GW572016 and Capecitabine (Xeloda) versus Capecitabine in Women with Refractory Advanced or Metastatic Breast Cancer. Report Date 08-Sep-2006.

Hetherington S, Hughes AR, Mosteller M, Shortino D, Baker KL, Spreen W, Lai E, Davies K, Handley A, Dow DJ, Fling ME, Stocum M, Bowman C, Thurmond LM, Roses AD. Genetic variations in HLA-B region and hypersensitivity reactions to abacavir. *Lancet*. 2002; 359:1121-2.

Kaptain S, Tan L, Chen B, Her-2/*neu* and Breast Cancer. Diagnostic Molecular Pathology. 10(3): 139-152, 2001.

Kawakami K, Salonga D, Park JM, Danenberg KD, Uetake H, Brabender J et al. Different Lengths of a Polymorphic Repeat Sequence in the Thymidylate Synthase Gene Affect Translational Efficiency but Not Its Gene Expression. Clin Cancer Res 2001:7(12):4096.

Klijn JG, Berns PM, Schmitz PI, et al. The clinical significance of epidermal growth factor receptor (EGF-R)in human breast cancer: a review on 5232 patients. Endocrine Review13:3-17, 1992.

Mallal S, Nolan D, Witt C, Masel G, Martin AM, Moore C, Sayer D, Castley A, Mamotte C, Maxwell D, James I. Association between presence of HLA-B*5701, HLA-DR7, and HLA-DQ3 and hypersensitivity to HIV-1 reverse-transcriptase inhibitor abacavir. *Lancet*. 2002; 359:727-32.

Mattison L, Soong R, Daisio R. Implications of dihydropyrimidine dehydrogenase on 5-fluorouracil pharmacogenetics and pharmacogenomics. Pharmacogenomics 2002; 3(4):485-492.

McCarthy JJ, Hilfiker R. The use of single-nucleotide polymorphism maps in pharmacogenomics. *Nat Biotechnol*. 2000; 18:505-8.

McLeod HL, Siva C. The thiopurine S-methyltransferase gene locus – implications for clinical pharmacogenomics. Pharmacogenomics 2002; 3(1)89-98.

Palmer LJ, Cookson WO. Using single nucleotide polymorphisms as a means to understanding the pathophysiology of asthma. *Respir Res*. 2001; 2:102-12.

Ring AE, Ellis PA. Predictors of response to systemic therapy in breast cancer. Forum. 12(1): 19-32, 2002

Roses AD. Genome-based pharmacogenetics and the pharmaceutical industry. *Nat Rev Drug Discov*. 2002; 1:541-9.

TYKERB™ (lapatinib) Product Information. March, 2007.

14. APPENDICES

14.1. Appendix 1: Time and Events Table

Study Assessments	Pre-Study[a]	Day 1 (pre-dose)	Day 10 Until Resection	Post Study[b]
Informed Consent	X			
Physical Exam	X		X	X
Medical and Medication History	X			
Vital Signs	X	X	X	X
Clinical Labs[c]	X		X	X
12-lead ECG	X			
MUGA or ECHO[d]	X			
AE Monitoring and Concomitant Medications	Continuous			
Dosing[e]		X		
Tumor Biopsy / Resection[f]	X		X	
Pharmacokinetic Sampling[g]		X	X	
Pharmacodynamic Sampling[h]		X	X	X
Pharmacogenetic Sampling[i]		X		

a. All pre-study screening assessments should be scheduled within two weeks of the first dose of study drug. Screening assessments completed within 72 hours of first dose of study drug can be used as Day 1 assessments. Local lab ErbB2 results must be available prior to dosing on Day 1.
b. The follow-up visit should occur 7-28 days after the last dose of study medication or prior to initiating adjuvant therapy.
c. Refer to Appendix 2 for complete list of required clinical labs. Pregnancy testing is required for all women of child-bearing potential.
d. LVEF must be ≥50% by MUGA or ECHO to be eligible for enrollment in the study.
e. Study drug will be dosed daily from Day 1 until the day prior to the scheduled resection.
f. The archived tumor biopsy which is equivalent to the pre-dose tumor biopsy must be obtained prior to initiating study treatment. The post-treatment tumor resection must coincide with the previous day's lapatinib dose trough concentration and must be collected within 24 hours of last lapatinib dose.
g. Refer to Section 6.6 for pharmacokinetic sampling schedule.
h. Refer to Section 6.7 for pharmacodynamic sampling schedule.
i. Refer to Section 6.8 for pharmacogenetic sampling schedule.

14.2. Appendix 2: Clinical Laboratory Assessments

Hematology

2mL blood into ethylenediamine tetraacetic acid (EDTA) to screen for:

Hemoglobin (Hb)
Hematocrit (HCT)
Red blood cell count (RBC)
Mean Cell Volume (MCV)
Mean Cell Hemoglobin (MCHC)
Platelet count
White blood cell count (WBC)
Neutrophil count
Lymphocyte count
Monocyte count
Eosinophil count
Basophil count

Clinical Chemistry

4mL blood into SST to provide 2mL to screen for:

Sodium
Potassium
Chloride
Total CO_2
Calcium
Glucose (fasting)
Phosphorous (inorganic)
Protein (total)
Albumin
GGT
Bilirubin (total)
Alkaline Phosphatase
LDH
AST (SGOT)
ALT (SGPT)
Creatinine
Blood Urea Nitrogen
Uric Acid
Creatine Phosphokinase (CPK)

Other Chemistries

Serum β-hCG (all women)

14.3. Appendix 3: Country Specific Requirements

No country-specific requirements exist.

14.4. Appendix 4: NCI Performance Status Scale/Score, Karnofsky Performance Status Scale/Score

Able to carry on normal activity; no special care is needed	100	Normal; no complaints; no evidence of disease
	90	Able to carry on normal activity; minor signs or symptoms of disease
	80	Normal activity with effort; some signs or symptoms of disease
Unable to work; able to live at home and care for most personal needs; a varying amount of assistance is needed	70	Cares for self; unable to carry on normal activity or to do active work
	60	Requires occasional assistance but is able to care for most of his needs
	50	Requires considerable assistance and frequent medical care
Unable to care for self; requires equivalent of institutional or hospital care; disease may be progressing rapidly	40	Disabled; requires special care and assistance
	30	Severely disabled; hospitalization is indicated although death not imminent
	20	Very sick; hospitalization necessary; active supportive treatment is necessary
	10	Moribund; fatal processes progressing rapidly
	0	Dead

Consideration of the following information (in addition to information in the scale itself) in determining the KPS score will add consistency to the rating across centers and studies. Please try to obtain this information in a consistent manner in determining the KPS.

1. Weight loss or gain
2. Reduction in energy, increase in fatigue
3. Difficulty in bathing or grooming
4. Difficulty in walking or moving around
5. Difficulty in driving
6. Difficulty working full or part time

14.5. Appendix 5: New York Heart Association Functional Classification

I	No symptoms and no limitation in ordinary physical activity.
II	Mild symptoms and slight limitation during ordinary activity.<br><br>Comfortable at rest.
III	Marked limitation in activity due to symptoms, even during less-than-ordinary activity.<br><br>Comfortable only at rest.
IV	Severe limitations. Experiences symptoms even while at rest.

14.6. Appendix 6: Amendment 01 Summary of Changes

Amendment 01 includes the following changes:

Protocol Summary, Rationale, 1st paragraph

Added text:

Although the efficacy of treatment with GW572016 in the neoadjuvant breast cancer setting is unknown at this time, tumor biomarker data generated from this study will guide future Phase II/III breast cancer efficacy trial design.

Protocol Summary, Rationale, 3rd paragraph

Original text:

This study will examine tumor tissue and normal tissue obtained adjacent to the tumor in treatment-naïve breast cancer patients to determine if inhibition of EGFR and ErbB2 phosphorylation and downstream mediators of tumor cell growth and survival (e.g., phosphorylated-ERK1/2, phosphorylated AKT and cyclin D1 protein and potentially other downstream markers of kinase activity) is enhanced by dosing GW572016 twice daily as compared to once daily. Although the plasma half-life of GW572016 is approximately 14 hours, thus supporting BID dosing, pre-clinical data suggests that GW572016 may accumulate in tumor tissue making BID dosing unnecessary. Patients will be randomized to one of two dose groups of GW572016 and will receive treatment for 14 to 21 days. Biopsy tissue obtained pre-treatment (Day –10 to Day 0) will be compared to tissue obtained by biopsy or resection post treatment (Day 14 to Day 21). In patients who undergo resection following 14 to 21 days, if sufficient tumor tissue is available, transcriptome analysis using microarray will be performed to examine the molecular profile of tumor tissue for factors which may be predictive of response to GW572016 treatment. A minimum of 14 days of treatment is required for GW572016 to reach steady state and provide meaningful protein biomarker results, therefore; in the event that the tumor is resected prior to completing 14 days of GW572016 treatment, only transcriptome analysis of resected tumor tissue will be examined.

Changed to read:

This study will examine the inhibition of EGFR and ErbB2 phosphorylation and downstream mediators of tumor cell growth and survival (e.g., phosphorylated ERK1/2, phosphorylated AKT and cyclin D1 protein and potentially other downstream markers of kinase activity) tumor tissue in treatment-naïve breast cancer patients for three dosing schedules of GW572016. Patients will be randomized to one of the three dose groups of GW572016 and will receive treatment for 9 to 13 days prior to surgical resection of the tumor. Biopsy tissue obtained pre-treatment (Day –3 to Day 0) will be compared to tissue obtained by resection post-treatment (Day 10 to Day 14). In addition, blood and tumor obtained at the time of resection will be examined for steady state, trough plasma and intratumoral drug concentrations of GW572016.

Protocol Summary, Rationale, 4th paragraph

Deleted text:

Following completion of the randomized portion of the study, 10 to 20 additional patients, will be enrolled at a dose of GW572016 250 mg once daily to examine the inhibition of EGFR and ErbB2 phosphorylation and downstream biomarkers in tumor tissue and normal breast tissue obtained adjacent to the tumor. EGFR has been shown to play an important role in normal ductal epithelium as the major controller of proliferation. The EGFR signaling networks in normal tissue may respond to treatment with GW572016 differently than the aberrant signaling pathways driving tumor cell growth and proliferation. This data will be examined to investigate the potential for low dose GW572016 to be used as a cancer prevention treatment in patients with a high risk for developing breast cancer.

Protocol Summary, Objectives

Original text:

Primary

To investigate the effects of once daily versus twice daily dosing schedules of GW572016 on intracellular mediators that regulate tumor cell growth and survival (e.g., inhibition of phosphorylated forms of ERK1/2, AKT, cyclin D1 or potentially other downstream markers of kinase activity) comparing pre-treatment and post-treatment tumor and normal breast tissue samples.

To assess the safety and tolerability of GW572016 at multiple doses when administered to patients with treatment-naïve T2-T4 breast tumors.

Secondary

To describe the pharmacokinetics and pharmacodynamics of GW572016 at doses and dosing schedules used in this study.

To perform transcriptome analysis using microarray to examine the molecular profile of tumors for additional factors that may influence biological and therefore clinical responses to GW572016 (e.g. tumor expression of EGF receptor ligands, insulin-like growth factor receptor 1, Ras mutations and potentially other molecular markers that may be predictive of response to treatment with GW572016).

To investigate the effects of low dose GW572016 treatment on intracellular mediators of cell growth in tumor and normal breast tissue (e.g., inhibition of phosphorylated forms of ERK1/2, AKT, cyclin D1 or potentially other downstream markers) comparing pre-treatment and post-treatment tissue samples.

To assess the effects of GW572016 therapy on the proteomic profile and circulating extracellular domain (ECD) of EGFR and ErbB2 in peripheral blood.

Pharmacogenetics may be investigated if at any time it appears that there is a potential unexpected or unexplained variation in GW572016 response or handling (e.g., safety and/or clinical activity) in this clinical study or in a series of clinical studies using GW572016.

Changed to read:

Primary

To investigate the effects of three dosing schedules of GW572016 on intracellular mediators that regulate tumor cell growth and survival (e.g., inhibition of phosphorylated forms of ERK1/2, AKT, cyclin D1 or potentially other downstream markers of kinase activity) by comparing pre-treatment and post-treatment breast tumor tissue samples.

Secondary

To assess the safety and tolerability of GW572016 at multiple dosing schedules when administered to patients with treatment-naïve breast tumors.

To examine the steady state, trough plasma and intratumoral concentrations of GW572016 obtained at the time of tumor resection.

To investigate the effects of GW572016 therapy on the proteomic profile and circulating extracellular domain (ECD) of EGFR and ErbB2 in peripheral blood.

Pharmacogenetics may be investigated if at any time it appears that there is a potential unexpected or unexplained variation in GW572016 response or handling (e.g., safety and/or clinical activity) in this clinical study or in a series of clinical studies using GW572016.

Protocol Summary, Endpoints

Original text:

Primary

Comparison of the effects of once daily versus twice daily dosing schedules of GW572016 on protein biomarkers involved in regulating tumor cell proliferation and survival (e.g., phosphorylated-ERK1/2, phosphorylated AKT, cyclin D1 protein and potentially other downstream markers of kinase activity) in pre-treatment and post-treatment tumor and normal breast tissue samples.

Evaluation of adverse events (AEs) and changes in laboratory values from pre-dose, during dosing and post-dose values.

Secondary

Calculation of GW572016 pharmacokinetic parameters such as AUCτ, C_{max} , and t_{max}, at Day 1 and at steady state (Day 14 – 19) in once daily and twice daily GW572016 dosing regimens used in this study.

Characterization of the molecular profile of tumors by transcriptome analyis using microarry for factors that may influence biological response and therefore clinical response to GW572016 (e.g., tumor expression of EGF receptor ligands, insulin-like growth factor 1, Ras mutations and transforming growth factor α).

Evaluation of the effects of low dose treatment with GW572016 on protein biomarkers regulating cell proliferation in tumor and normal breast tissue (e.g., phosphorylated-ERK1/2, phosphorylated AKT, cyclin D1 protein and potentially other downstream markers of kinase activity) in pre-treatment and post-treatment tissue samples..

- Assessment of peripheral blood to examine the proteomic profile and determination of serum EGFR and ErbB2 (circulating extracellular domain) levels in response to GW572016.

Changed to read:

Primary

Comparison of the effects of three dosing schedules of GW572016 on protein biomarkers involved in regulating tumor cell proliferation and survival (e.g., phosphorylated-ERK1/2, phosphorylated AKT, cyclin D1 protein and potentially other downstream markers of kinase activity) in pre-treatment and post-treatment breast tumor tissue samples.

Secondary

Evaluation of adverse events (AEs) and changes in laboratory values from pre-dose, and post-dose values.

Examination of plasma and intratumoral GW572016 steady state trough concentrations.

Assessment of peripheral blood to examine the proteomic profile and determination of serum EGFR and ErbB2 (circulating extracellular domain) levels in response to GW572016.

Protocol Summary, Study Design

Original text:

This will be a two-part study consisting of a randomized (Part 1) and non-randomized (Part 2) design. In Part 1 of the study, patients will be randomized to one of two dose groups of GW572016, administered in a parallel design. The doses to be administered include 1000 mg dosed once daily and 500 mg dosed twice daily (every 12 hours). Following completion of Part 1, 10 to 20 additional patients will be enrolled to examine biomarker changes in tumor and normal breast tissue obtained pre- and post-treatment using low dose (250 mg) GW572016.

Prior to entering the study, the EGFR and/or ErbB2 status will be determined for each patient from a recent biopsy. Only patients with treatment-naïve, T2-T4 breast tumors, which overexpress EGFR by IHC and/or ErbB2 by IHC or fluorescence in situ hybridization (FISH), or that express activated phosphorylated EGFR and/or ErbB2 determined by semi-quantitative IHC will be eligible for this study. For eligible patients, tumor tissue and normal breast tissue obtained by biopsy adjacent to the tumor will be examined for biomarkers involved in regulating tumor cell proliferation and survival (e.g., ErbB2, ERK1/2, phosphorylated-ERK1/2, AKT, phosphorylated AKT and cyclin D1 protein and potentially other downstream markers of kinase activity). Patients will receive treatment with GW572016 for 14 to 21 days (a minimum of 14 days of GW572016 treatment is required for the patient to be considered evaluable). In patients who undergo resection following 14 to 21 days, if sufficient tumor tissues is available, transcriptome analysis using microarray will be performed to examine the molecular profile of tumor tissue for factors which may be predictive of response to GW572016 treatment. In patients for whom surgical resection is performed prior to reaching steady state drug concentrations (e.g. prior to 14 days of treatment), tumor tissue will only be examined for transcriptome analysis using microarray.

Patients administered GW572016 once daily will have their post-treatment resection or biopsy performed approximately 24 hours following the previous day’s dose while those administered GW572016 twice daily will have their resection or biopsy performed approximately 12 hours following the previous day’s second dose to temporally coincide with the 24 hour steady state trough concentrations. A sufficient number of patients will be enrolled to ensure there are a minimum of 20 evaluable patients in each randomized dose group. Approximately 10 to 20 patients will be enrolled in the non-randomized low-dose treatment group.

Serial blood samples for pharmacokinetic analysis of GW572016 plasma concentrations will be obtained during the dosing interval on Day 1 and prior to the scheduled tumor resection or biopsy on Day 14-21. So as not to interfere with pre-operative surgical preparations in patients undergoing resection, pharmacokinetic sampling will occur two days prior to the scheduled surgery (i.e., if tumor resection is scheduled for Day 21, pharmacokinetic sampling should occur on Day 19). Safety (AEs, clinical laboratory values) will be assessed throughout the study.

Changed to read:

This will be a randomized, open-label study. Patients will be randomized to one of three dose groups of GW572016, administered in a parallel design. The doses to be administered include 1500 mg and 1000 mg dosed once daily and 500 mg dosed twice daily (every 12 hours).

Prior to entering the study, the EGFR and/or ErbB2 status will be determined for each patient from a recent biopsy. Only patients with treatment-naïve, breast tumors measuring greater than 2 cm, which overexpress EGFR by IHC and/or ErbB2 by IHC or fluorescence in situ hybridization (FISH), or which express activated phosphorylated EGFR and/or ErbB2 determined by semi-quantitative IHC will be eligible for this study. For eligible patients, an additional biopsy will be obtained pre-treatment and tumor tissue will be examined by semi-quantitative IHC for biomarkers involved in regulating tumor cell proliferation and survival (e.g., ErbB2, ERK1/2, phosphorylated-ERK1/2, AKT, phosphorylated AKT and cyclin D1 protein and potentially other downstream markers of kinase activity). Patients will receive treatment with GW572016 for 9 to 13 days in the interim period between initial diagnosis and scheduled surgical resection. Tumor tissue obtained at resection will be examined for biomarkers as described above and compared to the results from the pre-treatment tumor sample. In addition, blood and tumor will be obtained at the time of resection and will be examined for steady state, trough concentrations of GW572016.

Patients administered GW572016 once daily will have their post-treatment surgical resection performed approximately 24 hours (± 3 hours) following the previous day's dose while those administered GW572016 twice daily will have their resection or biopsy performed approximately 12 hours (± 3 hours) following the previous day's second dose to temporally coincide with the 24 hour steady state trough concentrations. Safety (AEs, clinical laboratory values) will be assessed throughout the study.

Protocol Summary, Study Population, 1st paragraph

Original text:

Approximately 80 patients will be enrolled at 3-4 study centers. Male or female patients, aged 18 years or older with T2-T4 breast tumors which overexpress EGFR and/or ErbB2 will be enrolled. Patients must be naïve to cytotoxic, radiation, hormonal and biologic therapy to treat their breast cancer.

Changed to read:

A sufficient number of patients will be enrolled to obtain 60 evaluable patients (20 per dose group) at 5-6 study centers. Male or female patients, aged 18 years or older with a breast tumor measuring at least 2 cm with evidence of EGFR and/or ErbB2 overexpression will be enrolled. Patients must be naïve to cytotoxic, radiation, hormonal and biologic therapy to treat breast cancer.

Protocol Summary, Study Assessments and Procedures

Original text:

Screening assessments will be completed within 14 days prior to the first dose of investigational product. Screening assessments performed within 72 hours of the first treatment period may also be used as Day 1 assessments. After obtaining informed consent, screening assessments will be completed to determine the patient's eligibility for enrollment.

Pre-dose tumor biopsy samples may be obtained up to ten days prior to initiating dosing with GW572016. Three core needle biopsies will be required (two tumor needle biopsies and one normal breast tissue core needle biopsy obtained adjacent to the tumor).

Patients will receive study medication for 14 to 21 days (or until the day prior to surgical resection or biopsy of their tumor). Post-treatment tumor resection or biopsy will coincide with the GW572016 trough concentration. If post-treatment tissue is obtained by biopsy, three core needle biopsies will be required as described above. If tumor tissue is obtained by resection, a minimum of 250 mg of tumor and normal tissue is required for biomarker analysis.

Blood samples (2 x 5 ml tubes) will be obtained on Day 1 and the on the same day as pharmacokinetic sampling post-treatment to evaluate changes in circulating/secreted proteins (proteomic analyis) and biomarkers (circulating extracellular domain (ECD) concentrations of EGFR and ErbB2) that may correlate with biological response at the tumor site and therefore provide an easily obtainable surrogate marker for determining therapeutic benefit of GW572016.

Changed to read:

After obtaining informed consent, screening assessments will be completed to determine the patient's eligibility for enrollment. Screening assessments must be completed within 14 days prior to the first dose of investigational product. Screening assessments performed within 72 hours of the first treatment period may also be used as Day 1 assessments. The pre-treatment tumor biopsy sample may be obtained up to three days prior to initiating dosing with GW572016. Three core needle biopsies will be required.

Patients will receive study medication for 9 to13 days (until the day prior to scheduled surgical resection of the tumor). Surgical resection will coincide with the GW572016 trough concentration. A minimum of 250 mg of tumor tissue is required for biomarker and GW572016 intratumoral concentration analysis. A 2 mL blood sample will be obtained at the time of tumor resection to examine steady state, trough plasma GW572016 concentrations. Safety (AEs, clinical laboratory values) will be assessed throughout the study.

Blood samples (2 x 5 ml tubes) will be obtained on Day 1, on the same day as the tumor resection and at the post-treatment safety visit to evaluate changes in circulating/secreted proteins (proteomic analysis) and biomarkers (circulating extracellular domain [ECD] concentrations of EGFR and ErbB2) that may correlate with biological response at the

tumor site and therefore provide an easily obtainable surrogate marker for determining therapeutic benefit of GW572016.

Protocol Summary, Investigational Product

Original text:

Patients will receive oral tablets of GW572016 ditosylate salt. GW572016 will be supplied to the study site as 250 mg tablets. The number of tablets supplied to the patient at each visit will be dependent on the dose group assignment.

Changed to read:

Patients will receive oral tablets of GW572016 ditosylate salt. GW572016 will be supplied to the study site as 250 mg tablets. The number of tablets supplied to the patient will be dependent on the dose group assignment and the period of time between enrollment and scheduled resection.

Section 1.4 Preliminary Results from Healthy Volunteers and Patients, 6th and 7th paragraph

Original text:

The first study in cancer patients (EGF10003) is ongoing. Daily doses of up to 1800 mg have been administered. A maximum tolerated dose (MTD) was not achieved following once daily dosing. Only Grade 1 and Grade 2 toxicity (preliminary data) have been seen following daily dosing. Those AEs which have occurred most frequently include diarrhea, fatigue, rash (including acniform rash) and nausea. Preliminary pharmacokinetic data from EGF10003 indicate that serum concentrations increase in a generally proportional manner with increasing dose up to 1800 mg. In this study 6 patients have received 900 mg twice daily and 8 patients have received 750mg twice daily. There has been an increased incidence of gastrointestinal (GI) intolerance (nausea, vomiting and diarrhea) in patients receiving twice daily dosing. This may be due to a local GI effect or may be due to enhanced absorption and increased bioavailability. Pharmacokinetic data from twice daily dosing portion of the study is in progress.

The second study in cancer patients (EGF10004) is also in progress. Preliminary AE data in this study is similar to that seen in EGF10003. AEs have been Grade 1 or 2 and primarily consist of diarrhea, vomiting, nausea and rash.

Changed to read:

The first study in cancer patients (EGF10003) is ongoing. Daily doses of up to 1800 mg have been administered. A maximum tolerated dose (MTD) was not achieved following once daily dosing. Only Grade 1 and Grade 2 toxicity (preliminary data) have been seen following daily dosing. Those AEs which have occurred most frequently include diarrhea (18-25%), rash (<10%) and nausea (< 10%). Preliminary pharmacokinetic data from EGF10003 indicate that serum concentrations increase in a generally proportional

manner with increasing dose up to 1800 mg. In this study 6 patients have received 900 mg twice daily and 8 patients have received 750mg twice daily. There has been an increased incidence of gastrointestinal (GI) intolerance (nausea, vomiting and diarrhea) to approximately 34% in patients receiving twice daily dosing. This may be due to a local GI effect or may be due to enhanced absorption and increased bioavailability.

The second study in cancer patients (EGF10004) is also in progress. Preliminary AE data in this study is similar to that seen in EGF10003. AEs have been primarily Grade 1 or 2 and consist of diarrhea (30%) and rash (12%). There was one report of Grade 3 reflux which was considered related to a large pill burden. In general, the time to AE onset was 2-4 weeks following initiation of daily dosing.

Section 1.5 Rationale, 1st parapharph

Added text:

Although the efficacy of treatment with GW572016 in the neoadjuvant breast cancer setting is unknown at this time, tumor biomarker data generated from this study will guide future Phase II/III breast cancer efficacy trial design.

Section 1.5 Rationale, 3rd paragraph

Original text:

This study will examine tumor tissue and normal tissue obtained adjacent to the tumor in treatment-naïve breast cancer patients to determine if inhibition of EGFR and ErbB2 phosphorylation and downstream mediators of tumor cell growth and survival (e.g., phosphorylated-ERK1/2, phosphorylated AKT and cyclin D1 protein and potentially other downstream markers of kinase activity) is enhanced by dosing GW572016 twice daily as compared to once daily. Although the plasma half-life of GW572016 is approximately 14 hours, thus supporting BID dosing, pre-clinical data suggests that GW572016 may accumulate in tumor tissue making BID dosing unnecessary. Patients will be randomized to one of two dose groups of GW572016 and will receive treatment for 14 to 21 days. Biopsy tissue obtained pre-treatment (Day –10 to Day 0) will be compared to tissue obtained by biopsy or resection post treatment (Day 14 to Day 21). In patients who undergo resection following 14 to 21 days, if sufficient tumor tissue is available, transcriptome analysis using microarray will be performed to examine the molecular profile of tumor tissue for factors which may be predictive of response to GW572016 treatment. A minimum of 14 days of treatment is required for GW572016 to reach steady state and provide meaningful protein biomarker results, therefore; in the event that the tumor is resected prior to completing 14 days of GW572016 treatment, only transcriptome analysis of resected tumor tissue will be examined.

Changed to read:

This study will examine the inhibition of EGFR and ErbB2 phosphorylation and downstream mediators of tumor cell growth and survival (e.g., phosphorylated ERK1/2, phosphorylated AKT and cyclin D1 protein and potentially other downstream markers of kinase activity) tumor tissue in treatment-naïve breast cancer patients for three dosing schedules of GW572016. Patients will be randomized to one of the three dose groups of GW572016 and will receive treatment for 9 to 13 days during the interim period between diagnosis and surgical resection of the tumor. Biopsy tissue obtained pre-treatment (Day –3 to Day 0) will be compared to tissue obtained by resection post-treatment (Day 10 to Day 14). In addition, blood and tumor obtained at the time of resection will be examined for steady state, trough plasma and intratumoral drug concentrations of GW572016.

Section 1.5 Rationale, 4th paragraph

Deleted text:

Following completion of the randomized portion of the study, 10 to 20 additional patients, will be enrolled at a dose of GW572016 250 mg once daily to examine the inhibition of EGFR and ErbB2 phosphorylation and downstream biomarkers in tumor tissue and normal breast tissue obtained adjacent to the tumor. EGFR has been show to play an important role in normal ductal epithelium as the major controller of proliferation. The EGFR signaling networks in normal tissue may respond to treatment with GW572016 differently than the aberrant signaling pathways driving tumor cell growth and proliferation. This data will be examined to investigate the potential for low dose GW572016 to be used as a cancer prevention treatment in patients with a high risk for developing breast cancer.

Section 2.0 Objectives

Original text:

Primary

To investigate the effects of once daily versus twice daily dosing schedules of GW572016 on intracellular mediators that regulate tumor cell growth and survival (e.g., inhibition of phosphorylated forms of ERK1/2, AKT, cyclin D1 and potentially other downstream biomarkers) in pre-treatment and post-treatment tumor and normal breast tissue samples.

To assess the safety and tolerability of GW572016 at multiple doses when administered to patients with treatment-naïve T2-T4 breast tumors.

Secondary

To examine the pharmacokinetics and pharmacodynamics of GW572016 at doses and dosing schedules used in this study.

To perform transcriptome analysis using microarry to examine the molecular profile of tumors for additional factors that may influence biological and therefore clinical responses to GW572016 (e.g. tumor expression of EGF receptor ligands, insulin-like growth factor receptor 1, Ras mutations).

To investigate the effects of low dose GW572016 treatment on intracellular mediators of cell growth in tumor and normal breast tissue (e.g., inhibition of phosphorylated forms of ERK1/2, AKT, cyclin D1 and potentially other downstream biomarkers of kinase activity) comparing pre-treatment and post-treatment tissue samples.

To assess the effects of GW572016 therapy on the proteomic profile and circulating extracellular domain (ECD) of EGFR and ErbB2 in peripheral blood.

Pharmacogenetics may be investigated if at any time it appears that there is a potential unexpected or unexplained variation in GW572016 response or handling (e.g., safety and/or clinical activity) in this clinical study or in a series of clinical studies using GW572016.

Section 3.0 Endpoints

Original text:

Primary

Comparison of the effects of once daily versus twice daily dosing schedules of GW572016 on biomarkers involved in regulating tumor cell proliferation and survival (e.g., phosphorylated-ERK1/2, phosphorylated AKT, cyclin D1 protein and potentially other downstream markers of kinase activity) in pre-treatment and post-treatment tumor and normal breast tissue samples.

Evaluation of adverse events (AEs) and changes in laboratory values from pre-dose, during dosing and post-dose values.

Secondary

Calculation of GW572016 pharmacokinetic parameters such as AUC, C_{max}, and t_{max}, on Day 1 and at steady state (Day 14 – 19) in once daily and twice daily GW572016 dosing regimens used in this study.

Characterization of the molecular profile of tumors by transcriptome analysis using microarray for factors that may influence biological response and therefore clinical response to GW572016 (e.g., tumor expression of EGF receptor ligands, insulin-like growth factor 1, Ras mutations and transforming growth factor α).

Evaluation of the effect of low dose treatment with GW572016 on protein biomarkers responsible for cell proliferation in tumor and normal breast tissue (e.g., phosphorylated ERK1/2, phosphorylated AKT, cyclin D1 protein and potentially other downstream biomarkers of kinase activity) comparing pre-treatment and post-treatment tissue samples.

Assessment of peripheral blood to examine the proteomic profile and determination of serum EGFR and ErbB2 levels (circulating extracellular domain) in response to GW572016.

Changed to read:

Primary

Comparison of the effects of three dosing schedules of GW572016 on biomarkers involved in regulating tumor cell proliferation and survival (e.g., phosphorylated-ERK1/2, phosphorylated AKT, cyclin D1 protein and potentially other downstream markers of kinase activity) in pre-treatment and post-treatment breast tumor tissue samples.

Secondary

Evaluation of adverse events (AEs) and changes in laboratory values from pre-dose, and post-dose values.

Examination of plasma and intratumoral GW572016 steady state trough concentrations.

Assessment of peripheral blood to examine the proteomic profile and determination of serum EGFR and ErbB2 (circulating extracellular domain) levels in response to GW572016.

Section 4.0 Study Design

Original text:

This will be a two-part study consisting of a randomized (Part 1) and non-randomized (Part 2) design. In Part 1 of the study, patients will be randomized to one of two dose groups of GW572016, administered in a parallel design as shown in Table 1 below. The doses to be administered include 1000 mg dosed once daily and 500 mg dosed twice daily (every 12 hours). Following completion of Part 1, 10 to 20 additional patients will be enrolled to examine biomarker changes in tumor and normal breast tissue obtained pre- and post-treatment using low dose (250 mg) GW572016.

Prior to entering the study, the EGFR and/or ErbB2 status will be determined for each patient from a recent biopsy. Only patients with treatment-naïve, T2-T4 breast tumors, which over-express EGFR by IHC and/or ErbB2 by IHC or fluorescence in situ hybridization (FISH), or that express activated phosphorylated EGFR and/or ErbB2 determined by semi-quantitative IHC will be eligible for this study. For eligible patients,

tumor tissue and normal breast tissue obtained by biopsy adjacent to the tumor will be examined for biomarkers involved in regulating tumor cell proliferation and survival (e.g., ErbB2, ERK1/2, phosphorylated-ERK1/2, AKT, phosphorylated AKT and cyclin D1 protein and potentially other downstream markers of kinase activity). Patients will receive treatment with GW572016 for 14 to 21 days (a minimum of 14 days of GW572016 treatment is required for the patient to be considered evaluable). In patients who undergo resection following 14 to 21 days, if sufficient tumor tissues is available, transcriptome analysis using microarray will be performed to examine the molecular profile of tumor tissue for factors which may be predictive of response to GW572016 treatment. In patients for whom surgical resection is performed prior to reaching steady state drug concentrations (e.g. prior to 14 days of treatment), tumor tissue will only be examined for transcriptome analysis using microarray.

Patients administered GW572016 once daily will have their post-treatment resection or biopsy performed approximately 24 hours following the previous day's dose while those administered GW572016 twice daily will have their resection or biopsy performed approximately 12 hours following the previous day's second dose to temporally coincide with the 24 hour steady state trough concentrations. A sufficient number of patients will be enrolled to ensure there are a minimum of 20 evaluable patients in each randomized dose group. Approximately 10 to 20 patients will be enrolled in the non-randomized low-dose treatment group.

Serial blood samples for pharmacokinetic analysis of GW572016 plasma concentrations will be obtained during the dosing interval on Day 1 and prior to the scheduled tumor resection or biopsy on Day 14-21. So as not to interfere with pre-operative surgical preparations in patients undergoing resection, pharmacokinetic sampling will occur two days prior to the scheduled surgery (i.e., if tumor resection is scheduled for Day 21, pharmacokinetic sampling should occur on Day 19). Safety (AEs, clinical laboratory values) will be assessed throughout the study.

Information regarding the biological effectiveness, pharmacokinetics, safety and tolerance obtained from this study will be used to determine the dose and dosing regimens to be used in future phase II/III efficacy trials.

Table 1 GW572016 Randomized Treatment Assignment

Randomization Group	A	B
Dose / Dosing Schedule	1000 mg QD	500 mg BID

Changed to read:

This will be a randomized, open-label study. Patients will be randomized to one of three dose groups of GW572016, administered in a parallel design as shown in Table 1 below. The doses to be administered include 1500 mg and 1000 mg dosed once daily and 500 mg dosed twice daily (every 12 hours).

Prior to entering the study, the EGFR and/or ErbB2 status will be determined for each patient from a recent biopsy. Only patients with treatment-naïve, breast tumors measuring greater than 2 cm, which overexpress EGFR by IHC and/or ErbB2 by IHC or fluorescence in situ hybridization (FISH), or which express activated phosphorylated EGFR and/or ErbB2 determined by semi-quantitative IHC will be eligible for this study. For eligible patients, an additional biopsy will be obtained pre-treatment and tumor tissue will be examined by semi-quantitative IHC for biomarkers involved in regulating tumor cell proliferation and survival (e.g., ErbB2, ERK1/2, phosphorylated-ERK1/2, AKT, phosphorylated AKT and cyclin D1 protein and potentially other downstream markers of kinase activity). Patients will receive treatment with GW572016 for 9 to 13 days in the interim period between initial diagnosis and scheduled surgical resection. Tumor tissue obtained at resection will be examined for biomarkers as described above and compared to the results from the pre-treatment tumor sample. In addition, blood and tumor will be obtained at the time of resection and will be examined for steady state, trough concentrations of GW572016.

Patients administered GW572016 once daily will have their post-treatment surgical resection performed approximately 24 hours (± 3 hours) following the previous day's dose while those administered GW572016 twice daily will have their resection performed approximately 12 hours (± 3 hours) following the previous day's second dose to temporally coincide with the 24 hour steady state trough concentrations. Safety (AEs, clinical laboratory values) will be assessed throughout the study.

Table 1 GW572016 Randomized Treatment Assignment

Randomization Group	A	B	C
Dose / Dosing Schedule	1500 mg QD	1000 mg QD	500 mg BID

Section 5.1 Number of Subjects

Original text:

Approximately 80 patients will be enrolled at 3-4 study centers.

Changed to read:

A sufficient number of patients will be enrolled to obtain 60 evaluable patients (20 per dose group) at 5-6 study centers.

Section 5.2.1 Inclusion Criteria, #1

Original text:

1. Has a histologically confirmed, treatment-naïve, T2-T4 breast tumor that can be readily biopsied and that EITHER (i) over-expresses total EGFR by IHC, ErbB2 by IHC and/or FISH gene amplification, (ii) or express activated, phosphorylated EGFR or ErbB2.

Changed to read:

1. Has a histologically confirmed, treatment-naïve, breast tumor measuring at least 2 cm that can be readily biopsied and that EITHER (i) over-expresses total EGFR by IHC, ErbB2 by IHC and/or FISH gene amplification, (ii) or expresses activated, phosphorylated EGFR or ErbB2.

Section 5.2.2 Exclusion Criteria, # 16

Original text:

Poor venous access.

Changed to read:

In the clinical judgement of the investigator or designated staff the patient has inadequate venous access for the protocol required blood draws.

Section 6.0 Study Assessments and Procedures

Original text:

The study specific assessments and procedures are detailed below and are outlined in the Time and Events Schedule contained in Section 14.1., Appendix 1. The total amount of blood to be obtained during this study is approximately 150 mL.

Changed to read:

The study specific assessments and procedures are detailed below and are outlined in the Time and Events Schedule contained in Section 14.1., Appendix 1. The total amount of blood to be obtained during this study is approximately 60 mL.

Section 6.3 On Study Assessments

Original text:

On Day 1 and post-treatment (Day 14 to 21) patients will have safety, pharmacokinetic and pharmacodynamic assessments performed. The following procedures will be completed.

Full physical examination, including semi-recumbent vital signs (blood pressure and heart rate) to occur at Day 1 (if greater than 72 hours since screening physical exam) and two days prior to scheduled resection.

Clinical Chemistry on Day 1 and prior to scheduled resection or biopsy on Day 14-21.

Hematology on Day 1 and prior to scheduled resection or biopsy on Day 14-21.

Assessment of study medication compliance.

Assessment of adverse events.

Tumor surgical resection or biopsy (Day 14-21) to coincide with previous day's dose GW572016 trough concentration.

Pharmacokinetic sampling on Day 1 and prior to scheduled resection or biopsy on Day 14 to 21. Refer to Section 6.6 for sampling schedule.

Pharmacodynamic sampling (2 x 5 mL tubes of whole blood) on Day 1 and the on the same day as pharmacokinetic sampling.. Refer to Section 6.7 for sampling schedule

Pharmacogenetic sampling (1 x 10 mL tube of whole blood) on Day 1 or at any time during the conduct of the study. Refer to Section 6.8 for sampling schedule.

Changed to read:

On Day 0 or 1 and on the day prior to surgical resection (Day 9 to 13) patients will have safety, pharmacokinetic and pharmacodynamic assessments performed. The following procedures will be completed and time and date of each assessment will be documented in the source records and the CRF.

Full physical examination, including vital signs (blood pressure and heart rate) to occur at Day 0 or 1 (if greater than 72 hours since screening physical exam) and prior to scheduled surgical resection (Day 9 to13).

Clinical Chemistry on Day 0 or 1 and prior to scheduled surgical resection on Day 9 to 13.

Hematology on Day 0 or 1 and prior to scheduled surgical resection on Day 9 to 13.

Assessment of study medication compliance.

Assessment of adverse events.

Tumor surgical resection (Day 10 to 14) to coincide with previous day's dose GW572016 trough concentration.

A blood sample for pharmacokinetic analysis will be collected pre-dose on Day 0 or 1 and at the time of surgical resection on Day 10 to 14. Refer to Section 6.6 for sampling schedule.

Pharmacodynamic sampling (2 x 5 mL tubes of whole blood) on Day 0 or 1 and the on the day prior to scheduled resection (Day 9 to 13). Refer to Section 6.7 for sampling schedule.

Pharmacogenetic sampling (1 x 10 mL tube of whole blood) on Day 1 or at any time during the conduct of the study. Refer to Section 6.8 for sampling schedule.

Section 6.5.4 Adverse Events

Original text:

Patients will be monitored from screening until 28 days after the last dose for the occurrence of AEs.

Changed to read:

Patients will be monitored from screening until 28 days after the last dose (or until initiation of adjuvant chemotherapy) for the occurrence of AEs.

Section 6.5.5 Criteria for Study Hold

Added text:

If Grade III or IV toxicity is observed in ≥ 30% (≥ 3 patients) of the first 10 patients in each dosing group, the study will placed on hold (ie. no further accrual). The safety data will be reviewed jointly by the GSK medical monitor and the Principal Investigators to determine if termination of additional enrollment in the given GW572016 dosing group is warranted.

Section 6.6 Pharmacokinetics

Original text:

Patients will have a cannula inserted prior to beginning pharmacokinetic sampling. The cannula will be kept patent by means of a saline lock. The total amount of blood to be collected in the pharmacokinetic portion of the study is approximately 60 mL over a 2-3 week period.

Prior to collection, the collection tube and plasma storage tube must be labeled with the corresponding bar-coded labels provided by GSK. The sample labels will include Protocol Number, Analyte, Subject Number, Planned Sample Time and Dosing Day. The labels must be placed along the length of the tube so the bar code can be easily read. So that the tubes will fit into the autoanalyzer test tube rack, tape must not be used to secure the labels.

Each 2 mL blood sample will be drawn into a vacutainer containing K3-EDTA and centrifuged to separate the plasma. The plasma (approximately 1 mL) will then be transferred into a separate storage tube for GW572016 analysis. Until assayed, the samples must be kept frozen in a freezer set at or below -20°C. All samples must be shipped with dry ice.

Patients receiving GW572016 once daily will have samples drawn on Day 1 and prior to scheduled post-treatment resection or biopsy at the following timepoints: predose (within 60 minutes of study drug administration), 0.25, 0.5, 1, 2, 3, 4, 6, 8, 10, 12, and 24 hours post dose. So as not to interfere with pre-operative surgical preparations in patients undergoing resection, pharmacokinetic sampling will occur two days prior to the scheduled surgery (i.e., if tumor resection is scheduled for Day 21, pharmacokinetic sampling should occur on Day 19).

Patients receiving GW572016 twice daily (doses separated by 12 hours) will have samples drawn around the first dose on Day 1 and two days prior to scheduled resection at the following timepoints: predose (within 60 minutes of study drug administration), 0.25, 0.5, 1, 2, 3, 4, 6, 8, 10, and 12 hours post dose.

Changed to read:

A 2 mL blood sample for pharmacokinetic analyis will be collected predose (Day 0 or 1) and as close as possible to the time of surgical resection (Day 10 to 14) to examine the steady state, trough plasma GW572016 concentration.

Section 6.7 Pharmacodynamics

Original text:

Blood samples (2 x 5 mL tubes) will be obtained on Day 1 and the on the same day as pharmacokinetic sampling post-treatment to evaluate changes in circulating/secreted proteins (proteomic analyis) and biomarkers (circulating extracellular domain [ECD] concentrations of EGFR and ErbB2) that may correlate with biological response at the tumor site and therefore provide an easily obtainable surrogate marker for determining therapeutic benefit of GW572016. An additional 5 mL of blood will be obtained at the 28 Day post-treatment study visit to examine ECD concentrations of EGFR and ErbB2.

The relative abundences of mRNAs for approximately 20,000 genes will be assessed by DNA microarray (methods on file in Genomic and Proteomic Scinences, GSK). Protoemic analysis will be carried out by a method to be determined (e.g., high resolution 2-D gel electrophoresis, Selective Lasar Desorption Ionization Mass Spectometry).

Changed to read:

Blood samples (2 x 5 mL tubes) will be obtained pre-treatment (Day 0 or 1) and on the day prior to surgical resection (Day 9 to 13) to evaluate changes in circulating/secreted proteins (proteomic analyis) and biomarkers (circulating extracellular domain [ECD] concentrations of EGFR and ErbB2) that may correlate with biological response at the tumor site and therefore provide an easily obtainable surrogate marker for determining

therapeutic benefit of GW572016. An additional 5 mL of blood will be obtained at the Post Study Visit to examine ECD concentrations of EGFR and ErbB2.

Section 6.7.1 Tumor Tissue Samples (Previous title Tumor and Normal Tissue Samples)

Original text:

A tumor block sample obtained within 10 days prior to anticipated enrollment on study will be analyzed for ErbB2 and EGFR expression. Only patients with breast tumors which over-express EGFR by IHC and/or ErbB2 by IHC or fluorescence in situ hybridization (FISH) or which express activated phosphorylated EGFR and/or ErbB2 determined by semi-quantitative IHC will be eligible for this study. The screening tumor samples may be processed locally or by a contract laboratory specified by GSK.

For eligible patients, pre-dose tumor and normal tissue biopsy samples may be obtained up to ten days prior to initiating dosing with GW572016. Three core needle biopsies will be required (two tumor core needle biopsies and one normal breast tissue core needle biopsy obtained adjacent to the tumor).

Following 14 to 21 days of treatment, patients administered GW572016 once daily will have a post-treatment resection or biopsy performed approximately 24 hours following the previous day’s dose while those administered GW572016 twice daily will have their resection or biopsy performed approximately 12 hours following the previous day’s second dose to temporally coincide with the 24 hour steady state trough concentrations. If post-treatment tissue is obtained by biopsy, three core needle biopsies will be required as described above. If tumor tissue is obtained by resection, a minimum of 250 mg of tumor and normal tissue is required for biomarker analysis. If sufficient resected tumor tissues is available, transcriptome analysis using microarray will be performed to examine the molecular profile of tumor tissue for factors which may be predictive of response to GW572016 treatment. In patients for whom surgical resection occurs prior to reaching steady state drug concentrations (e.g. prior to 14 days of treatment), tumor tissue will be evaluated for transcriptome analysis using microarry only.

Changed to read:

Tumor tissue from an archived block sample or from a current biopsy if no archived tumor tissue is available will be analyzed for ErbB2 and EGFR expression. Only patients with breast tumors which over-express EGFR by IHC and/or ErbB2 by IHC or fluorescence in situ hybridization (FISH) or which express activated phosphorylated EGFR and/or ErbB2 determined by semi-quantitative IHC will be eligible for this study. The tumor samples for screening may be processed locally or by a contract laboratory specified by GSK.

For eligible patients, a pre-dose tumor biopsy sample will be obtained within 3 days prior to initiating dosing with GW572016. Three core needle biopsies will be required for biomarker and pharmacokinetic analysis.

On Day 10 to 14, patients administered GW572016 once daily will have a post-treatment surgical resection performed approximately 24 hours (± 3 hours) following the previous day's dose while those administered GW572016 twice daily will have their surgical resection performed approximately 12 hours (± 3 hours) following the previous day's second dose to temporally coincide with the 24 hour steady state trough concentrations. A minimum of 250 mg of tumor is required for biomarker and intratumoral GW572016 concentration analysis.

Section 7.1 Description of Investigational Product

Original text:

Patients will receive tablets of GW572016 ditosylate salt. GW572016 will be supplied to the study site as 250mg oral tablets. The number of tablets and administration schedule will be dependent upon the dose randomization assignment.

Changed to read:

GW572016 will be supplied to the study site as 250mg oral tablets in bulk bottles containing 100 tablets per bottle. Each tablet contains 405 mg of GW572016 ditosylate monohydrate salt equivalent to 250 mg GW572016 free base.

The 250 mg tablets are oval, biconvex, orange, film-coated with one side plain and the opposite side de-bossed with FG HLS.

The number of tablets and administration schedule will be dependent upon the dose randomization assignment and the number of days between enrollment and scheduled surgical resection.

Section 7.2 Dosage and Administration

Original text:

Patients will be randomized to one of 2 dose groups as detailed below. A randomized treatment assignment will be generated and supplied to each participating study center by GSK.

Randomization Group	A	B
Dose / Dosing Schedule	1000mg QD	500mg BID

Changed to read:

Patients will be randomized to one of 3 dose groups as detailed below. A randomized treatment assignment will be generated and supplied to each participating study center by GSK.

Randomization Group	A	B	C
Dose / Dosing Schedule	1500 mg QD	1000 mg QD	500 mg BID

Section 7.3 Dose Rationale

Original text:

Based on pharmacodynamic, clinical response, and safety data from studies EGF10003 and EGF10004 (see below), the doses to be examined in the randomized portion of this study will be 1000mg once daily compared to 500mg twice daily.

Tumor biomarker changes and clinical responses were observed in study EGF10004 for doses of GW572016 at 650mg once daily and above. The frequency of achieving a ≥75% inhibition of one of four key biomarkers (p-EGFR, p-ErbB2, p-ERK1/2, or p-AKT) was 25% at 500mg, 66% at 650mg, 60% at 900mg, 75% at 1200mg, and 80% at 1600mg once daily. A dose of 1000mg daily appears to be in the active range as both biologic and clinical responses have been observed. Study EGF10003 examined three different twice daily doses, 900mg BID, 750mg BID, and 500mg BID. Dose-limiting toxicity (diarrhea) was observed at the 900mg BID dose (2 of 6 subjects) and preliminary data suggests the 750mg BID dose is poorly tolerated by patients (although no DLT has been observed at 750mg BID). Twice daily dosing also results in significant increases in exposure (AUC ~two-fold higher in 900 mg BID dosing compared with 1800 mg once daily). Thus, the 500 mg BID dose would be expected to have increased exposure compared with 1000 mg once daily, but variability in the pharmacokinetic profiles makes dosing based on exposure difficult. Therefore, the doses were chosen based on equivalent total daily dose. GW572016 is currently available as a 250 mg tablet (not scored).

The doses in this study will allow assessment of tumor response (i.e. inhibition of downstream mediators of tumor cell growth and survival) to a total daily dose of 1000mg in a once daily versus twice daily dosing regimen.

A GW572016 250 mg dose is of interest as a potential preventive therapy in subjects with a high risk of developing breast cancer. EGFR has been shown to play an important role in normal ductal epithelium as the major controller of proliferation. The EGFR signaling network in these normal cells may indeed exist in a 'steady state' that is much different than the aberrant signaling networks driving cell growth and proliferation in tumor cells. The *in vivo* pharmacodymic effects of GW572016 on normal breast ductal epithelium therefore, may be quite different than the effects of GW572016 on tumor cells. The dose required to inhibit the EGFR signaling cascade in normal breast ducts may be less than the therapeutic dose needed to treat cancer cells since the normal duct cells rely more heavily on this one pathway and lack many of the molecular abnormalities driving proliferation in tumor cells. In addition, the degree of EGFR inhibition required may be less in the preventive setting as induction of apoptosis would be undesirable in this setting. This portion of the study will determine the safety, tolerability, and pharmacodynamic response of normal breast tissue to GW572016. The information

gained in this study will assist in assessing the potential for low-dose GW572016 to be used as preventive therapy in subjects with a high risk of developing breast cancer.

Changed to read:

Based on pharmacodynamic, clinical response, and safety data from studies EGF10003 and EGF10004 (see below), the doses to be examined in the randomized portion of this study will be 1500 mg and 1000 mg once daily and 500 mg twice daily.

Tumor biomarker changes and clinical responses were observed in study EGF10004 at doses of GW572016 ranging from 650 mg to 1600 mg once daily. The frequency of achieving a ≥75% inhibition of one of four key biomarkers (p-EGFR, p-ErbB2, p-ERK1/2, or p-AKT) was 29% at 500mg, 50% at 650mg, 44% at 900mg, 75% at 1200mg, and 83% at 1600mg once daily. Total daily doses of 1000 mg and 1500 mg daily appear to be in the active range as both biologic and clinical responses have been observed. Study EGF10003 examined three different twice daily doses, 900mg BID, 750mg BID, and 500mg BID. Dose-limiting toxicity (diarrhea) was observed at the 900mg BID dose (2 of 6 subjects) and preliminary data suggests the 750mg BID dose is poorly tolerated by patients (although no DLT has been observed at 750mg BID). Twice daily dosing also results in significant increases in exposure (AUC ~two-fold higher in 900 mg BID dosing compared with 1800 mg once daily). Thus, the 500 mg BID dose would be expected to have increased exposure compared with 1000 mg once daily, but variability in the pharmacokinetic profiles makes dosing based on exposure difficult. Therefore, the doses were chosen based observations of clinical and biologic response as well as tolerability. GW572016 is currently available as a 250 mg tablet (not scored).

The doses in this study will allow assessment of tumor response (i.e. inhibition of downstream mediators of tumor cell growth and survival) in GW572016 dosing regimens of 1000 mg and 1500 mg once daily and 500 mg twice daily.

Section 7.6 Handling and Storage, 3rd paragraph

Original text:

On Day 1 and Day 14-19, study drug will be administered to the patient at the study site after pre-dose safety and PK/PD assessments are complete. On Day 1, an adequate supply of study drug will be dispensed to the patient to account for dosing for the remainder of the study.

Changed to read:

On Day 1 study drug will be administered to the patient at the study site after pre-dose safety and PK/PD assessments are complete. An adequate supply of study drug will be dispensed to the patient to account for dosing for the remainder of the study.

Section 8.2 Prohibited Medications

Original text:

Patients should not receive any other anti-cancer therapy (cytotoxic, biologic, or hormonal other than for replacement) while enrolled on the study. Patients should not receive any other investigational medications within 28 days prior to study enrollment until 28 days after the last dose of study medication.

The following list of medications/substances are prohibited from screening through discontinuation form the study:

- Antibiotics: clarithromycin, erythromycin, troleandomycin, ciprofloxacin, rifampin, norfloxacin, rifabutin.
- HIV Anitretrovirals: delaviridine, indinavir, nelfinavir, ritonavir, saquinivir, efavirenz, nevirapine, amprenavir, lopinavir.
- Anticonvulsants: phenytoin, carbamezepine, phenobarbital.
- Antidepressants: fluoxetine, nefazodone, fluvoxamine.
- Antifungals: itraconazole, ketoconazole, fluconazole, voriconazole.
- GI: antacids (within 1 hour before and after dosing), cimetidine.
- Miscellaneous: glucocorticoids, amiodarone, diltiazem, proglitazone, St. John's Wort, grapefruit or grapefruit juice, mibefradil, diethyldithiocarbamate, gestodene, mifepristone, modafinil.

Changed to read:

Patients should not receive other anti-cancer therapy (cytotoxic, biologic, radiation, or hormone other than for replacement) while on treatment in this study. Patients should not receive any other investigational drugs from 4 weeks prior to the first dose of GW572016 until 28 days after the last dose of study or post-treatment blood draws are completed, whichever is earlier. GW572016 is a substrate for CYP3A4. Inducers and inhibitors of CYP3A4 may alter the metabolism of GW572016. The following list of CYP3A4 inducers and inhibitors are prohibited from screening through discontinuation from the study:

CYP3A4 Inducers:

Antibiotics: all rifamycin class agents (e.g., rifampin, rifabutin, rifapentine)

Anticonvulsants: phenytoin, carbamazepine, phenobarbital (barbiturates)

HIV: efavirenz, nevirapine

Miscellaneous: modafinil, St. John's Wort

WASH-OUT Period: At time of screening, if a patient is currently receiving any of the above listed medications/substances, the medication or substance must be discontinued for a period of no less than **14** days prior to the administration of the first dose of study drug in order for the patient to meet study eligibility

CYP3A4 Inhibitors:

Antibiotics: clarithromycin, erythromycin

HIV: delaviridine, nelfinavir, amprenavir, ritonavir, indinavir, saquinavir

Antifungals: itraconzaole, ketoconazole, fluconazole (> or = to 200 mg daily), voriconazole

Antidepressants: nefazodone, fluovoxamine

Calcium channel blockers: verapamil, diltiazem

GI: antacids: H2 blockers (cimetidine, ranitidine, nizatidine, famotidine), proton pump inhibitors (omeprazole, esomeprazole, rabeprazole, pantoprazole, lansoprazole), antacids (within 1 hour before and after dosing)

Miscellaneous: amiodarone* (also a CYP3A4 substrate), grapefruit or grapefruit juice

WASH-OUT Period: At time of screening, if a patient is currently receiving any of the above listed medications/substances, the medication or substance must be discontinued for a period of no less than **7** days prior to the administration of the first dose of study drug in order for the patient to meet study eligibility

*If patient is to be considered eligible, amiodarone must not have been taken for at least 6 months prior to the administration of the first dose of study drug.

Section 9.1 Subject Completion

Original text:

A patient will be considered complete if the patient receives a minimum of 14 days of dosing with GW572016 and pre-dose and post-dose tumor samples are adequate for analysis of biomarkers.

Changed to read:

A patient will be considered complete if the patient receives a minimum of 9 to13 days of dosing with GW572016 and pre-dose and post-dose tumor samples are adequate for analysis of biomarkers.

Section 11.1 Hypothesis

Original text:

No formal hypotheses are being tested. The study will focus on obtaining point estimates and 90% confidence intervals of differences in pre-to-post optical density changes in tumor tissue markers between QD and BID dosing.

Changed to read:

No formal hypotheses are being tested. The study will focus on obtaining point estimates and 90% confidence intervals of differences in pre-to-post optical density changes in tumor tissue markers for 3 different dosing regimens.

Section 11.2 Sample Size Considerations

Original text:

Subjects will be randomized in equal numbers to QD and BID schedules. Twenty evaluable subjects are targeted for each schedule

An additional 10-20 subjects will be allocated to a dose of 250mg of GW572016.

Changed to read:

Subjects will be randomized in equal numbers to the three dosing schedules. Twenty evaluable subjects are targeted for each schedule.

Section 11.2.1 Sample Size Assumptions

Original text:

Although the targeted number of subjects in this study is based primarily on logistic feasibility, calculations are provided below to show the impact of the using the targeted numbers on the width of 90% confidence intervals.

From preliminary biomarker data from study EGF10004, at a dose of 1200 mg of GW572016, the estimated between-subject standard deviation in the percent pre-dose to post-dose change in optical density ranged from 19% in pAKT to 70% in cyclin D.

When the sample size in each group is 20, a two-sided 90% confidence interval for the difference of two means will extend 36% from the observed difference in means assuming that the common standard deviation in the two treatment groups is known to be 70% and the confidence interval is based on the large sample z statistic.

For example, if the observed mean percent decrease in a given biomarker’s activity for one schedule were 50% and 80% for the other schedule, a 90% confidence interval for the true difference in schedule mean percent change in the marker would be 30% ± 36%.

Changed to read:

In preliminary analysis of the tumor biomarkers in the phase I clinical study EGF10004, the standard deviation of the pre-dose to post-dose percent change in individual biomarkers ranged from 17% to over 600% across multiple dose levels of GW572016. Although the variability of tumor biomarkers measured in a more homgenous population (i.e. all breast cancer) may be reduced, it is not practical to frame this study as confirmatory hypothesis testing. Thus, results from this study should be viewed as exploratory data analysis, and proposed sample sizes of each treatment group are based on logistic feasibility.

Section 11.3 Analysis Populations

Original text:

All subjects who receive at least one dose of GW572016 will be included in the safety population. All patients for whom valid GW572016 pharmacokinetic parameters can be estimated will be included in the pharmacokinetic population. All patients who receive a minimum of 14 days of treatment and for whom pre- and post-dose biomarker values have been obtained will be included in the pharmacodynamic population.

Changed to read:

All subjects who receive at least one dose of GW572016 will be included in the safety population. All patients for whom valid GW572016 pharmacokinetic concentrations can be estimated will be included in the pharmacokinetic population. All patients who receive 9 to 13 days of treatment and for whom pre- and post-dose biomarker values have been obtained will be included in the pharmacodynamic population.

Section 11.6.1 Pharmacokinetic Analyses

Original text:

GW572016 plasma concentration data will be summarized and displayed in both tabular and graphical form. Non-compartmental analysis of concentration time data will be performed using standard methods. The pharmacokinetic parameters $AUC_{\tau,}$ C_{max}, and t_{max}, will be calculated for Days 1 and 14-19. Individual patient parameter values, as well as a descriptive summary (mean, standard deviation, median, minimum, maximum, and the standard deviation and geometric mean of log-transformed parameters) by treatment group will be reported. Individual patient parameter values (AUC, $C_{max,}$) will be plotted versus treatment group.

Drug accumulation will be evaluated by estimating pertinent pharmacokinetic parameters in each subject on Days 1 and 14-19. Drug accumulation results will be listed by subject and summarized by treatment group.

Dose proportionality of AUC and C_{max} for GW572016 on Day 1 will be evaluated visually in graphical form.

Changed to read:

GW572016 plasma and intratumoral concentration data will be summarized and displayed in both tabular and graphical form.

Section 11.6.2 Pharmacodynamic Analyses

Original text:

Each patient will be measured for biomarker activity level in tissue and tumor obtained pre- and post-dose. For each marker, a one-way analysis of covariance of the post-dose measurement will be performed with treatment group as a classification variable and the pre-dose level of the marker as a covariate. Where possible, comparisons will be constructed to estimate the least squares mean difference between the QD and BID schedules for each total daily dose of GW572016. Transformation, e.g., logarithms, of pre- and post-dose marker levels may be necessary to satisfy analysis assumptions.

Analysis of transcrioptome and proteomic data will be the responsibility of the Discovery Bioinformatics (DB). Statistical analyses of the transcriptome and proteomic data will be the responsibility of Clinical Pharmacology Statistics and Data Sciences, and will be performed in consultation with DB. All pharmacodynamic endpoints of interest from all study sessions will be descriptively and/or graphically summarized, as appropriate to the data.

For each gene separately, adjusted abundances will be determined for each time point within each regimen and subject. Following normalizaton of these adjusted abundances, appropriate methods may be used to eliminate invalid adjusted abundances from the data set prior to analysis. For each comparison of interest, adjusted abundances for a single gene will be pooled across subjects and will then be statistically analyzed by currently accepted methods for microarray data, such as t-tests, taking into account the design of the study. As appropriate, adjustments for multiple comparisons will be made. [Dudoit, 2000]. An examination of the association between fold ratio changes with the expression level of selected genes (e.g., cytokines) will be done in an exploratory fashion, by comparison with literature/ELISA data, as appropriate.

Changed to read:

Each patient will be measured for biomarker activity level in tumor obtained pre- and post-dose. For each marker, a one-way analysis of covariance of the post-dose measurement will be performed with treatment group as a classification variable and the pre-dose level of the marker as a covariate. Adjusted least squares means will be estimated for each dose regimen. Tansformation, e.g., logarithms, of pre- and post-dose marker levels may be necessary to satisfy analysis asssumptions.

Analysis of proteomic data will be the responsibility of the Discovery Bioinformatics (DB). Statistical analyses of the proteomic data will be the responsibility of Clinical Pharmacology Statistics and Data Sciences, and will be performed in consultation with DB. All pharmacodynamic endpoints of interest from all study sessions will be descriptively and/or graphically summarized, as appropriate to the data.

Deleted text:

Treatment Comparisons of Interest (Original Section 11.2)

Primary Comparison of Interest (Original Section 11.2.1)

For each biomarker assessed in biopsied tumor tissue, the primary comparison of interest is the difference in the mean pre-dose to post-dose percent change in the optical density resulting from QD versus BID dosing schedules for a total daily dose of 1000 mg of GW572016.

Other Comparisons of Interest (Original Section 11.2.2)

Pre-dose to post-dose percent change in optical density resulting from 250 mg QD dosing of GW572016 will be summarized but will not be formally compared to other treatment groups. Marker changes in normal tissue will also be assessed for the 250 mg dose group and the 500 mg BID and 1000 mg QD dose groups.

Interim Analysis (Original Section 11.3)

No interim analysis will be performed.

Sample Size Sensitivity (Original Section 11.4.2)

If between subject standard deviation of pre-to-post exposure change were 50% or greater than seen in EGF10004, i.e., 105%, with the same sample size, then a two-sided 90% confidence interval for the difference of two means will extend 55% from the observed difference in means.

For example, if the observed mean percent decrease in a given biomarker's activity for one schedule were 50% and 80% for the other schedule a 90% confidence interval for the true difference in schedule means would be 30% ± 55%.

If between subject standard deviation of pre-to-post exposure change were 50% less than see in EGF10004, i.e., 35%, with the same sample size, then a two-sided 90% confidence interval for the difference of two means will extend 18% from the observed difference in means.

For example, if the observed mean percent decrease in a given biomarker's activity for one schedule were 50% and 80% for the other schedule, a 90% confidence interval for the true difference in schedule means would be 30% ± 18%.

Sample Size Re-estimation (Original Section 11.4.3)

There is no plan to re-estimate sample sizes.

Derived and Transformed Data (Original Section 11.6.3)

Pharmacokinetic parameters relating to AUC and C_{max}, will be log-transformed prior to analysis.

Section 12.2 Study Monitoring

Deleted text:

The monitor will also review subject-completed health outcomes questionnaire(s) for extraneous written comments that could indicate possible AEs. Information collected in the CRF and in the subject-completed health outcomes questionnaire(s) are independent components of this study. Except for header section information (e.g., subject number, treatment number, visit date) and other information as defined in the standard clarification agreement (SCA), neither the monitor nor the investigator will attempt to reconcile responses to individual questions/items recorded on the subject-completed health outcomes questionnaire(s) or health outcomes portions of diary cards (if applicable) with other data recorded in the CRFs. Subject-completed health outcome questionnaires generally serve as the source document; therefore, unless otherwise specified elsewhere, no other source document is available for data validation.

Section 14.1 Appendix 1

Original text:

Appendix 1: Time and Events Table

Study Assessments	Pre-Study[a]	Day 1[a]	Day 14-20 (prior to surgical resection or biopsy)	Day 15-21	Post Study[b]
Informed Consent	X				
Physical Exam	X	X	X		X
Medical and Medication History	X				
Vital Signs	X	X	X		X
Clinical Labs[c]	X	X	X		X
12-lead ECG	X				
MUGA[d]	X				
AE Monitoring and Concomitant Medications	Continuous				
Dosing		X	X		
Tumor Biopsy/Resection[e]	X			X	
Pharmacokinetic[f] Sampling		X	X		
Pharmacodynamic[g] Sampling		X	X		X
Pharmacogenetic Sampling[h]		X			

a. All pre-study screening assessments should be scheduled within two weeks of the first dose of study drug. Screening assessments completed within 72 hours of first dose of study drug can be used as Day 1 assessments. The pre-dose tumor biopsy must be obtained within 10 days of anticipated enrollment on study. EGFR/ErbB2 expression results must be available prior to dosing on Day 1.
b. The follow-up visit should occur 28 days after the last dose of study medication or prior to initiating chemotherapy.
c. Refer to Appendix 2 for complete list of required clinical labs. Pregnancy testing is required for all women of child-bearing potential.
d. LVEF must be >40% by MUGA to be eligible for enrollment in the study.
e. The post-treatment tumor resection/biopsy must coinicide with the previous day's GW572016 dose trough concentration $\pm$ 2 hours.
f. Refer to Section 6.6 for pharmacokinetic sampling schedule.
g. Refer to Section 6.7 for pharmacodynamic sampling schedule.
h. Refer to Section 6.8 for pharmacogenetic sampling schedule.

Changed to read:

Appendix 1: Time and Events Table

Study Assessments	Pre-Study[a]	Day 0 or 1	Day 10 to 14	Post Study[b]
Informed Consent	X			
Physical Exam	X	X	X	X
Medical and Medication History	X			
Vital Signs	X	X	X	X
Clinical Labs[c]	X	X	X	X
12-lead ECG	X			
MUGA or ECHO[d]	X			
AE Monitoring and Concomitant Medications	Continuous			
Dosing[e]		X		
Tumor Resection[f]	X		X	
Pharmacokinetic[g] Sampling		X	X	
Pharmacodynamic Sampling[h]		X	X	X
Pharmacogenetic Sampling[i]		X		

a. All pre-study screening assessments should be scheduled within two weeks of the first dose of study drug. Screening assessments completed within 72 hours of first dose of study drug can be used as Day 1 assessments. The pre-dose tumor biopsy must be obtained within 3 days of anticipated enrollment on study. EGFR/ErbB2 expression results must be available prior to dosing on Day 1.

b. The follow-up visit should occur 28 days after the last dose of study medication or prior to initiating adjuvant chemotherapy.

c. Refer to Appendix 2 for complete list of required clinical labs. Pregnancy testing is required for all women of child-bearing potential.

d. LVEF must be >40% by MUGA or ECHO to be eligible for enrollment in the study.

e. Study drug will be dosed daily according to the randomization assignment from Day 1 until Day 9 to 14 (the day prior to the scheduled resection).

f. The post-treatment tumor resection must coinicide with the previous day's GW572016 dose trough concentration ± 3 hours.

g. Refer to Section 6.6 for pharmacokinetic sampling schedule.

h. Refer to Section 6.7 for pharmacodynamic sampling schedule.

i. Refer to Section 6.8 for pharmacogenetic sampling schedule.

14.7. Appendix 7: Amendment 02 Summary of Changes

Global Changes:

Throughout the protocol Common Toxicity Criteria (CTC) version 2 was replaced with Common Terminology Criteria for Adverse Events (CTCAE) version 3, Day 0 and 1 was replaced with Day 1 and GW572016 was replaced with the generic name of lapatinib and EGFR was replaced with ErbB2.

PROTOCOL SUMMARY

Study Design

Original Text:

Prior to entering the study, the EGFR and/or ErbB2 status will be determined for each patient from a recent biopsy. Only patients with treatment-naïve, breast tumors measuring greater than 2 cm, which overexpress EGFR by IHC and/or ErbB2 by IHC or fluorescence in situ hybridization (FISH), or which express activated phosphorylated EGFR and/or ErbB2 determined by semi-quantitative IHC will be eligible for this study. For eligible patients, an additional biopsy will be obtained pre-treatment and tumor tissue will be examined by semi-quantitative IHC for biomarkers involved in regulating tumor cell proliferation and survival (e.g., ErbB2, ERK1/2, phosphorylated-ERK1/2, AKT, phosphorylated AKT and cyclin D1 protein and potentially other downstream markers of kinase activity). Patients will receive treatment with GW572016 for 9 to 13 days in the interim period between initial diagnosis and scheduled surgical resection. Tumor tissue obtained at resection will be examined for biomarkers as described above and compared to the results from the pre-treatment tumor sample. In addition, blood and tumor will be obtained at the time of resection and will be examined for steady state, trough concentrations of GW572016.

Changed to read:

Prior to entering the study, the ErbB1 and/or ErbB2 status will be determined for each patient. Only patients with treatment-naïve, breast tumors measuring 1 cm or greater which express ErbB1 and/or overexpress ErbB2 by semi-quantitative IHC or demonstrate ErbB2 gene amplification by fluorescence in situ hybridization (FISH), or which express activated phosphorylated ErbB1 and/or ErbB2 by semi-quantitative IHC will be eligible for this study. For eligible patients, if archived biopsy tissue is used to determine eligibility, an additional biopsy obtained within 14 days prior to initiating study treatment will be required. The pre-treatment biopsy tissue will be examined by semi-quantitative IHC for biomarkers involved in regulating tumor cell proliferation and survival (e.g., ErbB2, ERK1/2, phosphorylated-ERK1/2, AKT, phosphorylated AKT and cyclin D1 protein and potentially other downstream markers of kinase activity). Patients will receive treatment with lapatinib for 9 to 13 days prior to surgical resection. Tumor tissue obtained at resection will be examined for biomarkers as described above and compared to the results from the pre-treatment biopsy. In addition, blood and tumor obtained at the

time of resection and will be examined for steady state, trough concentrations of lapatinib.

Study Population

Original text:

A sufficient number of patients will be enrolled to obtain 60 evaluable patients (20 per dose group) at 5-6 study centers. Male or female patients, aged 18 years or older with a breast tumor measuring at least 2 cm with evidence of EGFR and/or ErbB2 overexpression will be enrolled

Change to read:

A sufficient number of patients will be enrolled to obtain 60 evaluable patients (20 per dose group) at approximately 10 study centers. Male or female patients, aged 18 years or older with a breast tumor measuring at least 1 cm with evidence of EGFR and/or ErbB2 overexpression will be enrolled.

Study Population (eligibility criteria)

Original text:

b. Childbearing potential, has a negative serum pregnancy test at Screening and agrees to one of the following:

- Double-barrier contraception (condom with spermicidal jelly, foam suppository, or film; diaphragm with spermicide; or male condom and diaphragm).
- Complete abstinence form sexual intercourse from 2 weeks prior to administration of the study drug, throughout the active study treatment period, and through the follow-up visit (to occur 28 days after last dose of study medication).
- Vasectomized partner who is sterile prior to the female subject's entry and is the sole sexual partner for that female.

Changed to read:

b. Childbearing potential, has a negative serum pregnancy test at Screening and agrees to one of the following where considered acceptable by the institution IEC or IRB:

- Complete abstinence form sexual intercourse from 2 weeks prior to administration of the study drug, throughout the active study treatment period, and through the follow-up visit (to occur 28 days after last dose of study medication).
- Barrier contraception (condom with spermicidal jelly, foam suppository, or film; diaphragm with spermicide; or male condom and diaphragm).
- Male partner who is who is sterile prior to the female subject's entry and is the sole sexual partner for that female subject.
- Implants of levonorgesterol.

- Injectable progestogen.
- Any intrauterine device (IUD) with a documented failure rate of less than 1% per year.
- Oral contraceptives (either combined or progetogen only)

Preliminary Results from Healthy Volunteers and Patients

Deleted Text:

Two additional healthy volunteer studies have been conducted. In these studies a total of 21 (EGF10008) and 70 (EGF10024) subjects have received single GW572016 doses, 100 mg and 250 mg, respectively. All AEs in EGF10008 were considered mild or moderate with headache, nasal congestion, and pruritus of hands the most frequent AEs seen. One subject was withdrawn from this study due to bilateral swelling of hands and forearms. The investigator assessed that the event was related to study drug. Data from EGF10024 is currently being evaluated.

The first study in cancer patients (EGF10003) is ongoing. Daily doses of up to 1800 mg have been administered. A maximum tolerated dose (MTD) was not achieved following once daily dosing. Only Grade 1 and Grade 2 toxicity (preliminary data) have been seen following daily dosing. Those AEs which have occurred most frequently include diarrhea (18-25%), rash (<10%) and nausea (< 10%). Preliminary pharmacokinetic data from EGF10003 indicate that serum concentrations increase in a generally proportional manner with increasing dose up to 1800 mg. In this study 6 patients have received 900 mg twice daily and 8 patients have received 750mg twice daily. There has been an increased incidence of gastrointestinal (GI) intolerance (nausea, vomiting and diarrhea) to approximately 34% in patients receiving twice daily dosing. This may be due to a local GI effect or may be due to enhanced absorption and increased bioavailability.

The second study in cancer patients (EGF10004) is also in progress. Preliminary AE data in this study is similar to that seen in EGF10003. AEs have been primarily Grade 1 or 2 and consist of diarrhea (30%) and rash (12%). There was one report of Grade 3 reflux which was considered related to a large pill burden. In general, the time to AE onset was 2-4 weeks following initiation of daily dosing.

There are five additional Phase I patient studies ongoing. These are combination studies evaluating GW572016 in combination with capecitabine (EGF10005), paclitexel (EGF10009), docetaxel (EGF10021), oxaliplatin/flurouracil/leucovorin (EGF10010) and ironotecan/flurouracil/leucovorin (EGF10011).

Changed to Read:

Refer to the the current Clinical Investigator’s Brochure / Investigator’s Brochure (CIB/IB) for additional information on studies in healthy volunteers and cancer patients.

4. Study Design

Original Text:

Prior to entering the study, the EGFR and/or ErbB2 status will be determined for each patient from a recent biopsy. Only patients with treatment-naïve, breast tumors measuring greater than 2 cm, which overexpress EGFR by IHC and/or ErbB2 by IHC or fluorescence in situ hybridization (FISH), or which express activated phosphorylated EGFR and/or ErbB2 determined by semi-quantitative IHC will be eligible for this study. For eligible patients, an additional biopsy will be obtained pre-treatment and tumor tissue will be examined by semi-quantitative IHC for biomarkers involved in regulating tumor cell proliferation and survival (e.g., ErbB2, ERK1/2, phosphorylated-ERK1/2, AKT, phosphorylated AKT and cyclin D1 protein and potentially other downstream markers of kinase activity). Patients will receive treatment with GW572016 for 9 to 13 days in the interim period between initial diagnosis and scheduled surgical resection. Tumor tissue obtained at resection will be examined for biomarkers as described above and compared to the results from the pre-treatment tumor sample. In addition, blood and tumor will be obtained at the time of resection and will be examined for steady state, trough concentrations of GW572016.

Changed to Read:

Prior to entering the study, the ErbB1 and/or ErbB2 status will be determined for each patient.. Patients with treatment-naïve breast tumors measuring 1 cm or greater which express ErbB1 (+) and/or overexpress ErbB2 (2+ or 3+) by semi-quantitative IHC or demonstrate amplification of the ErbB2 gene by fluorescence in situ hybridization (FISH), or which express activated phosphorylated ErbB1 and/or ErbB2 determined by semi-quantitative IHC will be eligible for this study. For eligible patients, if archived biopsy tissue is used to determine eligibility, an additional biopsy obtained within 14 days prior to initiating study treatment will be required. The pre-treatment biopsy tissue will be examined by semi-quantitative IHC for biomarkers involved in regulating tumor cell proliferation and survival (e.g., ErbB2, ERK1/2, phosphorylated-ERK1/2, AKT, phosphorylated AKT and cyclin D1 protein and potentially other downstream markers of kinase activity). Patients will receive treatment with lapatinib for 9 to 13 days prior to surgical resection.Tumor tissue obtained at resection will be examined for biomarkers as described above and compared to the results from the pre-treatment tumor sample. In addition, blood and tumor obtained at the time of resection and will be examined for steady state, trough concentrations of lapatinib.

5.1 Number of Subjects

Original Text:

A sufficient number of patients will be enrolled to obtain 60 evaluable patients (20 per dose group) at 5-6 study centers.

Change to Read:

A sufficient number of patients will be enrolled to obtain 60 evaluable patients (20 per dose group) at approximately 10 study centers.

5.2.1 Inclusion Criteria

Original Text:

A subject will be eligible for inclusion in this study only if all of the following criteria apply:

1. Has a histologically confirmed, treatment-naïve, breast tumor measuring at least 2 cm that can be readily biopsied and that EITHER (i) over-expresses total EGFR by IHC, ErbB2 by IHC and/or FISH gene amplification, (ii) or expresses activated, phosphorylated EGFR or ErbB2.

Change to read:

A subject will be eligible for inclusion in this study only if all of the following criteria apply:

1. Has a histologically confirmed, treatment-naïve, breast tumor measuring 1 cm or greater that can be readily biopsied to meet protocol requirements and that EITHER (i) expresses total ErbB1 (EGFR) by IHC (positive), ErbB2 by IHC 2+ or 3+ and/or FISH gene amplification (positive), (ii) or expresses activated, phosphorylated EGFR or ErbB2.

Original Text:

b. Childbearing potential, has a negative serum pregnancy test at Screening and agrees to one of the following:

- Double-barrier contraception (condom with spermicidal jelly, foam suppository, or film; diaphragm with spermicide; or male condom and diaphragm).
- Complete abstinence form sexual intercourse from 2 weeks prior to administration of the study drug, throughout the active study treatment period, and through the follow-up visit (to occur 28 days after last dose of study medication).
- Vasectomized partner who is sterile prior to the female subject’s entry and is the sole sexual partner for that female.

Changed to read:

b. Childbearing potential, has a negative serum pregnancy test at Screening and agrees to one of the following where considered acceptable by the institution IEC or IRB:

- Complete abstinence form sexual intercourse from 2 weeks prior to administration of the study drug, throughout the active study treatment period, and through the follow-up visit (to occur 28 days after last dose of study medication).

- Barrier contraception (condom with spermicidal jelly, foam suppository, or film; diaphragm with spermicide; or male condom and diaphragm).
- Male partner who is who is sterile prior to the female subject's entry and is the sole sexual partner for that female subject.
- Implants of levonorgesterol.
- Injectable progestogen.
- Any intrauterine device (IUD) with a documented failure rate of less than 1% per year.
- Oral contraceptives (either combined or progetogen only).

5.2.2 Exclusion Criteria

Deleted Text:

9. Currently receiving steroid (oral, inhaled) treatment.

Original Text:

1. Has received prior biological, cytotoxic or hormonal (other than for replacement) therapy to treat breast cancer.

2. Has received prior radiation therapy to the chest, mediastinum or abdomen.

7. Has a known immediate or delayed hypersensitivity reaction or idiosyncrasy to drugs chemically related to the study drug. There are a number of small molecules such as ZD1839 [Iressa], OSI-774 that are in clinical development and other ErbB2 or EGFR inhibitors that are monoclonal antibodies in clinical development or on the market [Herceptin, C225]).

Changed to Read:

1. Has received prior biological, cytotoxic or hormonal (other than for replacement) therapy to treat this incidence of breast cancer.

2. Has received prior radiation therapy to the chest to treat this incidence of breast cancer.

7. Has a known immediate or delayed hypersensitivity reaction or idiosyncrasy to drugs chemically related to the study medication. These include other anilinoquinazolines, such as gefitinib [Iressa], erlotinib [Tarceva], or other chemically related compounds.

5.2.3 Other Eligibility Criteria Considerations

Deleted Text

Patients must refrain from alcohol for the duration of the study.

6.3 On Study Assessments

Original Text

Full physical examination, including vital signs (blood pressure and heart rate) to occur at Day 0 or 1 (if greater than 72 hours since screening physical exam) and prior to scheduled surgical resection (Day 9 to 13).

Clinical Chemistry on Day 0 or 1 and prior to scheduled surgical resection on Day 9 to 13.

Hematology on Day 0 or 1 and prior to scheduled surgical resection on Day 9 to 13.

Change to Read

- Full physical examination, including vital signs (blood pressure and heart rate) to occur at Day 1 (if greater than 14 days since screening physical exam) and prior to scheduled surgical resection (Day 9 to 13).
- Clinical Chemistry on Day 1 (if greater than 14 days since screening chemistries) and prior to scheduled surgical resection on Day 9 to 13.
- Hematology on Day 1 (if greater than 14 days since screening hematology) and prior to scheduled surgical resection on Day 9 to 13.
- Assessment of study medication compliance when study medication bottle is returned prior to surgical resection.
- Assessment of adverse events continuously.
- Tumor surgical resection (Day 10 to 14).

6.4 Post Study Visit

Original Text:

Twenty-eight days following the last dose of GW572016 (or prior to initiating adjuvant therapy), all patients will be required to have a post-study evaluation to include:

Changed to read:

Seven to Twenty-eight days following the last dose of GW572016 (or prior to initiating adjuvant therapy), all patients will be required to have a post-study evaluation to include:

6.5.2 Clinical Laboratory Tests

Original Text:

Blood and urine will be obtained for clinical laboratory tests prior to dosing on Days 1 and the day prior to surgical resection.

Changed to read:

Blood will be obtained for clinical laboratory tests prior to dosing on Days 1 and the day prior to surgical resection.

6.7.1 Tumor Tissue Samples

Added Text end of the 3rd paragraph:

If for any reason, surgical resection cannot be done within this time period, the GSK medical monitor must be contacted. This discussion must be held prior to the 14th day.

6.8.6 Pharmacogenetic Samples

Added Text, 3rd paragraph:

The sample can be shipped ambient provided it is shipped the same day the sample is drawn.

7.1 Description of Investigational Product

Original Text:

GW572016 will be supplied to the study site as 250mg oral tablets in bulk bottles containing 100 tablets per bottle.

Change to Read:

Laptinib will be supplied to the study site as 250mg oral tablets in bulk bottles containing 90 tablets per bottle.

7.8 Assessment of Compliance

Added Text end of first paragraph:

During the visit the site staff will review the patient-dosing diary and remaining medication to confirm compliance with dosing.

8.2 Prohibited Medications

Original Text:

Patients should not receive other anti-cancer therapy (cytotoxic, biologic, radiation, or hormone other than for replacement) while on treatment in this study. Patients should not receive any other investigational drugs from 4 weeks prior to the first dose of GW572016 until 28 days after the last dose of study or post-treatment blood draws are completed, whichever is earlier. GW572016 is a substrate for CYP3A4. Inducers and inhibitors of CYP3A4 may alter the metabolism of GW572016. The following list of CYP3A4

inducers and inhibitors are prohibited from screening through discontinuation from the study:

CYP3A4 Inducers:

Antibiotics: all rifamycin class agents (e.g., rifampin, rifabutin, rifapentine)

Anticonvulsants: phenytoin, carbamazepine, phenobarbital (barbiturates)

HIV: efavirenz, nevirapine

Miscellaneous: modafinil, St. John’s Wort

WASH-OUT Period: At time of screening, if a patient is currently receiving any of the above listed medications/substances, the medication or substance must be discontinued for a period of no less than **14** days prior to the administration of the first dose of study drug in order for the patient to meet study eligibility

CYP3A4 Inhibitors:

Antibiotics: clarithromycin, erythromycin

HIV: delaviridine, nelfinavir, amprenavir, ritonavir, indinavir, saquinavir

Antifungals: itraconzaole, ketoconazole, fluconazole (> or = to 200 mg daily), voriconazole

Antidepressants: nefazodone, fluovoxamine

Calcium channel blockers: verapamil, diltiazem

GI: antacids: H2 blockers (cimetidine, ranitidine, nizatidine, famotidine), proton pump inhibitors (omeprazole, esomeprazole, rabeprazole, pantoprazole, lansoprazole), antacids (within 1 hour before and after dosing)

Miscellaneous: amiodarone* (also a CYP3A4 substrate), grapefruit or grapefruit juice

WASH-OUT Period: At time of screening, if a patient is currently receiving any of the above listed medications/substances, the medication or substance must be discontinued for a period of no less than **7** days prior to the administration of the first dose of study drug in order for the patient to meet study eligibility

*If patient is to be considered eligible, amiodarone must not have been taken for at least 6 months prior to the administration of the first dose of study drug.

Change to Read:

Drug Class	Agent	Wash-out[1]
CYP3A4 Inducers		
Antibiotics	all rifamycin class agents (e.g., rifampin, rifabutin, rifapentine)	14 days
Anticonvulsants	phenytoin, carbamezepine, barbiturates (e.g., phenobarbital)	
Antiretrovirals	efavirenz, nevirapine	
Glucocorticoids (oral)	Cortisone (>50mg), hydrocortisone (>40mg), prednisone (>10mg), methylprednisolone (>8mg), dexamethasone (>1.5mg)[2]	
Other	St. John's Wort, modafinil	
CYP3A4 Inhibitors		
Antibiotics	clarithromycin, erythromycin, troleandomycin	7 days
Antifungals	itraconazole, ketoconazole, fluconazole (> 150mg daily), voriconazole	
Antiretrovirals	delaviridine, nelfinavir, amprenavir, ritonavir, indinavitr, saquinavir, lopinavir	
Calcium channel blockers	verapamil, diltiazem	
Antidepressants	nefazodone, fluvoxamine	
GI Agents	cimetidine, aprepitant	
Other	grapefruit, grapefruit juice	
	Amiodarone	6 months
Miscellaneous		
H2 blockers	ranitidine, nizatidine, famotidine	2 days
Proton Pump Inhibitors	omeprazole, esomeprazole, rabeprazole, pantoprazole, lansoprazole	2 days
Antacids	Mylanta, Maalox, TUMS, Rennies	1 hour before and after dosing
Herbal or dietary supplements	All	14 days

1. At the time of screening, if a patient is receiving any of the above listed medications/substances, the medication or substance must be discontinued (if clinically appropriate) for the period of time specified prior to administration of the first dose of investigational product and throughout the study period in order for the patient to meet study eligibility.
2. Glucocorticoid daily doses (oral) ≤ 1.5mg of dexamethasone (or equivalent) are allowed. Glucocorticoid conversions are provided in parentheses.

11.5.3 Clinical Laboratory Evaluations

Original Text:

Hematology and clinical chemistryvalues will be listed for each patient and flagged high or low relative to the normal range, where applicable.

Change to Read:

Hematology and clinical chemistry values will be listed for each patient and flagged high or low relative to the normal range, where applicable.

Appendix 1: Time and Events Table

Original Text:

Study Assessments	Pre-Study[a]	Day 0 or 1	Day 10 to 14	Post Study[b]
Informed Consent	X			
Physical Exam	X	X	X	X
Medical and Medication History	X			
Vital Signs	X	X	X	X
Clinical Labs[c]	X	X	X	X
12-lead ECG	X			
MUGA or ECHO[d]	X			
AE Monitoring and Concomitant Medications	Continuous			
Dosing[e]		X		
Tumor Resection[f]	X		X	
Pharmacokinetic Sampling[g]		X	X	
Pharmacodynamic Sampling[h]		X	X	X
Pharmacogenetic Sampling[i]		X		

a. All pre-study screening assessments should be scheduled within two weeks of the first dose of study drug. Screening assessments completed within 72 hours of first dose of study drug can be used as Day 1 assessments. The pre-dose tumor biopsy must be obtained within 3 days of anticipated enrollment on study. EGFR/ErbB2 expression results must be available prior to dosing on Day 1.
b. The follow-up visit should occur 28 days after the last dose of study medication or prior to initiating adjuvant chemotherapy.
c. Refer to Appendix 2 for complete list of required clinical labs. Pregnancy testing is required for all women of child-bearing potential.
d. LVEF must be >40% by MUGA or ECHO to be eligible for enrollment in the study.
e. Study drug will be dosed daily according to the randomization assignment from Day 1 until Day 9 to 13 (the day prior to the scheduled resection).
f. The post-treatment tumor resection must coincide with the previous day's GW572016 dose trough concentration ± 3 hours.
g. Refer to Section 6.6 for pharmacokinetic sampling schedule.
h. Refer to Section 6.7 for pharmacodynamic sampling schedule.
i. Refer to Section 6.8 for pharmacogenetic sampling schedule.

Changed to read:

Study Assessments	Pre-Study[a]	Day 1 (pre-dose)	Day 10 to 14	Post Study[b]
Informed Consent	X			
Physical Exam	X		X	X
Medical and Medication History	X			
Vital Signs	X	X	X	X
Clinical Labs[c]	X		X	X
12-lead ECG	X			
MUGA or ECHO[d]	X			
AE Monitoring and Concomitant Medications	Continuous			
Dosing[e]		X		
Tumor Biopsy / Resection[f]	X		X	
Pharmacokinetic Sampling[g]		X	X	
Pharmacodynamic Sampling[h]		X	X	X
Pharmacogenetic Sampling[i]		X		

a. All pre-study screening assessments should be scheduled within two weeks of the first dose of study drug. Screening assessments completed within 72 hours of first dose of study drug can be used as Day 1 assessments.. If archived tissue used to determine elgibility; an additional biopsy obtained within 14 days of initiating study treatment will be required. ErbB1/ErbB2 expression results must be available prior to dosing on Day 1.
b. The follow-up visit should occur 7-28 days after the last dose of study medication or prior to initiating adjuvant therapy.
c. Refer to Appendix 2 for complete list of required clinical labs. Pregnancy testing is required for all women of child-bearing potential.
d. LVEF must be >40% by MUGA or ECHO to be eligible for enrollment in the study.
e. Study drug will be dosed daily according to the randomization assignment from Day 1 until Day 9 to 13 (the day prior to the scheduled resection).
f. The pre-dose tumor biopsy must be obtained within 14 days of initiating study treatment. The post-treatment tumor resection must coinicide with the previous day's lapatinib dose trough concentration $\pm$ 3 hours.
g. Refer to Section 6.6 for pharmacokinetic sampling schedule.
h. Refer to Section 6.7 for pharmacodynamic sampling schedule.
i. Refer to Section 6.8 for pharmacogenetic sampling schedule.

14.8. Appendix 8: Amendment 03 Summary of Changes

Study Title

Original Text:

A Phase I, Open Label Study of the Safety, Pharmacokinetics and Pharmacodynamics of Lapatinib (GW572016) in Once Daily Versus Twice Daily Dosing Regimens in Patients with Treatment-Naive Breast Cancer

Changed to Read:

A Phase I, Open Label Study of the Safety, Pharmacokinetics and Pharmacodynamics of Lapatinib (GW572016) in Patients with Treatment-Naïve, ErbB2 Positive Breast Cancer

Summary Protocol, Study Population, 1st paragraph

Original Text:

A sufficient number of patients will be enrolled to obtain 60 evaluable patients (20 per dose group) at approximately 10 study centers. Male or female patients, aged 18 years or older with a breast tumor measuring 1 cm or greater with evidence of ErbB1 expression and/or ErbB2 protein overexpression or ErbB2 gene amplification will be enrolled.

Changed to Read:

Approximately 20 evaluable patients will be enrolled from approximately 16 study centers. Female patients, aged 18 years or older with a breast tumor measuring 1 cm or greater with evidence of ErbB2 protein overexpression as measured by IHC 3+ or ErbB2 gene amplification as measured by FISH and who will receive adjuvant chemotherapy, hormonal therapy or radiation therapy will be enrolled. Patients must be naïve to cytotoxic, radiation, hormonal and biologic therapy to treat this incidence of breast cancer.

Removed Text:

A female is eligible to enter and participate in the study if she is of:

Summary Protocol Rationale

Original Text:

Breast cancer is the most common malignancy in women in the United States. Despite a variety of hormonal, cytotoxic and biologic approaches, a significant number of tumors are resistant to currently approved treatment modalities [Ring, 2002]. There is evidence that epidermal growth factor receptor (EGFR, ErbB1) and ErbB2 over-expression in breast cancers are independently associated with more aggressive tumor proliferation and a poor prognosis [Klijn, 1992; Kaptain, 2001]. ErbB1 and ErbB2 are members of the

Type I family of receptor tyrosine kinases (ErbB). Tyrosine autophosphorylation of ErbB1/ErbB2 activates downstream effectors regulating cell proliferation and survival. A compound such as lapatinib that can selectively modulate the abnormal signaling pathways in these tumors is an alternative therapeutic approach in treating breast cancer. Although the efficacy of treatment with lapatinib in the neoadjuvant breast cancer setting is unknown at this time, tumor biomarker data generated from this study will guide future Phase II/III breast cancer efficacy trial design.

FDA approval of Herceptin, a humanized monoclonal antibody targeting ErbB2 effective in treating patients whose breast cancer over-express ErbB2 and/or display ErbB2 gene amplification, demonstrates the clinical utility of ErbB2 targeted therapy. Lapatinib, a reversible inhibitor of both ErbB1 and ErbB2 tyrosine kinases, has been shown to induce growth arrest and/or tumor cell apoptosis in ErbB1 or ErbB2 dependent tumor cell lines or xenografts. The dual inhibitory nature of lapatinib offers a potential therapeutic advantage over a compound that inhibits only one tyrosine receptor kinase.

This study will examine the inhibition of ErbB1 and ErbB2 phosphorylation and downstream mediators of tumor cell growth and survival (e.g., phosphorylated ERK1/2, phosphorylated AKT and cyclin D1 protein and potentially other downstream markers of kinase activity) tumor tissue in treatment-naïve breast cancer patients for three dosing schedules of lapatinib. Patients will be randomized to one of the three dose groups of lapatinib and will receive treatment for 9 to 13 prior to surgical resection of the tumor. Biopsy tissue obtained pre-treatment (Day –14 to Day 1) will be compared to tissue obtained by resection post-treatment (Day 10 to Day 14). In addition, blood and tumor obtained at the time of resection will be examined for steady state, trough plasma and intratumoral drug concentrations of lapatinib.

Changed to Read:

Breast cancer is the most common malignancy in women in the United States. Despite a variety of hormonal, cytotoxic and biologic approaches, a significant number of tumors are resistant to currently approved treatment modalities [Ring, 2002]. There is evidence that epidermal growth factor receptor (EGFR, ErbB1) and ErbB2 overexpression in breast cancers are independently associated with more aggressive tumor proliferation and a poor prognosis [Klijn, 1992; Kaptain, 2001]. ErbB1 and ErbB2 are members of the Type I family of receptor tyrosine kinases (ErbB). Tyrosine autophosphorylation of ErbB1/ErbB2 activates downstream effectors regulating cell proliferation and survival. A compound such as lapatinib that can selectively modulate the abnormal signaling pathways in these tumors is an alternative therapeutic approach in treating breast cancer. Although the efficacy of treatment with lapatinib in the neoadjuvant breast cancer setting is unknown at this time, tumor biomarker data generated from this study will guide future Phase II/III breast cancer efficacy trial design and biomarker studies.

Trastuzumab, a humanized monoclonal antibody targeting the extracellular domain of ErbB2, is effective in treating patients whose breast cancer overexpress ErbB2 and/or exhibit ErbB2 gene amplification. Lapatinib, a reversible inhibitor of both ErbB1 and ErbB2 tyrosine kinases, has been shown to induce growth arrest and/or tumor cell apoptosis in ErbB1 or ErbB2 dependent tumor cell lines or xenografts. Data from several

Phase II and III clinical studies have demonstrated the efficacy and safety of lapatinib, as either single-agent therapy or in combination with chemotherapy, in patients with ErbB2 positive, advanced breast cancer. The dual inhibitory nature of lapatinib may offer a potential therapeutic advantage over a compound that inhibits only one tyrosine receptor kinase.

This study will examine the effect of lapatinib treatment on inhibiting ErbB1, ErbB2 and downstream signaling pathways (e.g., ERK1/2 and AKT); as well as evaluate its effect on other mediators of tumor cell growth and survival in tumor tissue from treatment-naïve breast cancer patients. Patients will receive 1500mg of lapatinib treatment daily for at least 9 days or longer and continue treatment until no more than 24 hours prior to surgical resection of the tumor. Biomarker analyses will be performed on tumor tissue obtained pre-treatment (archived tumor tissue) and compared with those biomarkers derived from the tissue obtained by resection post-treatment. In addition, intratumoral drug concentrations of lapatinib will be evaluated in the tumor tissue obtained at the time of resection.

Section 1.3 Toxicology

Added Text:

An impurity 3-chloro-4-((3-fluorobenzyl) oxy) aniline has been detected in the lapatinib drug substance batches. GlaxoSmithKline does not believe the presence of this impurity increases risk for patients who are receiving, have received or will receive other therapies known to be genotoxic such as chemotherapy, hormonal therapy or radiation.

Refer to the current Supplement to the Clinical Investigator’s Brochure / Investigator’s Brochure (CIB/IB) for additional information regarding the genotoxic impurity and the results of the completed genotoxicity studies.

Section 5. Study Population

Original Text:

Male and female patients 18 years or older who are amenable to treatment with oral lapatinib will be enrolled.

Changed to read:

Female patients 18 years or older who are amenable to treatment with oral lapatinib and who will receive adjuvant chemotherapy, hormonal therapy or radiation therapy will be enrolled. An evaluable patient must meet all inclusion and exclusion criteria as detailed in Section 5.2 including taking all study medication. In addition, patients must have pre-dose and post-dose tumor tissue samples adequate for biomarker analysis. A GSK medical monitor must approve any deviation from these criteria.

Section 5.1 Number of Subjects

Original Text:

A sufficient number of patients will be enrolled to obtain 60 evaluable patients (20 per dose group) at approximately 10 study centers.

Changed to read:

A sufficient number of patients will be enrolled to obtain approximately 20 evaluable patients approximately 16 study centers. Evaluable subjects are defined as those subjects whose pre-treatment tumors are determined by a central laboratory to be ErbB2 positive, which is defined as either IHC 3+ or FISH amplified. Patients must have pre-dose and post-dose tumor tissue samples adequate for biomarker analyses, and have completed lapatinib treatment for a minimum of 9 days. In addition, the elapsed time between last lapatinib dose and surgical resection must be within 24 hours.

Section 5.2.1 Inclusion Criteria

Original Text:

1. Has a histologically confirmed, treatment-naïve, breast tumor measuring 1 cm or greater that can be readily biopsied to meet protocol requirements and that EITHER (i) expresses total ErbB1 by IHC (+), ErbB2 by IHC (2+ or 3+) and/or FISH gene amplification, (ii) or expresses activated, phosphorylated ErbB1 or ErbB2.

Changed to Read:

1. Has a histologically confirmed, treatment-naïve, breast tumor measuring 1 cm or greater.

Original Text:

3. Is male or female.

Changed to Read:

3. Has tumor that overexpresses ErbB2 defined by local laboratory as **either**:
   - 3+ by IHC

     OR
   - c-erbB2 gene amplification by FISH
   - 1+, 2+ by IHC **and** c-erbB2 gene amplification by FISH.

Original Text:

1. Is at least 18 years of age.

Changed to Read:

1. Is Female and at least 18 years of age

Added Text:

4. Will receive adjuvant chemotherapy, hormonal therapy or radiation therapy

5.2.2 Exclusion Criteria

Original Text:

14. Has a left ventricular ejection fraction (LVEF) < 40% based on MUGA or ECHO. (This criteria is based on the development of cardiomyopathy seen in patients treated with Herceptin who had prior treatment with cardiac toxic chemotherapeutic agents such as anthracyclines or taxanes).

Changed to Read:

14. Has a left ventricular ejection fraction (LVEF) < 50% based on MUGA or ECHO.

Title, Sponsor Information Page

Original Text:

A Phase I, Open Label Study of the Safety, Pharmacokinetics and Pharmacodynamics of Lapatinib (GW572016) in Once Daily Versus Twice Daily Dosing Regimens in Patients with Treatment-Naive Breast Cancer

Changed to Read:

A Phase I, Open Label Study of the Safety, Pharmacokinetics and Pharmacodynamics of Lapatinib (GW572016) in Patients with Treatment-Naïve, ErbB2 Positive Breast Cancer

Protocol Decription

Original Text:

The most commonly reported adverse events were diarrhea, rash, nausea, headache, flatus and fatigue in healthy volunteers and patients treated with once daily dosing regimens. A limited number of patients treated with lapatinib twice daily regimens, experienced an increased incidence of GI intolerance (nausea, vomiting and diarrhea). This study will examine the ability of lapatinib to inhibit downstream mediators of tumor cell growth and survival, (e.g. AKT, ERK1/2 phosphorylation and protein expression of cyclin D1) for several dosing schedules. Patients with treatment-naïve, breast tumors that express ErbB1 and/or overexpress ErbB2 protein or demonstrate ErbB2 gene amplification will be enrolled. In addition, safety and pharmacokinetic data will be collected.

Changed to Read:

The most commonly reported adverse events were diarrhea, rash, nausea, headache, flatus and fatigue in healthy volunteers and patients treated with once daily dosing regimens. This study will examine the ability of lapatinib to inhibit downstream mediators of tumor cell growth and survival (e.g. AKT and ERK1/2). Patients with treatment-naïve, breast tumors that overexpress ErbB2 protein or demonstrate ErbB2 gene amplification will be enrolled. In addition, safety and pharmacokinetic data will be collected.

Objectives, Summary and Section 2.

Primary

Original Text:

•To investigate the effects of three dosing schedules of lapatinib on intracellular mediators that regulate tumor cell growth and survival (e.g., ErbB2, ERK1/2, phosphorylated-ERK1/2, AKT, phosphorylated AKT and cyclin D1 protein and potentially other downstream markers of kinase activity)) by comparing pre-treatment and post-treatment breast tumor tissue samples.

Changed to Read:

•To investigate the effects of lapatinib on mediators that regulate tumor cell growth and survival pathways (e.g., ErbB2, ERK1/2, AKT and other downstream pathways regulated by ErbB receptor signaling) through the analysis and comparison of biomarkers derived from pre-treatment and post-treatment breast tumor tissue samples.

Secondary

Original Text:

•To assess the safety and tolerability of lapatinib at multiple dosing schedules when administered to patients with treatment-naïve breast tumors.

•To examine the steady state, trough plasma and intratumoral concentrations of lapatinib obtained at the time of tumor resection.

•To investigate the effects of lapatinib therapy on the proteomic profile and circulating extracellular domain (ECD) of ErbB1 and ErbB2 in peripheral blood.

Changed to Read:

•To assess the safety and tolerability of lapatinib when administered to patients with treatment-naïve breast tumors.

•To examine the intratumoral concentrations of lapatinib obtained at the time of tumor resection.

•To investigate the effects of lapatinib therapy on the proteomic profile in peripheral blood.

Endpoints, Summary and Section 3.

Primary

Original Text:

•Comparison of the effects of three dosing schedules of lapatinib on protein biomarkers involved in regulating tumor cell proliferation and survival ((e.g., ErbB2, ERK1/2, phosphorylated-ERK1/2, AKT, phosphorylated AKT and cyclin D1 protein and potentially other downstream markers of kinase activity)) in pre-treatment and post-treatment breast tumor tissue samples.

Changed to Read:

•Comparison of the effects of lapatinib on biomarkers and pathways involved in regulating tumor cell proliferation and survival (e.g., ErbB2, ERK1/2, AKT, and other downstream pathways regulated by ErbB receptor signaling) in pre-treatment and post-treatment breast tumor tissue samples.

Secondary

Original Text:

•Evaluation of adverse events (AEs) and changes in laboratory values from pre-dose, and post-dose values.

•Assessment of peripheral blood to examine the proteomic profile and determination of serum ErbB1 and ErbB2 (circulating extracellular domain) levels in response to lapatinib.

Changed to Read:

•Evaluation of adverse events (AEs) and changes in laboratory values from pre-dose and post-dose values.

•Assessment of peripheral blood to examine the proteomic profile in response to lapatinib.

Study Design

Original Text:

This will be a randomized, open-label study. Patients will be randomized to one of three dose groups of lapatinib, administered in a parallel design. The doses to be administered include 1000 mg and 1500 mg dosed once daily and 500 mg dosed twice daily (every 12 hours).

Prior to entering the study, the ErbB1 and/or ErbB2 status will be determined for each patient. Only patients with treatment-naïve, breast tumors measuring 1 cm or greater which express ErbB1 and/or overexpress ErbB2 by semi-quantitative IHC or demonstrate ErbB2 gene amplification by fluorescence in situ hybridization (FISH), or which express activated phosphorylated ErbB1 and/or ErbB2 by semi-quantitative IHC will be eligible for this study. For eligible patients, if archived biopsy tissue is used to determine eligibility, an additional biopsy obtained within 14 days prior to initiating study treatment will be required. The pre-treatment biopsy tissue will be examined by semi-quantitative IHC for biomarkers involved in regulating tumor cell proliferation and survival (e.g., ErbB2, ERK1/2, phosphorylated-ERK1/2, AKT, phosphorylated AKT and cyclin D1 protein and potentially other downstream markers of kinase activity). Patients will receive treatment with lapatinib for 9 to 13 days prior to surgical resection. Tumor tissue obtained at resection will be examined for biomarkers as described above and compared to the results from the pre-treatment biopsy. In addition, blood and tumor obtained at the time of resection and will be examined for steady state, trough concentrations of lapatinib.

Patients administered lapatinib once daily will have their post-treatment surgical resection performed approximately 24 hours (± 3 hours) following the previous day's dose while those administered lapatinib twice daily will have their resection or biopsy performed approximately 12 hours (± 3 hours) following the previous day's second dose to temporally coincide with the 24 hour steady state trough concentrations. Safety (AEs, clinical laboratory values) will be assessed throughout the study.

Changed to Read:

This will be a single arm, open-label phase I study. Patients will receive 1500mg of lapatinib administered daily for at least 9 days.

Prior to entering the study the ErbB2 status will be required for each patient. Only patients with treatment-naïve, ErbB2 positive, as defined by either overexpression of ErbB2 (3+) by semi-quantitative IHC or amplification of the ErbB2 gene by fluorescence in situ hybridization (FISH), breast tumors measuring 1 cm or greater will be eligible for the study. For eligible patients, the archived tumor tissue obtained at diagnosis will represent the pre-treatment biopsy and be required for entry into the study. The pre-treatment biopsy tissue will be examined using technologies for measuring biomarkers involved in regulating tumor cell proliferation and survival (e.g., ErbB2, ERK1/2, AKT, and other downstream pathways regulated by ErbB receptor signaling). Patients will receive treatment with lapatinib for a minimum of 9 days prior to surgical resection. Patients will have their post-treatment surgical resection performed within 24 hours of last lapatinib dose. Tumor tissue obtained at resection will be examined for biomarkers as described above and compared with the results from the pre-treatment biopsy. In addition, tumor obtained at the time of resection will be examined for steady state, trough concentrations of lapatinib.

Safety (AEs, clinical laboratory values) will be assessed throughout the study.

Study Assessments and Procedures

Original Text:

The pre-treatment tumor biopsy sample may be obtained within fourteen days prior to initiating dosing with lapatinib. If archived biopsy tissue is used for determining eligibility, an additional biopsy obtained within 14 days prior to initiating study treatment will be required. Three core needle biopsies will be obtained.

Patients will receive study medication for 9 to 13 days (until the day prior to scheduled surgical resection of the tumor). Surgical resection will coincide with the lapatinib trough concentration. A minimum of 250 mg of tumor tissue (approximately equivalent to 3 core needle biopsies) is required for biomarker and lapatinib intratumoral concentration analysis.

Changed to Read:

The archived tumor biopsy obtained for diagnosis of this incidence of breast cancer must be available and shipped to a GSK Central lab for biomarker studies. Patients will receive study medication for a minimum of 9 days and continue treatment until scheduled surgical resection of the tumor (subjects must continue lapatinib treatment so that no more than 24 hours have elapsed between last dose and surgical resection). Surgical resection will coincide with the lapatinib trough concentration. The resected tumor tissue is required for biomarker and lapatinib intratumoral concentration analyses.

Investigational Product(s)

Removed Text:

the dose group assignment and

Section 1.4 Preliminary Results from Healthy Volunteers and Patients

Added Text:

The efficacy and safety of lapatinib as a single agent or in combination with chemotherapy are being assessed in several clinical studies of metastatic breast cancer.

Single agent lapatinib exhibited a 6% clinical benefit response rate in a heavily pre-treated population where an unmet medical need exist [GSK Document Number RM2005/00018/00] (Study EGF20008). In a large, randomized, Phase III study EGF100151 [GSK Document Number ZM2006/00137/00], lapatinib plus capecitabine demonstrated anti-tumor activity in patients with refractory, advanced metastatic breast cancer (MBC) who were previously treated with trastuzumab. Data from the pivotal study EGF100151 supported approval of lapatinib plus capecitabine for the treatment of patients with HER2-positive advanced or MBC who have received prior anthracycline, taxane and trastuzumab therapy [TYKERB Package Insert, 2007]. In this trial, lapatinib 1250mg once daily (QD) plus capecitabine 2000 mg/m2/day on days 1–14 every 3 weeks

showed a statistically and clinically significant improvement in median time to progression of disease compared with capecitabine (2500 mg/m2/day on days 1–14 every 3 weeks) alone (hazard ratio 0.57; p=0.00013; median of 27.1 in the combination arm and 18.6 weeks in the control arm).

Section 1.5 Rationale

Original text:

Although the efficacy of treatment with lapatinib in the neoadjuvant breast cancer setting is unknown at this time, tumor biomarker data generated from this study will guide future Phase II/III breast cancer efficacy trial design.

FDA approval of Herceptin, a humanized monoclonal antibody targeting ErbB2 effective in treating patients whose breast cancer over-express ErbB2 and/or display ErbB2 gene amplification, demonstrates the clinical utility of ErB2 targeted therapy. Lapatinib, a reversible inhibitor of both ErbB1 and ErbB2 tyrosine kinases has been shown to induce growth arrest and/or tumor cell apoptosis in ErbB1 or ErbB2 dependent tumor cell lines or xenografts. The dual inhibitory nature of lapatinib offers a potential therapeutic advantage over a compound that inhibits only one tyrosine receptor kinase.

This study will examine the inhibition of ErbB1 and ErbB2 phosphorylation and downstream mediators of tumor cell growth and survival (e.g., phosphorylated ERK1/2, phosphorylated AKT and cyclin D1 protein and potentially other downstream markers of kinase activity) tumor tissue in treatment-naïve breast cancer patients for three dosing schedules of lapatinib. Patients will be randomized to one of the three dose groups of lapatinib and will receive treatment for 9 to 13 days prior to surgical resection of the tumor. Biopsy tissue obtained pre-treatment (Day –14 to Day 1) will be compared to tissue obtained by resection post-treatment (Day 10 to Day 14). In addition, blood and tumor obtained at resection will be examined for steady state, trough plasma and intratumoral drug concentrations of lapatinib.

Changed to Read:

Although the efficacy of short-term treatment with lapatinib prior to surgical resection in breast cancer is unknown at this time, tumor biomarker data generated from this study will guide the design of future Phase II/III breast cancer efficacy trials and biomarker studies.

Trastuzumab, a humanized monoclonal antibody targeting ErbB2, is effective in treating patients whose breast cancer overexpress ErbB2 and/or display ErbB2 gene amplification, and hence, demonstrates the clinical utility of ErbB2 targeted therapy. Lapatinib, a reversible inhibitor of both ErbB1 and ErbB2 tyrosine kinases has been shown to induce growth arrest and/or tumor cell apoptosis in ErbB1 or ErbB2 dependent tumor cell lines or xenografts. The dual inhibitory nature of lapatinib offers a potential therapeutic advantage over a compound that inhibits only one tyrosine receptor kinase.

This study will examine the effect of lapatinib treatment on inhibiting ErbB1, ErbB2 and downstream signaling pathways (e.g., ERK1/2 and AKT); as well as evaluate its effect on other mediators of tumor cell growth and survival in tumor tissue from treatment-naïve breast cancer patients. Patients will receive 1500mg of lapatinib treatment daily for at least 9 days or longer and continue treatment until no more than 24 hours have elapsed prior to surgical resection of the tumor. Biomarker analyses will be performed on tumor tissue obtained pre-treatment (archived tumor tissue which is required for entry into the study) and compared with those biomarkers derived from the tissue obtained by resection post-treatment. In addition, intratumoral drug concentrations of lapatinib will be evaluated in the tumor tissue obtained at the time of resection.

Section 4. Study Design

Original Text:

This will be a randomized, open-label study. Patients will be randomized to one of three dose groups of lapatinib, administered in a parallel design as shown in Table 1 below. The doses to be administered include 1500 mg and 1000 mg dosed once daily and 500 mg dosed twice daily (every 12 hours).

Prior to entering the study, the ErbB1 and/or ErbB2 status will be determined for each patient.. Patients with treatment-naïve breast tumors measuring 1 cm or greater which express ErbB1 (+) and/or overexpress ErbB2 (2+ or 3+) by semi-quantitative IHC or demonstrate amplification of the ErbB2 gene by fluorescence in situ hybridization (FISH), or which express activated phosphorylated ErbB1 and/or ErbB2 determined by semi-quantitative IHC will be eligible for this study. For eligible patients, if archived biopsy tissue is used to determine eligibility, an additional biopsy obtained within 14 days prior to initiating study treatment will be required. The pre-treatment biopsy tissue will be examined by semi-quantitative IHC for biomarkers involved in regulating tumor cell proliferation and survival (e.g., ErbB2, ERK1/2, phosphorylated-ERK1/2, AKT, phosphorylated AKT and cyclin D1 protein and potentially other downstream markers of kinase activity). Patients will receive treatment with lapatinib for 9 to 13 days prior to surgical resection.Tumor tissue obtained at resection will be examined for biomarkers as described above and compared to the results from the pre-treatment tumor sample. In addition, blood and tumor obtained at the time of resection and will be examined for steady state, trough concentrations of lapatinib.

Patients administered lapatinib once daily will have their post-treatment surgical resection performed approximately 24 hours (± 3 hours) following the previous day's dose while those administered lapatinib twice daily will have their resection performed approximately 12 hours (± 3 hours) following the previous day's second dose to temporally coincide with the 24 hour steady state trough concentrations. Safety (AEs, clinical laboratory values) will be assessed throughout the study.

Changed to Read:

This will be a single arm, open-label phase I study. Patients will receive 1500mg of lapatinib administered daily for a minimum of 9 days and continue treatment until surgical resection (≤24 hours).

Prior to entering the study, the documented ErbB2 status will be required for each patient. Only patients with treatment-naïve, ErbB2 positive, as defined by either overexpression of ErbB2 (3+) by semi-quantitative IHC or amplification of the ErbB2 gene by fluorescence in situ hybridization (FISH), breast tumors measuring 1 cm or greater will be eligible for the study. For eligible patients, archived tumor tissue obtained at diagnosis will represent the pre-treatment biopsy and be required for entry into the study. The pre-treatment biopsy tissue will be examined using technologies for measuring biomarkers involved in regulating tumor cell proliferation and survival (e.g., ErbB2, ERK1/2, AKT, and other downstream pathways regulated by ErbB receptor signaling). Patients will receive treatment with lapatinib for a minimum of 9 days and continue treatment until 24 hours prior to surgical resection. Patients will have their post-treatment surgical resection performed within 24 hours of last lapatinib dose. Tumor tissue obtained at resection will be examined for biomarkers as described above and compared with the results from the pre-treatment biopsy. In addition, tumor obtained at the time of resection will be examined for steady state, trough concentrations of lapatinib.

Detailed instructions on processing and shipping archived and resected tissue are included in the SRM.

Safety (AEs, clinical laboratory values) will be assessed throughout the study.

Removed:

Table 1 Lapatinib Randomized Treatment Assignment

Section 5.2.3.Other Eligibility Criteria Considerations

Removed Text:

•Patients must fast 1 hour prior to and post dosing from Day 1 through Day 9 to 13 depending on date of scheduled resection with the exception of water which will be allowed freely.

Section 6.3. On Study Assessments

Removed Text throughout section:

(Day 9 to 13)

Original Text:

•A blood sample for pharmacokinetic analysis will be collected pre-dose on Day 1 and at the time of surgical resection on Day 10 to 14. Refer to Section 6.6 for sampling schedule.

Changed to Read:

•A blood sample for pharmacokinetic analysis will be collected pre-dose on Day 1 and at the time of surgical resection. Refer to Section 6.6 for sampling schedule.

Original Text:

•Tumor surgical resection (Day 10 to 14).

Changed to Read:

•Tumor surgical resection (≥Day 10).

Section 6.5.1.1.Pregnancy testing

Removed Text:

, Day 1 and at the 28 day post study follow-up visit

6.5.1.2.Time period for collecting pregnancy information

Removed Section

Section 6.5.3 Cardiac Assessments

Original Text Title:

Electrocardiogram and MUGA or ECHO

Changed to Read:

Cardiac Assessments

Added Text:

6.5.3.1. Primary Cardiac Endpoint

The following is a definition of a primary cardiac endpoint, which includes cardiac death and congestive heart failure. This definition is provided to universally describe cardiac-related events.

- Cardiac death defined as either:
- Cardiac death due to heart failure, myocardial infarction or arrhythmia;
- Probable cardiac death defined as sudden, unexpected death within 24 hours of a definite or probable cardiac event.
- Severe symptomatic congestive heart failure defined as:
- New York Heart Association (NYHA) class III or IV (refer to Appendix5) (class III defined as being not capable of climbing one flight of stairs and class IV defined as having symptoms at rest), and
- A drop in left ventricular ejection fraction (LVEF) of more than 10 points from baseline and below 50%.

Treatment with lapatinib will be permanently stopped if a subject develops severe symptomatic NYHA class III or IV event and has a drop in LVEF of more than 10 points. However, all efforts must be made to complete all post-study patient assessments which must include LVEF measurement. The method for LVEF assessment at screening should be used to assess LVEF in 4 weeks after documented absolute decrease of 10 points from baseline, and every 4 weeks for at least 16 weeks or until resolution.

6.5.3.2. Secondary Cardiac Endpoint

- Asymptomatic or mildly symptomatic cardiac event defined as:
- An asymptomatic (NYHA I) or mildly symptomatic (NYHA II) significant decrease in LVEF defined as an absolute decrease in LVEF of more than 10 points from baseline and
- LVEF value below 50%.

A second LVEF assessment must be performed within approximately 3 weeks to confirm the significant decrease in LVEF as defined above. If a subject has a confirmed secondary cardiac event as defined above, cardiac evaluation, preferably using the same method as for LVEF baseline assessment, must be performed every 4 weeks for at least 16 weeks or until resolution.

Section 6.5.5. Criteria for Study Hold

Original text:

If Grade III or IV toxicity is observed in 30% (3 patients) of the first 10 patients in each dosing group, the study will placed on hold (ie. no further accrual). The safety data will be reviewed jointly by the GSK medical monitor and the Principal Investigators to determine if termination of additional enrollment in the given lapatinib dosing group is warranted.

Changed To Read

If Grade III or IV toxicity is observed in 30% (3 patients) of the first 10 patients the study will placed on hold (ie. no further accrual). The safety data will be reviewed jointly by the GSK medical monitor and the Principal Investigators to determine if termination of additional enrollment is warranted.

Section 6.6. Pharmacokinetics

Removed Text:

(Day 10 to 14)

Section 6.7.Pharmacodynamics

Original Text:

Blood samples (2 x 5 mL tubes) will be obtained pre-treatment (Day 1) and on the day prior to surgical resection (Day 9 to 13) to evaluate changes in circulating/secreted proteins (proteomic analysis) and biomarkers (circulating extracellular domain [ECD] concentrations of ErbB1 and ErbB2) that may correlate with biological response at the tumor site and therefore provide an easily obtainable surrogate marker for determining therapeutic benefit of lapatinib. An additional 5 mL of blood will be obtained at the Post Study Visit to examine ECD concentrations of ErbB1 and ErbB2.

Changed to Read:

Blood samples (2 x 5 mL tubes) will be obtained pre-treatment (Day 1) and on the day prior to surgical resection to evaluate changes in circulating/secreted proteins (proteomic analysis) that may correlate with biological response at the tumor site and therefore provide an easily obtainable surrogate marker for determining therapeutic benefit of lapatinib. An additional 5 mL of blood will be obtained at the Post Study Visit to examine the changes in circulating/secreted proteins identified from the pharmacodynamic analyses.

Section 6.7.1.Tumor Tissue Samples

Original Text:

Tumor biopsy tissue obtained within 14 days prior to initiating dosing with lapatinib will be collected. Three core needle biopsies will be required for biomarker and pharmacokinetic analysis.

On Day 10 to 14, patients administered lapatinib once daily will have a post-treatment surgical resection performed approximately 24 hours (± 3 hours) following the previous day’s dose while those administered lapatinib twice daily will have their surgical resection performed approximately 12 hours (± 3 hours) following the previous day’s second dose to temporally coincide with the 24 hour steady state trough concentrations. A

minimum of 250 mg of tumor is required for biomarker and intratumoral lapatinib concentration analysis. If for any reason, surgical resection cannot be performed within the time period from Day 10 to 14, the GSK medical monitor must be contacted.

Changed to Read:

Archived tumor tissue obtained at diagnosis (prior to initiating dosing with lapatinib) is required for biomarker analyses. All of the archived tumor tissue is required for pre-treatment biomarker analysis.

Patients will have a post-treatment surgical resection performed within 24 hours of lapatinib dose. If for any reason, surgical resection cannot be performed within 24 hours after the last dose of lapatinib, the GSK medical monitor must be contacted.

Section 6.8.Pharmacogenetics

Removed Text:

his or her

Section 6.8.6 Pharmacogenetic Samples

Original Text:

In addition to any blood samples taken for the clinical study, a whole blood sample (~10ml) will be collected for the PGx research using a tube containing EDTA. The PGx sample is labeled (or "coded") with a study specific number that can be traced or linked back to the subject by the investigator or site staff. Coded samples do not carry personal identifiers (such as name or social security number). The blood sample will be taken on a single occasion unless a duplicate sample is required due to inability to utilize the original sample. It is recommended that the blood sample be taken at the first available opportunity, but may be taken at any time while the subject is participating in the clinical study.

The whole blood sample must be shipped to the receiving laboratory at room temperature on the day of collection. A freezer log should be maintained at the site capturing the time of sample collection and the time of sample storage in the freezer. Also freezer temperature needs to be maintained to confirm appropriate storage conditions.

The sample can be shipped ambient provided it is shipped the same day the sample is drawn.

Prior to sample shipment, the site staff should contact the sponsor staff member responsible for the study to notify them of the impending shipment. A completed inventory form must accompany the samples to the laboratory. In order to avoid the potential for sample degradation, samples should only be sent Monday through Wednesday, unless alternative arrangements have been agreed to by the receiving laboratory.

The inventory form will contain the following:

- study identifier
- investigator's name
- site address
- site number
- site and sponsor contact names and telephone numbers
- subject number(s)
- date(s) of sample collection(s)
- shipment date

Changed to Read

In addition to any blood samples taken for the clinical study, a whole blood sample (~10ml) will be collected for the PGx research using a tube containing EDTA. The PGx sample is labeled (or "coded") with a study specific number that can be traced or linked back to the subject by the investigator or site staff. Coded samples do not carry personal identifiers (such as name or social security number). The blood sample will be taken on a single occasion unless a duplicate sample is required due to inability to utilize the original sample. It is recommended that the blood sample be taken at the first available opportunity, but may be taken at any time while the subject is participating in the clinical study.

The whole blood sample must be shipped to the receiving laboratory at room temperature on the day of collection. A log should be maintained at the site capturing the time of sample collection.

Prior to sample shipment, the site staff should contact the sponsor staff member responsible for the study to notify them of the impending shipment. A completed inventory form must accompany the samples to the laboratory.

The inventory form will contain the following:

- study identifier
- investigator's name
- site address
- site number
- site and sponsor contact names and telephone numbers
- subject number(s)
- date(s) of sample collection(s)
- shipment date
- total number of tubes

- number of tubes/subject
- barcode (if applicable)
- comments (e.g., environmental conditions, information on broken/missing tubes)
- signature of packer

Section 7.1. Description of Investigational Product

Original Text:

The number of tablets and administration schedule will be dependent upon the dose randomization assignment and the number of days between enrollment and scheduled surgical resection.

Changed to Read:

The number of tablets and administration schedule will be dependent upon the number of days between enrollment and scheduled surgical resection.

Section 7.2. Dosage and Administration

Removed Table

Original Text:

Patients will be randomized to one of 3 dose groups as detailed below. A randomized treatment assignment will be generated and supplied to each participating study center by GSK.

Twice daily lapatinib doses are to be spaced by 12 hours. Patients must take lapatinib on an empty stomach (1 hour prior to and following food intake).

Changed to Read:

A daily dose of lapatinib is six tablets (1500mg of lapatinib) taken approximately at the same time each day. Subjects will be instructed to take study drug either 1 hour (or more) before a meal or 1 hour (or more) after a meal.

Section 7.3. Dose Rationale

Original Text:

Based on pharmacodynamic, clinical response, and safety data from studies EGF10003 and EGF10004 (see below), the doses to be examined in the randomized portion of this study will be 1500 mg and 1000 mg once daily and 500 mg twice daily.

Tumor biomarker changes and clinical responses were observed in study EGF10004 at doses of lapatinib ranging from 650 mg to 1600 mg once daily. The frequency of

achieving a ≥75% inhibition of one of four key biomarkers (p-EGFR, p-ErbB2, p-ERK1/2, or p-AKT) was 29% at 500mg, 50% at 650mg, 44% at 900mg, 75% at 1200mg, and 83% at 1600mg once daily. Total daily doses of 1000 mg and 1500 mg daily appear to be in the active range as both biologic and clinical responses have been observed. Study EGF10003 examined three different twice daily doses, 900mg BID, 750mg BID, and 500mg BID. Dose-limiting toxicity (diarrhea) was observed at the 900mg BID dose (2 of 6 subjects) and preliminary data suggests the 750mg BID dose is poorly tolerated by patients (although no DLT has been observed at 750mg BID). Twice daily dosing also results in significant increases in exposure (AUC ~two-fold higher in 900 mg BID dosing compared with 1800 mg once daily). Thus, the 500 mg BID dose would be expected to have increased exposure compared with 1000 mg once daily, but variability in the pharmacokinetic profiles makes dosing based on exposure difficult. Therefore, the doses were chosen based observations of clinical and biologic response as well as tolerability. lapatinib is currently available as a 250 mg tablet (not scored).

The doses in this study will allow assessment of tumor response (i.e. inhibition of downstream mediators of tumor cell growth and survival) in lapatinib dosing regimens of 1500 mg and 1000 mg once daily and 500 mg twice daily.

Changed to Read:

The dose of lapatinib to be tested in this study is 1500 mg QD and was selected based upon the following criteria:

- Cell-based assays demonstrate that the lapatinib concentration required to inhibit the proliferation of either ErbB1 or ErbB2 over-expressing tumor cell lines by 90% (IC_{90}) ranged from 520ng/mL to 1313ng/mL, depending upon the cell line. These concentrations were associated with 90% inhibition of tumor cell proliferation and comparable inhibition of ErbB1 or ErbB2 receptor tyrosine phosphorylation.
- In the initial Phase I patient study (EGF10003), lapatinib doses of 1200 mg and 1600 mg QD were well tolerated with only Grade 1/2 diarrhea and skin rash reported.
- Initial biopsies of skin, which expresses ErbB1 in the epidermis, show that doses below 1200 mg QD inhibit ErbB tyrosine autophosphorylation, indicating that 1500 mg QD is within in the biologically active dose range.
- Preliminary analysis of EGF10004 indicates that doses of 1200 mg QD produced biological activity against growth and survival pathways in tumor biopsies. Data showed a plateau of effects on biomarkers (pErbB1, pErbB2, pAKT, and pERK) between doses 1200 mg and 1500 mg daily. No maximum tolerated dose was reached at the maximum dose given (1800 mg/day). Therefore, to ensure that patients received the optimum effect from lapatinib, the dose of lapatinib was increased from 1250 to 1500 mg/day, with the expectation that this change was unlikely to affect the safety profile.
- Pharmacokinetic data in patients receiving lapatinib doses up to 1800 mg QD indicate that QD dosing results in 1.5- to 2-fold accumulation and 3-fold fluctuation between peak and trough plasma concentrations at steady state. Based on these data, 1500 mg QD is likely to produce a steady-state plasma concentration profile with

peaks that are above, and troughs that are below these *in vitro* IC90. Although steady-state trough concentrations at this dose may not exceed these *in vitro* IC90, data from animal xenografts indicate that intra-tumoral concentration exceeds and lags behind plasma concentration. Therefore, declining plasma concentrations in patients may not preclude continuous exposure in tumors with QD dosing.

Section 7.4. Treatment Assignment

Original Text:

Patients will be assigned to study treatment in accordance with the randomization schedule provided by GSK. Each site will receive a randomization dose assignment. The randomization number and corresponding dose should be assigned to patients qualified for enrollment in a sequential manner. Once a patient treatment allocation has been assigned, it cannot be reassigned to any other patient.

Any patient not completing all required assessments will be replaced. Replacement patients will receive the next available patient number.

Changed to Read:

All patients will receive 1500 mg of lapatinib daily. Any patient not completing all required assessments will be replaced.

Section 8.2. Prohibited Medications

Removed Text:

All H2 Blockers and Proton Pump Inhibitors to allow for their use while on treatment

Section 11.1. Hypotheses

Original Text:

No formal hypotheses are being tested. The study will focus on obtaining point estimates and 90% confidence intervals of differences in pre-to-post optical density changes in tumor tissue markers for 3 different dosing regimens.

Changed to Read:

No formal hypotheses are being tested. The study will focus on obtaining point estimates and 90% confidence intervals of biomarker differences derived from the pre-to-post tumor tissue.

Section 11.2. Sample Size Considerations

Original Text:

Subjects will be randomized in equal numbers to the three dosing schedules. Twenty evaluable subjects are targeted for each schedule.

Changed to Read:

Twenty evaluable subjects are needed for biomarker analyses.

Section 11.2.1. Sample Size Assumptions

Removed Text:

of each treatment group

Section 11.3. Analysis Population

Removed Text:

to 13

Section 11.4.1. Withdrawal

Original Text:

Patients that drop out will be replaced at the same dose and schedule wherever possible.

Changed to Read:

Patients that drop out will be replaced until 20 subjects have been determined to be evaluable.

Section 11.6.2. Pharmacodynamic Analyses

Original Text:

Each patient will be measured for biomarker activity level in tumor obtained pre- and post-dose. For each marker, a one-way analysis of covariance of the post-dose measurement will be performed with treatment group as a classification variable and the pre-dose level of the marker as a covariate. Adjusted least squares means will be estimated for each dose regimen. Tansformation, e.g., logarithms, of pre- and post-dose marker levels may be necessary to satisfy analysis asssumptions.

Analysis of proteomic data will be the responsibility of the Discovery Bioinformatics (DB). Statistical analyses of the proteomic data will be the responsibility of Clinical Pharmacology Statistics and Data Sciences, and will be performed in consultation with

DB. All pharmacodynamic endpoints of interest from all study sessions will be descriptively and/or graphically summarized, as appropriate to the data.

Changed to Read:

Each patient will be measured for biomarker activity level in tumor obtained pre- and post-dose. For each marker, a one-way analysis of covariance of the post-dose measurement will be performed with a biological marker of cellular proliferation, cellular apoptosis, or other biological marker deemed relevant to pathways regulating cancer pathophysiology as a classification variable and the pre-dose level of the marker as a covariate. Transformation, e.g., logarithms, of pre- and post-dose marker levels may be necessary to satisfy analysis asssumptions.

All pharmacodynamic endpoints of interest from all study sessions will be descriptively and/or graphically summarized, as appropriate to the data.

Section 14.1. Appendix 1 Time and Events Table

Original Column Name:

Day 10 to 14

Changed to Read:

Day 10 Until Resection

Removed Text:

to 13; ±3 days; If archived tissue used to determine elgibility; an additional biopsy obtained within 14 days of initiating study treatment will be required.

Original Text in table footnotes:

a. ErbB1/ErbB2 expression results must be available prior to dosing on Day
c. LVEF must be >40% by MUGA or ECHO to be eligible for enrollment in the study.
f. The pre-dose tumor biopsy must be obtained within 14 days of initiating study treatment. The post-treatment tumor resection must coinicide with the previous day's lapatinib dose trough concentration ± 3 hours.

Changed to Read:

a. Local lab ErbB2 results must be available prior to dosing on Day 1.
c. LVEF must be ≥50% by MUGA or ECHO to be eligible for enrollment in the study.
f. The archived tumor biopsy which is equivalent to the pre-dose tumor biopsy must be obtained prior to initiating study treatment. The post-treatment tumor resection must coinicide with the previous day's lapatinib dose trough concentration and must be collected within 24 hours of last lapatinib dose.

Section 14.5. Appendix 5 New York Heart Association Functional Classification

Added this Appendix and renumbered subsequent appendices
