# Supplementary material for: Lapatinib Plasma and Tumor Concentrations and Effects on HER Receptor Phosphorylation in Tumor
Source: PLoS One. 2015 Nov 16;10(11):e0142845. doi: 10.1371/journal.pone.0142845 (PMC4646457; doi:10.1371/journal.pone.0142845)
Supplement: S1 Table — Lapatinib was administered to fed female tumor-bearing CB-17 SCID mice orally to ascertain the pharmacokinetics and pharmacodynamics of its free base. Blood, kidney, liver and BT474 tumor samples were collected up to 144 hours after a single dose or multiple doses (100, and 200 mg/kg BID or QD for 3 days). Plasma and tissue samples were analyzed for lapatinib concentration by LC/MS/MS. Values shown are based on the calculated mean. Mean values for Tmax (time to maximum concentration) and MRTlast (mean resonance time) are indicated in hours, while Cmax (maximum concentration) and AUC (area under the curve) are indicated in ng/ml. (PDF) [file pone.0142845.s005.pdf]

	Dose (mg/kg)	T_{max} (h)		C_{max} (ng/mL)		AUC_{last} (h.ng/mL)		MRT_{last} (h)	
		Single	Multiple	Single	Multiple	Single	Multiple	Single	Multiple
**Blood**	100	1	1	7040	9347	46713	101828	6.3	6.9
	200	12	10	8217	16075	88155	151764	8.5	7.3
**Kidney**	100	1	10	29774	98540	266736	1167077	7.7	8.4
	200	12	10	64863	135920	632965	1317909	9.0	8.0
**Liver**	100	1	10	46696	118705	414284	1382325	7.5	8.0
	200	12	10	109752	240442	1051905	2106717	9.4	8.4
**Tumor**	100	12	4	24900	55806	313254	1118385	12.6	20.5
	200	1	10	44046	79220	578794	1320952	17.5	18.7
