# Supplementary material for: Lapatinib Plasma and Tumor Concentrations and Effects on HER Receptor Phosphorylation in Tumor
Source: PLoS One. 2015 Nov 16;10(11):e0142845. doi: 10.1371/journal.pone.0142845 (PMC4646457; doi:10.1371/journal.pone.0142845)
Supplement: S2 Table — Data were from female mice after single and repeat oral administration at 100 mg/kg BID, or 200 mg/kg QD. (PDF) [file pone.0142845.s006.pdf]

Dose (mg/kg)	Time (h)	Animals 1	Animals 2	Animals 3	Mean (ng/ml)	SD (ng/ml)
**Blood**						
100 BID	1	10959	9673	7410	9347	1796
	4	7047	6787	10981	8272	2350
	10	3082	8918	11638	7880	4372
	24	17	95.2	43.2	51.8	39.8
	48	BQL	17	BQL	5.68	9.83
	72	BQL	BQL	BQL	BQL	BQL
	96	ND	ND	ND	BQL	BQL
	144	ND	ND	ND	BQL	BQL
200 QD	1	6512	7727	14360	9533	4224
	4	6915	17523	10263	11567	5423
	10	4796	20680	22750	16075	9823
	24	28	BQL	16.9	15	14.1
	48	BQL	BQL	11.5	3.8	6.6
	72	BQL	BQL	BQL	BQL	BQL
	96	ND	ND	ND		
	144	ND	ND	ND		
**Tumor**						
Dose (mg/kg)	Time (h)	Animals 1	Animals 2	Animals 3	Mean (ng/ml)	SD (ng/ml)
100 BID	1	11837	4179	11995	9337	4467
	4	25281	51373	90764	55806	32966
	10	59243	64621	35943	53269	15243
	24	5497	20022	7578	11032	7854
	48	5900	8812	4287	6333	2293
	72	2060	1135	944	1380	597
	96	108	928	345	460	422
	144	113	118	118	116	3
200 QD	1	40087	18597	33094	30593	10961
	4	47375	78459	83131	69655	19436
	10	25977	71794	139890*	79220	57318
	24	9372	3931	5937	6413	2751
	48	7573	2009	14505	8029	6261
	72	853	1778	1611	1414	493
	96	226	710	915	617	354
	144	104	162	205	157	50.7
**Kidney**						

Dose (mg/kg)	Time (h)	Animals 1	2	3	Mean (ng/ml)	SD (ng/ml)
	1	57250	89224	56923	67799	18555
	4	72318	76828	117987	89044	25166
	10	61132	105982	128507	98540	34298
100	24	1813	1188	841	1281	493
BID	48	114	422	166	234	165
	72	94.5	75.3	55.2	75	19.6
	96	38.6	47.9	41.2	42.6	4.8
	144	46.6	37	51.7	45.1	7.46
	1	48267	48559	81791	59539	19271
	4	69496	152726*	99172	107131	42182
	10	217197*	139734	50830	135920	83249
200	24	267	144	254	222	67.8
QD	48	229	152	264	215	57.2
	72	77.3	112	111	100	19.6
	96	49.9	56.9	50.4	52.4	3.92
	144	33.1	31	71	45	22.5
**Liver**						
Dose (mg/kg)	Time (h)	Animals 1	2	3	Mean (ng/ml)	SD (ng/ml)
	1	106815	89306	90217	95446	9856
	4	90677	96684	137136	108166	25268
	10	55647	136587	163881*	118705	56289
100	24	279	1323	703	769	525
BID	48	193	333	213	247	75.8
	72	121	101	104	109	11.1
	96	66.9	83	82.7	77.5	9.22
	144	78	52.9	88.8	73.2	18.4
	1	72240	74786	136615	94547	36454
	4	80867	223450*	144989	149769	71412
	10	420671*	236852*	63803	240442	178461
200	24	427	159	293	293	134
QD	48	228	173	250	217	39.3
	72	101	860	109	356	436
	96	81.9	98.3	86.3	88.9	8.46
	144	46.6	50.9	120	72.5	41.2

Abbreviations: BID: twice a day; BQL: Below the quantitation limit (20 ng/mL); QD: once a day; ND: Not Determined; SD: Standard Deviation.
*The sample concentration was above the limit of quantitation.
