# Supplementary material for: Lapatinib Plasma and Tumor Concentrations and Effects on HER Receptor Phosphorylation in Tumor
Source: PLoS One. 2015 Nov 16;10(11):e0142845. doi: 10.1371/journal.pone.0142845 (PMC4646457; doi:10.1371/journal.pone.0142845)
Supplement: S3 Table — (PDF) [file pone.0142845.s007.pdf]

	Lap conc (µM)		p-EGFR			p-HER2			p-HER3		
SUBJ	Plasma	Tumor	post	pre	post/pre	post	pre	post/pre	post	pre	post/pre
1401	0.16	1.02	6	0	*	---	---	---	3	16	0.19
801	0.24	1.98	6	7	0.86	---	---	---	14	15	0.93
803	0.53	2.08	4	4	1.00	---	---	---	5	9	0.56
806	0.32	2.27	5	0	*	9	12	0.75	8	6	1.33
304	0.39	2.39	7	13	0.54	---	---	---	22	20	1.10
804	3.12	7.90	6	5	1.20	---	---	---	11	7	1.57
1403	0.93	9.08	0	0	**	0	7	**	0	12	**
1405	2.75	12.76	7	7	1.00	17	12	1.42	18	13	1.38
5	1.97	25.60	13	7	0.54	10	15	0.67	16	22	0.73
124	2.29	56.02	8	8	1.00	---	---	---	7	16	0.44
SD			3.26	4.23	0.25	6.98	3.32	0.41	7.04	5.27	0.47
Mean			6.20	5.10	0.88	9.00	11.50	0.94	10.40	13.60	0.91
CV			0.526	0.829	0.287	0.775	0.288	0.435	0.677	0.388	0.516

* pre-treatment zero (maximal increase) ratio = 2

** post-treatment zeros (minimal increase) ratio = 0

Abbreviations: SUBJ:Subject; p-EGFR: phosphorylated EGFR; p-HER2:phosphorylated HER2; p-HER3:phosphorylated HER3; post: post-treatment; pre: pre-treatment; post/pre: ratio of post to

pre-treatment optical density values; CV: coefficient of variation; OD: optical density; SD: standard deviation
